# Developing a South African curriculum for education in neonatal critical care retrieval: An initial exploration

**DOI:** 10.1371/journal.pone.0290972

**Published:** 2023-08-31

**Authors:** W. Williams, E Theron, W Khan, W Stassen

**Affiliations:** Division of Emergency Medicine, University of Cape Town, Cape Town, South Africa; University of Sharjah, UNITED ARAB EMIRATES

## Abstract

**Background:**

Owing to limited or centralised neonatal critical care resources, the interfacility transfer of neonates is inevitable. In many high-income settings, dedicated Critical Care Retrieval Services (CCRS) with additional education and training undertake neonatal critical care retrieval (CCR). In South Africa, however, these transfers are mostly conducted by advanced paramedics with limited education in neonatal care, and this may lead to high adverse event rates. In SA, a shortage of skilled neonatal interfacility transport services has been identified as one of the top ten avoidable causes of under-5 mortality. In order to address this gap in neonatal transfer education for paramedics in South Africa, the aim of this study is to develop a curriculum for neonatal critical care retrieval in South Africa.

**Methods:**

Using Kern’s approach to curriculum development, a general and targeted needs assessment was conducted through semi-structured interviews with experts in the field and a focus group discussion with a prospective student group. Interviews were preceded and informed by a literature review and retrospective chart review of neonates who underwent CCR in SA over a one-year period. Audio recordings of interviews were transcribed verbatim and subjected to inductive-dominant content analysis. Finally, qualitative codes were expanded into course outcome and a curriculum map was developed.

**Results:**

Six experts in neonatal critical care and retrieval participated in semi-structured interviews with a mean duration of 59 minutes. Following transcription and analysis, 372 codes were developed. Seven prehospital providers (prospective students) who are involved in neonatal transfers in South Africa participated in a focus group discussion with a duration of 91 minutes. The audio recording was transcribed and analysed with 97 codes extracted. The main categories were: Current status of neonatal CCR in South Africa; learning and education in neonatal CCR; and proposed curriculum structure. The proposed curriculum structure described 13 broad course outcomes to be delivered as a blended postgraduate programme. Participants noted that funding, employer buy-in and internet resources would be required. The targeted prospective student group should be all Advanced Life Support (ALS) providers with a change in their scope of practice on completion.

**Conclusion:**

This study described the need for additional education in neonatal critical care retrieval due to the limitations in the current and past education systems. This study provides a curriculum structure with course outcomes that can be used as a basis for the development of a complete curriculum for education in neonatal CCR, with the potential to greatly reduce adverse event rates.

## Background

Owing to limited neonatal critical care resources, the transfer of neonates to district and tertiary facilities is inevitable in Low- and Middle-Income Countries (LMICs), such as South Africa. Lack of appropriate interfacility transport has been identified as one of the top ten avoidable causes of under-5 mortality in South Africa [1]. The physiological stressors experienced by the neonate during transport may lead to clinical deterioration or instability, making transfer inherently high risk in nature [2–9]. A lack of specialised equipment [2, 5, 6, 8, 10–12] and time delays for transfer and stabilisation [2, 6, 9–11, 13–15] have all been associated with an increased incidence of adverse events. Similarly, the level of education and training of the transferring provider has been shown to be one of the biggest predictors of adverse events and eventual patient outcome [2, 5–11, 13, 14]. Internationally, neonatal transfer and retrieval is most often undertaken by dedicated multidisciplinary teams with specialised equipment and additional training [12, 16]. This is not the case in South Africa where these transfers are mostly undertaken by prehospital emergency care providers with no more than their baseline training [17]. Previously, calls have been made to standardise training and core competency standards for all providers that undertake neonatal and Critical Care Retrieval (CCR) [18]. Yet, this field, at least in South Africa, is still quite nascent which necessitates a need to determine its current state and areas for development. To our knowledge, there are currently no courses dedicated to neonatal critical care retrieval in South Africa.

The way that education is conducted in South Africa for pre-hospital emergency care providers has changed in recent times by discontinuing vocational training short courses. All courses are now provided by tertiary institutions and graded on the National Qualifications Framework (NQF). The closure of the vocational course registers has resulted in a downward trend in the output of newly qualified emergency care personnel with a major shortage predicted by 2030 [19]. The current EMS cadre is represented by only 4% ALS providers of which the number of those still active unknown [19]. It is recommended that at least 10% of the EMS cadre should be ALS providers [19], and with an unlikely forecast of improvement, it will further have a negative impact on the ability to conduct neonatal critical care transfers.

The critical care curricula of the degree in emergency medical care programmes in South Africa were compared in a local study [20]. Neonatal critical care retrieval was not a stand-alone course offered, and the content was mixed with other age groups. The curricula also did not mention the specific needs of neonates or how it would be covered. This limited education in neonatal CCR affects the level of care that these patients will receive during retrieval [10].

While previous work have described the perceptions of providers on general paediatric [10] and neonatal [11, 21, 22] interfacility transfer, this has not been done for CCR. CCR refers to the stabilisation and transport of critically ill or injured patients from a healthcare facility where the healthcare requirements of the patient outweigh the diagnostic or treatment abilities to an appropriate facility where these are available [23]. The consensus definition of a Critical Care Retrieval Service (CCRS) include five aspects namely a patient population that meets specific criteria, case selection by specialised call centre personnel, dedicated CCR crew members with additional training and dedicated equipment; and quality management and continuous training [23]. While no criteria have been developed yet, in principle, neonates that require CCR have life-threatening or high dependency conditions, and require intensive monitoring, stabilisation and care. The Critical Care Retrieval Work Group (CCRWG) of South Africa defined that the transfer of neonates that meet CCR criteria should be reserved to dedicated CCR crew members with additional education in this field [23].

Given the limited neonatal education of Advance Life Support (ALS) providers that conduct transfers and the lack of dedicated neonatal CCRS in South Africa, it is the aim of this study to initiate the development of neonatal CCR curriculum which has the potential to reduce AEs.

### Curriculum development framework

To develop additional education in neonatal CCR requires a structured approach. Kern [24] provides a framework of curriculum development in six steps described in Table 1. This study focused on the first three steps of curriculum development, where the final three steps were beyond the scope of this study.

**Table 1:** Kern’s curriculum development [24].

Steps in Curriculum development	Description
Step 1: Problem identification and general needs assessment	The identification of a health need or problem. In this context it is to determine the need for additional education in neonatal critical care retrieval. This includes understanding the current and the ideal approach to education in this field.
Step 2: Targeted needs assessment	Assessing the needs of a specific group of learners, which in this case were ALS providers that conduct neonatal transfers. It also includes understanding their learning environment as it may differ from learners in general.
Step 3: Goals and objectives	The broad objectives of the curriculum is defined which is necessary to evaluate its viability. If resources are limited, objectives can be prioritised to focus on essential areas first.
Step 4: Educational strategies	The specific content is selected and the method required to deliver it effectively to achieve the objectives, is identified.
Step 5: Implementation	Obtaining support and allocating resources to ensure the educational intervention can be implemented. Addressing any barriers and implementing the evaluation process.
Step 6: Evaluation and feedback	Assessing the performance of the program and providing feedback for improvement.

## Methods

This study followed a descriptive qualitative design. Perceptions were explored through semi-structured interviews and a focus group discussion. This study explored the first three steps of curriculum development as described by Kern [24]. In the first step, one-on-one interviews with experts in neonatal CCR were used to conduct the general needs assessment, which clearly defined the problem that helped focus the curriculum’s goals and objectives [24]. The second step of the targeted needs assessment was conducted through a focus group discussion with a group of qualified ALS providers [24]. Information obtained from this group included perceptions of previous learning experiences, perceived deficiencies, learning preferences, barriers and available resources. In step three the identified needs were used to derive course outcomes.

### Research paradigm

This study was conducted within the social constructivist paradigm [25]. The paradigm’s assumptions of relativist ontology are that reality is subjectively based on the understanding of social and experimental levels [26]. This paradigm also includes community of practice as learning does not only originate from a single source or institution, but occurs rather as a result of the community that these providers find themselves in. The community that these providers will learn from are not limited to the prehospital environment either, but include the Emergency Centre (EC) and Intensive Care Unit (ICU) settings as well. Essential knowledge is shared between these individuals and form part of the learning process. It was thus important to include the role-players that form part of this community when developing a new curriculum. Community of practice falls within the theory of situated learning and forms part of a conceptual framework in education [27]. The inclusive process of communities of practice helps with the identification of outcomes during curriculum design as the participation in input by all parties are essential [27].

### Study setting

The study was conducted in the LMIC setting of South Africa where the transfer of neonates are mostly conducted by ALS providers with variable education backgrounds [10] and these ALS providers are of the opinion that they need additional education and specialised equipment to conduct these transfers safely [11]. The participants represented various provinces across South Africa. Both urban and rural areas were represented from the state and private sectors.

### Sampling

#### One on one interviews

The study made use of purposive sampling to recruit participants. The first group of participants were experts in neonatal CCRS and neonatology who were invited to individual one-on-one interviews. The expert group included doctors, nursing professionals and ALS providers working in the field of CCRS or neonatal critical care, with at least five years’ experience.

#### Focus group discussion

The second group of participants were represented by prospective students. The term “student” in this context was any individual that would potentially enrol in the neonatal CCR course in future. Through purposive sampling, prospective students from different genders, representing urban, rural, state and private sectors were recruited. The prospective student group participants were registered with the Health Professions Council of South Africa as paramedics or emergency care practitioners, and actively involved in the transfer of at least one neonate per month.

All participants were recruited through email correspondence. No candidates refused participation, and no participants withdrew from the study. There were no formal relationships established between the researchers and participants prior to study commencement however, some participants were known to the researchers professionally. This was unavoidable given the nascency of CCR in South Africa. The goals of the research and its motivation to develop evidence-informed curricula was made clear to the participants in the informed consent documents and at the start of the interview.

### Data collection instrument

The discussion schedule was informed by literature [17, 20, 28–32], and developed by the authors collaboratively through constant discussion and revision. This schedule was used as a guide by the facilitators during the interviews. The first section of the document was an introduction that gave a background and rationale to the study. The second section gave prompts for obtaining demographic information from the participants with the aim to determine experience and fair representation. The third section of the document provided prompts on exploring the current limitations to education in neonatal CCR within academic and health institutions in South Africa. The fourth section provided prompts on deriving the objectives of a curriculum. The final section focussed on the specific needs to teaching, learning and assessment (TLA) in neonatal CCR education.

### Data collection

The interviews and focus group discussion were conducted online by WS and WW due to the COVID-19 pandemic and the geographic spread of participants. WS has extensive experience in qualitative data collection and guided WW during the interviews, including debriefing sessions. Only participants and the researchers were present during the interviews and focus group discussion. The one-on-one semi-structured interviews with the experts were conducted first, with no repeat interviews required

Participants were provided with a pre-reading document that informed the interviews and focus group discussion. The expert group’s pre-reading document described the neonatal population currently transferred in South Africa, as described by Venter [17]. The diagnoses, attachments and medications involved during the CCR of these neonates by dedicated teams were represented in charts and tables. The document also described the neonatal critical care curricula found during a literature review [20, 28–32].

The prospective student group’s pre-reading document contained the same information, but had the addition of the expert group’s suggested course content and methods of delivery and assessment. The participants were asked to share their opinion on the subject in their private capacities and not those of their employer or organization.

The interviews were guided by formulated questions but strict adherence to these were not required. The interviews and focus group discussion were conducted in English as it will be the medium used in the proposed curriculum. Some new questions arose, and probing was required. The participants were encouraged to speak freely about the topic. Notes were taken during the audio recorded interviews and conducted up to the point where data saturation was achieved. Data saturation was defined as the point where further data collection was counterproductive and new data did not add any more value [33]. The authors felt that data saturation was achieved following the expert group interviews and that no further participants were required.

### Data analysis

All voice recordings were transcribed verbatim by WW, anonymised, and pseudonyms assigned to the transcripts. Data were analysed inductively, using content analysis to the manifest level [33]. The steps of content analysis was based on the methods described by Erlingsson et al. [34] and conducted on nVivo qualitative data analysis software (QSR International, Massachusetts, USA) [35]. The transcripts were read and re-read by WW to get a sense of the content. The text was then divided into condensed meaning units by WW and shared with WS and ET for researcher triangulation. Codes were formulated by WW from the meaning units while maintaining the core meaning. These codes were then grouped into categories and shared with WS, ET and WK for review and refinement. The categories were derived from the data and not predetermined by the authors. For the development of the curriculum map, the codes of topic examples were expanded into course outcomes by WW, and shared with WS, WK and ET for review and refinement. The categorical development is presented in coding tree tables (Table 2, S1 and S2 Appendices in S1 File).

**Table 2:** Categorical development.

Meaning unit	Code	Subcategory	Main Category
*“you would have to understand something about the critically ill infant and where you are taking them”*	Critically ill neonates	Characteristics of neonatal transfers	Current status of neonatal CCR in South Africa
*“I do think there is a difference between rural and urban*.*”*	Urban and Rural variation	Characteristics of transfer teams	Current status of neonatal CCR in South Africa
*“Oh yes*, *I think there’s a need*, *knowing that the medical field evolves …”*	Identifying a need for additional training	Identifying learning needs (Prospective student)	Learning and education in neonatal CCR

### Trustworthiness

The trustworthiness of the research was ensured through credibility, transferability, confirmability, and dependability [36]. Credibility deals with how believable the research findings are. During the interview, the researcher asked the participants if the captured information was interpreted correctly using summarisation and clarification. To achieve transferability and dependability, [37] the researcher and co-authors analysed the data collectively, as multiple points of view balance out the subjective views of the interpreters. Peer debriefing sessions with the co-authors provided different interpretations of the data in an attempt to reduce researcher bias. It was important to ensure that enough contextual information about the study was provided so that the reader can come to the conclusion of transferability [36]. The data were described in such detail that the reader can decide whether it is applicable to their own context. Confirmability was ensured through a transparent and reflexive process of data description and interpretation.

#### Reflexivity statement

The primary author (WW) and the senior author (WS) are emergency care practitioners with extensive experience in neonatal retrieval. Through their involvement in neonatal retrieval and clinical governance, a need was identified to improve education and training in South Africa. WS leads a working group that seeks to develop CCR in South Africa. ET is a research psychologist with extensive methodological expertise, while WK is an emergency physician with expertise in curriculum development.

### Ethical considerations

Ethical approval for the study was obtained from the Human Research Ethics Committee of the University of Cape Town (HREC Ref 474/2020). Written and verbal consent was obtained from all participants prior to data collection.

## Results

### Expert group

The first group of participants enrolled were the expert group, which included six participants with extensive experience (>5 years’) in neonatology and CCRS. The participants’ demographic information is presented in Table 3.

**Table 3:** Demographics of expert group participants in semi-structured interviews.

Expert Group Participant	Self-identified Gender	Qualification	Neonatal CCR Experience (>5 years’)
E1	Male	ECP	Extensive local CCR and education experience
E2	Female	Paediatrician	Extensive international CCR experience
E3	Female	ECP, Nursing Sister	Extensive local CCR and education experience
E4	Female	CCA, Nursing Sister	Extensive local CCR experience
E5	Female	Neonatologist	Extensive in-hospital neonatal experience
E6	Male	CCA	Extensive local CCR experience

ECP: Emergency Care Practitioner

CCA: Critical Care Assistant

CCR: Critical Care Retrieval

### Prospective student group

The second group of participants included seven prospective students (as previously defined) involved in neonatal transfers in South Africa. The participants’ demographic information is presented in Table 4.

**Table 4:** Demographics of prospective student group participants in focus group discussion.

Participant	Self-identified Gender	Qualification	Area of employment	Health sector employment	First Language
L1	Male	CCA	Gauteng	State, Rural	Non-English
L2	Male	CCA	Gauteng	State, Urban	Non-English
L3	Male	ECP	Gauteng	State, Rural	Non-English
L4	Male	ECP	Eastern Cape	State, Urban	Non-English
L5	Male	CCA	Limpopo	State, Rural	Non-English
L6	Female	ECP	Western Cape	Private, Urban	English
L7	Female	ECP	Gauteng	State, Urban	Non-English

CCA: Critical Care Assistant

ECP: Emergency Care Practitioner

The semi-structured expert group interviews had a mean (range) duration of 59 minutes (51–68 min). The semi-structured focus group discussion had a duration of 91 minutes.

The recordings were transcribed, analysed and 372 codes were extracted from the expert interviews. The audio recording from the prospective student group online discussion was transcribed and analysed with 97 codes extracted. No clarity was required from the participants during the transcription phase. The main categories were: Current status of neonatal CCR in South Africa; Learning and education in neonatal CCR; and proposed curriculum elements. The results are summarised in Table 5.

**Table 5:** Main categories, sub-categories and codes.

Main Category	Sub-category	Code	Description
Current status of neonatal CCR in South Africa	Characteristics of neonatal transfers	Transfer case mix differences and similarities	Participants comparing the presented neonatal transfer case mix [17] to their own settings
		Long distance transfers	The distance between dispatching and receiving facilities
		Transfer to tertiary units	CCR transfers are usually an escalation of care
		Time delays	Arrival of team, on scene time and long distances
		Need for improved systems	CCR transfer systems
		Critically ill neonates	The clinical state of neonates for CCR transfers
		High Risk	Risk of adverse events during transfer
	Characteristics of transfer teams	Urban and Rural variation	Team composition differ between urban and rural areas
		The Ideal CCR Team	The ideal CCR team in a resource rich setting
		Variable competency levels	Variation of competency levels between transfer teams
	Limited Resources	Limited backup	Availability of specialists to consult with or specialist teams to come and support
		Limited Dedicated teams	Dedicated CCR teams are limited in SA
		Variable equipment levels	Variation of level of specialised equipment between transfer teams
		Limited Neonatologists	Limited specialists within the Health network
		Limited NICU units	Limited specialist units within the Health network
Learning and education in neonatal CCR	Current education in CCR in South Africa	Need for additional neonatal education	Participants reflect on the need for additional education in neonatal CCR
		Limited neonatal exposure	As students, participants had limited hands on exposure to neonates
		Limited time to cover neonatal content	Not enough time spent on neonates on current education offerings
		Barriers to accessing international education	International neonatal transfer courses are expensive, face to face in another country or reserved for doctors and nurses
		Lack of local context in international offerings	The international courses do not cover all requirements for the local context
	Bridging the knowledge gap (Experts)	Lack of preparedness for neonatal CCR	Participants reflecting on their lack of preparedness for neonatal CCR transfers
		Experience and exposure during neonatal transfer	Gaining experience during transfer of neonates
		Mentorship	Mentorship by experienced and senior individuals
		Self-Study	Reading of journals, text books etc to improve neonatal knowledge
		Clinical shifts in NICU	Spending time in NICU and learning from experts
	Identifying learning needs (Prospective students)	Identifying a need for additional training	Prospective student participants reflect on the need for additional education in neonatal CCR transfers
		Bridging knowledge gaps	How prospective student participants closed their perceived knowledge gaps in neonatal CCR transfers
		Preparedness for neonatal CCR	Prospective student participants’ preparedness for neonatal CCR transfers
		Limited operational exposure	Limited neonatal CCR transfers compared to primary calls
		Curtailment of scope	Change in ALS scope required to improve access to neonatal CCR

### Category 1: Current status of neonatal CCR in South Africa

The participants described the current state of neonatal CCR in South Africa and it outlines current practices, shortcomings, caseload, and volume. It also outlines the importance of neonatal CCR. Three sub-categories were developed: characteristics of neonatal transfers, characteristics of transfer teams and limited resources.

#### Characteristics of neonatal transfers

This sub-category describes the type of patients transferred and under what conditions these transfers took place, including limitations and challenges experienced.

Perceptions of the expert group on the presented case mix from Venter et al. [17] is described first. Experts agreed with the representation in the case mix when compared to their own experiences. However, some experts felt that CHD and prematurity were underrepresented in the description. *“We do see a lot of CHD as well*. *Prematurity and CHD are the bigger ones”* **Expert no. 3 (ECP/nursing sister).** Experts also discussed the variation in case mix between private EMS and the state sector. They felt that neonatal transfers in the state sector was less complex from an equipment and infusion perspective but that the patients were also very sick. *“Perhaps a little bit less cluttered and a little bit less complex*, *maybe very sick*, *but maybe not as complex as this”* **Expert no. 5 (Neonatologist).** They also felt that, reflecting on the presented case mix, it was important to have a better understanding of what types of cases are being transferred in both the state and private sector when informing studies. *“But I do feel it’d be important to see what type of cases they would see in state as well”* **Expert no. 1 (ECP).**

Prospective students explained that one of the challenges in neonatal CCR are long distance transfers. *“Then we have a tertiary hospital that is about 197 kilometres away from the base that I work from”* **Student no. 5 (CCA)** and that the distance does come with risk of adverse events occurring. *“…adverse events during these long-distance transfers”* **Student no. 2 (CCA).**

Another challenge with CCR described was the effect that time delays had on the patients. “*Because of the time delays*, *very often that child was much sicker than they probably would have been if they had been able to get there much quicker*” **Expert no. 5 (Neonatologist).**

The conditions under which these transfers occur were further elaborated on when experts explained that the teams also have to transfer neonates *“across the province*, *and from various outlying facilities*” and it was also suggested that there needs to be a *“better system in place to say*, *to prevent adverse events in neonates*” **Expert no. 1 (ECP).**

The neonates transferred in South Africa were also described by the experts as “*really sick intubated*, *ventilated neonates*” **Expert no. 1 (ECP)** and needed to be transferred based on their acuity. The experts also described the current state of neonatal transfers as high risk in nature. *“Because*, *these transfers are high risk*, *and these neonates are very sick”* **Expert no. 5 (Neonatologist).**

#### Characteristics of transfer teams

This sub-category describes the qualification and composition of current team members who conduct neonatal transfers and the ideal team composition. The experts felt that there was a difference between the qualifications of the ALS providers that transfer neonates in rural and urban areas. *“I do think there is a difference between rural and urban*. *Where in Johannesburg [major urban city] it is ECPs and in Welkom [small rural town] it is a CCA*. *And in Kathu [small rural town] it is a CCA*. *That is why I think the reality is we cannot exclude those people (non-degreed providers)* [in training initiatives]*”* **Expert no. 3 (ECP/nursing sister).**

The ideal CCR team/system was described by experts. This included team composition (Dedicated/Multidisciplinary) and specialised equipment *“…the international standard is that transfers or transport of neonates*, *especially the sick ones should actually be a multidisciplinary team event”* **Expert no. 5 (Neonatologist).**

Dedicated CCR teams were suggested to ideally transfer neonates. “*I know we’re very fortunate with our company that*, *you know*, *we’ve pretty much got an understanding here that anything that smells*, *sounds or looks like a neonate we’ll be going with the critical care retrieval team”* and the service was also described as *“*..*in order to make it safe for the practitioner and safe for the client (patient)*, *I think it needs to be a speciality”* **Expert no. 4 (CCA/Nursing sister).** A multidisciplinary team approach was described as the ideal “*because if you’ve got those different disciplinaries with different scope of practice…*..*and do the transfer to the best of the ability of the team”* **Expert no. 6 (CCA).**

The experts described the documentation that the teams should have during CCR as currently limited “*shortcomings was documentation”* and that from “*medico-legal perspective*, *this is a concern”*. Extensive documentation was described as a necessity “*to communicate effectively to the other side* [receiving centre]*”* **Expert no. 2 (Paediatrician).**

There was also “*a variation in confidence”* described where some current ALS providers were found to be “*nervous about handing over to the ICU doctor*” and then others that had “*high level of confidence amongst the staff picking up babies for transport”* **Expert no. 2 (Paediatrician).**

#### Limited resources

This sub-category describes the limited in-hospital specialist services available creating the need for transfer and the limited resources available to transfer high acuity neonates.

Experts felt that there is *“limited sort of backup”* and that ALS providers who conduct the transfers “*have to have backup”* **Expert no. 2 (Paediatrician).** The experts went on to describe dedicated CCR teams as a limited resource. “*What we understand is that the advanced teams are very few”* **Expert no. 5 (Neonatologist).** Prospective students also described dedicated CCR teams as limited. *“…in the Western Cape*, *we have a (single) dedicated neonatal transfer unit”* **Student no. 6 (ECP).**

Equipment failure as a limitation was described as “*sometimes they (transfer team) were also let down to a certain extent by the quality of the equipment they had*” **Expert no. 5 (Neonatologist).**

Limited specialists available to care for these patients was also described as a challenge and reason for transfer. “*Retrieving babies from outlying areas*, *bringing them back into level one hospitals and to the neonatologists*” **Expert no. 4 (CCA/Nursing sister).** Experts also described that there “*aren’t that many neonatal intensive care units*” in the rural areas and that “*they’re only really in the major cities*” **Expert no. 2 (Paediatrician).** Prospective students also described NICUs as a scarce resource. *“Here in Limpopo we have about seven hospitals*, *of which six of those are primary*. *And one is secondary*. *There’s the one with about two to three neonatal ICU beds (for the province)”* **Student no. 5 (CCA).**

### Category 2: Learning and education in neonatal CCR

This category describes the current state of education (including barriers to access) in neonatal CCR and how any identified educational gaps were bridged. The category was divided into the following sub-categories: current education in CCR in South Africa; bridging the knowledge gap (experts); and identifying learning needs (prospective students).

#### Current education in neonatal CCR in South Africa

This sub-category describes why there is a need for additional education in neonatal CCR and barriers to accessing further education in the field.

Experts attributed the need for additional education to limited exposure to neonates in the current education system and was described as “*I think that is maybe why the basics is so lacking because they don’t see neonates…*. *you see them but you see them from a distance”* **Expert no. 3 (ECP/nursing sister).**

The time spent on neonatal education was described as limited. “*ideally yes*, *if we can edit the time spent on neonatal content we could get more value out of it*.*”* and that “*I don’t think that they can be adequately prepared in the time frame”* **Expert no. 3 (ECP/nursing sister).**

Experts expressed some barriers to accessing international neonatal CCR education. The international courses were firstly described as limited *“*..*quite limited programs out there”* and cost-prohibitive “*the (high) cost associated with them as well”*. Some of these courses were also described as *“*.. *reserved for physicians and not so much for paramedics”* **Expert no. 3 (ECP/nursing sister).**

The prospective student group discussed the availability of resources to access additional neonatal CCR education. Prospective students felt that electronic devices and internet access to participate in online teaching was not such a barrier anymore. *“People*, *kids in the rural areas have tablets*, *we have cell phones*, *like I’m currently using my cell phone”* **Student no. 7 (ECP).**

From a cost perspective, prospective students felt that employer buy-in and funding was very important. *“I think it’s imperative for the employer to support this kind of program*, *because it’s going to be beneficial for both practitioners and their employer”* **Student no. 2 (CCA).** Prospective students also felt that paying for the course themselves would be possible for some. *“But if it’s something that you personally feel like you will be doing*, *you can also try and pay for yourself”* **Student no. 7 (ECP).**

The prospective student group felt that this kind of education should be inclusive for all ALS providers and that it should change the current scope of practice limitations. *“So*, *I think a course like this*, *if then allowed afterwards for the CCA and the short courses to have access to*, *those qualifications where they can now do these transfers would be such a huge help”* **Student no. 6 (ECP).** It was also suggested to be a specialty for qualified pre-hospital providers. *“It should be as a standalone course for a specialised team”* **Student no. 5 (CCA).**

The experts suggested that some sections of the international curricula presented in the pre-reading document could “*literally take the courses that you’ve outlined here*, *and pull pieces out and put them all in to make a course”* but that “*we can’t just take one of those courses and use it as is in our local context”* **Expert no. 4 (CCA/Nursing sister)** as they lack local context specific content.

#### Bridging the learning gap (experts)

This sub-category describes the expert group’s preparedness and perceived learning gaps and how they overcame them.

The experts described the current situation of education in neonatal CCR in South Africa. They described that *“very little of the training that we actually did*, *prepared me for that first neonatal transfer”* and that there is a “*need for new graduates to get more training in neonatology*.*”* The current programmes were also described as *“not designed in such a way that you have a critical care retrieval specialist”* **Expert no. 1 (ECP).**

The expert participants shared their experience of how they bridged the learning gap when they started conducting neonatal CCR. Exposure to neonates in transfer was described as “…*the more you do it (neonatal CCR)*, *the more you get comfortable…”* They mentioned that mentorship played an important role too. “…*what was very nice is that at the state hospital there was quite a great doctor*, *that every time we went there with a patient*, *we could get some knowledge from her and especially on things that we did not know”* **Expert no. 6 (CCA).**

Experts also described self-study as a way of bridging the learning gap. “..*then obviously some literature searching from a lot of the American stuff helped fill some of those gaps*”. Spending time in a NICU was also described as a way to learn more about neonates “*go to the unit and started discussions… would like to understand more from NICU*” **Expert no. 1 (ECP).**

#### Identifying learning needs (prospective students)

This sub-category explored the student’s need for additional education in neonatal CCR through discussions on perceived knowledge gaps, preparedness, exposure and limitations in scope of practice.

The prospective student group agreed with the need for additional education in neonatal CCR. *“Most definitely*. *Let me tell you about my experience*. *I remember doing my first neonatal ICU transfer*. *It was really nerve wracking because now you don’t have anyone to rely on*. *If you could get a course and do it part time”* **Student no. 3 (ECP)** *and “Oh yes*, *I think there’s a need*, *knowing that the medical field evolves …so there’s definitely a need to always get more knowledge”* **Student no. 1 (CCA).**

Prospective students also shared how they closed their perceived neonatal CCR knowledge gaps. Prospective students expressed how they became more comfortable with managing neonates following exposure during actual transfers on the job. “*But you learn every day*, *when it comes to how to handle the patient you learn every day because you handle a patient in a certain way*, *when you’re transferring a patient*, *when it comes to the tube securing or the other attachments with a patient”* **Student no. 7 (ECP).** They also felt that literature searches helped close the knowledge gap. *“…go into the internet and read about that patient and what they had and how to manage them better in future”* **Student no. 7 (ECP).**

Prospective students were asked to share their opinion on their preparedness for neonatal CCR when they were newly qualified. They felt that they were not prepared for these transfers. *“But we didn’t cover much of how to manage a neonate in the ICU transfers”* **Student no. 2 (CCA).** They felt like they were better prepared on a basic level for neonatal transfers. *“I feel like the university did pull through in like preparing me on the basics that I need to know about transferring a neonate or treating a neonate patient”* **Student no. 7 (ECP).**

They also reflected on the time that was dedicated to neonatal CCR on their respective courses. Prospective students with the university bachelor’s qualifications shared that neonatal content was mainly covered in fourth year and that time was limited. *“You only really deal with neonates in fourth year towards the second half of the year*, *where we did intensive neonatal ICU training*, *including medication dosages*, *pathology*, *ICU transfers*, *etc*.*”* and “*Because even within the BEMC (program) things felt rushed*, *there were definitely topics that we could have gone in more depth or spend more time with”* **Student no. 6 (ECP).** The prospective students with the CCA qualification background shared a similar experience where they felt not enough time was allocated to neonatal content. *“The CCA program was about nine months*. *Now we have to cover the literature and the practicals within that nine months*. *Now we didn’t spend much of the time in terms of paediatrics and neonatology”* **Student no. 2 (CCA).**

Prospective students shared some of the content that they were taught on their programs as it relates to neonatal CCR. *“…regarding neonates*, *the way the curriculum works is that during first and second year you do anatomy and physiology”* **Student no. 6 (ECP).** Neonates and the rest of the paediatric group were combined during lectures on the bachelor’s program and only separated by individual drug dosages. *“I’d say basically*, *because they didn’t actually separate it*. *It was paediatrics and neonates*, *but then obviously*, *the doses and how you treat them”* **Student no. 7 (ECP).**

The prospective student group felt that experiential learning on their programs were beneficial but had some limitations. *“And a lot of the learning that took place was also reliant on what you learned during your experiential shifts”* **Student no. 6 (ECP).** One limitation was exposure to neonates in a CCR environment. “*But because it’s such a specialist service*, *there were not that many neonatal transfers going around as a student to get exposure*.*”* **Student no. 1 (CCA).** Prospective students felt that more hands-on experience was gained during NICU shifts. *“Whereas I got more experienced learning in the neonatal ICU shifts where the sisters were really kind and gave you a baby for a week to look after and you would feed the baby*, *clean the baby*, *do all those things with the nurses”* **Student no. 6 (ECP).** In addition to exposure as a student, prospective students also explained that operational ALS providers have limited exposure to neonates. *“…working on the road*, *see minimal neonatal transfers in the private sector*, *just because of medico-legal risks*, *and they have a specialised unit”* **Student no. 6 (ECP).**

### Proposed course structure and outcomes

Table 6 describes the curriculum structure as proposed by the expert and prospective student groups. Further, broad course outcomes derived from the interview content is proposed. The derivation process is shown in the coding trees contained in the supplementary attachments (S1 and S2 Appendices in S1 File).

**Table 6:** Proposed curriculum structure.

Proposed Curriculum Structure			
Course Duration	• Part time post-graduate diploma (Proposed by prospective student and expert group) or • Part time master’s degree (Proposed by expert group)		
Course Model	• Blended course–Online/Workplace/Simulation-Based Teaching, Learning & Assessments • Mentorship programme		
Resources	• Electronic Devices • Specialist supervision • Cost: self/employer funded		
Targeted Student Group	• The course should be inclusive for all Advanced Life Support providers.		
Suggested scope of the course	• Scope of practice should change after course • The course should be considered as an elective speciality for ALS providers in South Africa		
• Developing Curriculum Map			
Course Outcomes	Proposed Assessment Methods		
	Formative Assessments	Continuous Summative Assessments	Summative Assessment
Describe anatomy and physiology as it relates to the neonate^1^	Work-Based Assessments Simulation-Based Assessments Written Assessments	Work-Based Assessments Simulation-Based Assessments Written Assessments	Portfolio of Evidence
Describe the components of an effective critical care retrieval service. ^2^			
Apply transport considerations and the effect of transport stress on a range of conditions specific to the critical neonate. ^3^			
Explain the continuity and maintenance of neonatal critical care and its application during retrieval and transport. ^4^			
Describe and prepare the documentation required for safe transitions of care during the handover of a neonatal patient. ^5^			
Demonstrate competence in the application and performance of emergency procedures for neonatal resuscitation and stabilisation. ^6^			
Demonstrate the safe use of equipment utilised for the monitoring and management of the neonate during transport, heating, mechanical ventilation and infusion devices. ^7^			
Describe and demonstrate the monitoring, care, management and troubleshooting of a variety of indwelling attachments as they relate to neonatal critical care and transport, including drains and different methods of vascular access. ^8^			
Describe and demonstrate the monitoring, care and management of a variety of medications as they relate to neonatal critical care and transport. ^9^			
Demonstrate the initial and ongoing assessment of a neonate as it relates to neonatal critical care and transport. ^10^			
Apply and analyse a variety of methods used for the monitoring of a critical neonate during retrieval and transport. ^11^			
Describe the physiological effects that the different modes of transport could have on the neonate and apply methods to mitigate potential risks to the neonatal patient during transport. ^12^			
Describe and demonstrate the monitoring, care and management of a variety of ventilation modes and methods as they relate to neonatal critical care and transport. ^13^			

Topic examples provided by participants:

1) Neonatal anatomy and physiology

2) Critical Care retrieval systems

3) Specific conditions suggested are: Conditions from the study [17]; congenital heart defects; infection; prematurity; respiratory conditions (bronchopneumonia, diaphragmatic hernia, meconium aspiration, persistent pulmonary hypertension); surgical emergencies (gastroschisis, necrotising enterocolitis)

4) Examples include feeding and skin care

5) Specific to referral documents, information gathering, and handover

6) Examples include: airway management, neonatal resuscitation, thoracentesis

7) Examples include: incubators, infusion devices, mechanical ventilators (see no. 13)

8) Examples include: Colostomy bags, drains, vascular access (arterial access, central intravenous access, intraosseous access, peripheral intravenous access, umbilical vascular access)

9) No specific examples given

10) Assessment of the neonate, and specifically for the conditions in 3.

11) Examples include: arterial blood gas, electrocardiogram, end-tidal carbon dioxide monitoring, fluid balance, glucose management, perfusion, thermoregulation.

12) Examples include: acceleration and deceleration forces and effects, mode of transport and their stressors, motion and movement, noise, patient packaging.

13) Examples include: manual bag valve mask resuscitators, device neonatal resuscitator (e.g. NeoPuff), oxygen blending, humidification, heating of air and circuits, continuous positive airway pressure, mechanical ventilator modes, oscillation and oscillation takeover.

## Discussion

This study aimed to derive course outcomes in neonatal critical care retrieval education by conducting a general and targeted needs assessment. First, a group of experts in neonatology and neonatal CCR took part in semi-structured one on one online interviews. Next, a group of prospective students participated in an online focus group discussion. The first main category identified during content analysis was: Current status of neonatal CCR in South Africa, which described the characteristics of neonatal transfers, the teams that conduct them and the limited resources in the South African setting. The second main category was learning and education in neonatal CCR, which described current education offerings, how participants bridged their perceived knowledge gaps and identifying learning needs. Finally, proposed curriculum elements and course outcomes were derived.

Neonatal CCR were described as often taking place over long distances, which was also reported in a single centre retrospective study by Royal et al. [14] in the Kwazulu-Natal province of South Africa. The centralisation of specialist care in a LMIC such as South Africa is unavoidable, which requires a strengthening of the CCRS network with dedicated multidisciplinary teams. Time delays were reported as a challenge, which was one of the contributing factors to adverse events in a single centre prospective study by Senthilkumar et al. [9] in the United Kingdom. Neonatal CCR transfers were also described as high risk in nature. This result was echoed in a prospective observational study by Goldsmit et al. [4] in Argentina, and in a prospective descriptive study by Henry et al. [5] in Jamaica, where the clinical deterioration of neonates during transfer was described. The ideal CCR team was described as a dedicated multidisciplinary team, also reported by Ramnarayan et al. [12] in the United Kingdom, where patients transferred by these teams had a better outcome than patients transferred by non-specialist teams. Limited dedicated teams that can conduct neonatal transfers safely was described, a finding also reported by Dalal et al. [7] in India where only half of neonates that undergo inter-facility transfer arrived by ambulance, leading to high adverse event rates. Interestingly, the lack of neonatal transport is one of the top ten preventable causes of under 5 year mortality in South Africa [1]. In a cross sectional study by Sogomo et al. [6] in Kenya, a lack NICUs and specialists created the need for neonatal transfers, which was also reported by the participants, especially in rural settings. Given the long transport times and expansive distances, providers who undertake neonatal CCR in these contexts should be equipped and knowledgeable to care for neonates for prolonged periods; thus strengthening the case for the development of additional, bespoke educational offerings.

Current education in neonatal CCR in South Africa was described as limited due to time constraints to cover content. This finding was similarly demonstrated by Conradie et al. [20] where the CCR curricula offered on the South African emergency medical care degree programmes were compared. The study found that neonatal CCR was not offered as a standalone module, but rather covered with other age groups. Experts in neonatology and neonatal CCR described perceived knowledge gaps, which they had to overcome to conduct neonatal CCR safely. Similarly, in a study by Senthilkumar et al. [9] adverse events were linked to the level of education of the transferring team. The need for additional education in neonatal CCR was described by the prospective student group. This result was also reported by Ismail et al. [11] in a descriptive study where ALS providers from the Western Cape, South Africa felt that they require additional exposure and training in neonatal transfers. The same result was also reported by Ashokcoomar et al. [22] in a descriptive study where seven neonatologists from the state sector in South Africa indicated that ALS providers who transfer neonates require specialised education. In another descriptive study by Ashokcoomar et al. [21] 43 ALS providers and 21 Emergency Medical Care Lecturers from South Africa described the educational preparedness of ALS providers to conduct neonatal transfers as limited and that deeper more specialised training is required.

The need for adequately trained personnel to safely transfer high acuity paediatric patients in South Africa was described in a qualitative study by Vincent-Lambert et al. [10]. In addition to this, the CCR working group of the Emergency Care Society of South Africa highlighted the importance of dedicated CCR crew members with additional education in this field, to undertake neonatal CCR [23]. The limitations in current offerings of South African neonatal CCR education and the desperate need described by participants of this study warrants the urgent development of additional training in this field. To this end, we report a proposed curriculum structure with course outcomes as a solution towards improving neonatal CCR in a LMIC setting.

A post-graduate diploma was suggested as the most favourable exit level for the proposed education programme. A master’s degree exit level was also reported as an option, however this may be a barrier to access for non-degree ALS paramedics. Such a programme, as an elective speciality, might allow for an increased scope of practice of prospective students as a standard in neonatal CCR. A model of blended learning was suggested which included online, workplace and simulation-based teaching, learning and assessment with a robust mentorship programme. In a prospective study by Tabas et al. [38] a blended approach including online, face-to-face and practical hands-on components were successfully employed to teach advanced emergency procedures to medical students in South Africa. The practical component suggested by participants is in line with the theory of situated learning where students will learn within their community of practice. The benefit of an online component was also demonstrated by Stassen et al. [39] where the implementation of an online course in managing traumatic brain injury (TBI) patients improved the care provided by Helicopter Emergency Medical Services (HEMS) personnel. The COVID-19 pandemic accelerated the use of online learning as a teaching medium, which is controversial in an LMIC setting with a digital divide due to an unequal society. Participants reported that most prospective students (employed professionals) have access to online learning resources. In South Africa, 70% of the population are internet users [40], and it is almost certainly higher in professional populations with a stable income. Mpungose [41] suggests the inclusion of free social media platforms as part of online learning offerings to make information more accessible to students with financial barriers.

The participants suggested topics that they felt should be covered on the proposed course and suggested that these are essential areas of knowledge and skills needed to manage an ill neonate during transfer. The transfer of neonates is not limited to road ambulances, as some specialised services also make use of helicopter and fixed wing ambulances. These modes of transport could have negative physiological effects on the neonate and participants felt that it was important to understand how to mitigate potential risks and ensure safe continuity of care to the receiving facility. It is important to note that these topics were informed by actual patients from the study by Venter et al. [17]. The importance of informing a South African curriculum with a local case mix was demonstrated by Cohen et al. [42] in a cross sectional retrospective audit study. The participants provided topics based on real life experiences and actual patients representing various provinces across state and private sectors.

It is encouraging that some neonates are being transferred by dedicated CCRS teams in South Africa. Unfortunately, this service is very limited in LMIC settings, especially in rural areas where access is mostly reserved for patients that can afford private health care. The only way to reduce adverse events during neonatal transfers is to invest in developing CCRS systems. Policymakers have to agree that neonates that meet specific criteria, irrespective of socio-economic status, have to be transferred by specialised CCRS teams.

Once course content has been developed through consensus and wider engagement, it is essential that additional education in neonatal CCR should become an adopted standard qualification for all CCRS team members. Importantly though, education in neonatal CCRS should be widely accessible to all providers that will transfer neonates, whether complex critical care cases or not. In this manner, successful implementation of such an educational programme has the potential to improve patient safety, minimise adverse events during transfer, and impact under-five mortality favourably.

The focus of this study was on the South African setting where interfacility transfer is a function of EMS and neonatal CCR is undertaken by ALS providers. This curriculum could likely be adopted as is, or adapted for other professional groups like nursing or physicians who often undertake neonatal CCR in other contexts. Future research should determine to what extent this might be suitable for those professions.

### Limitations

This study had some important limitations. The experts in neonatal critical retrieval that participated were representative of CCRS in South Africa, however it will be necessary to further consult with the different stakeholders and then undertake a consensus process in order to finalise the curriculum and content. During these phases, a broader and more systematic consultation with neonatology and other expert groups may be necessary. The prospective student group of participants were representative of the current South African ALS providers involved in neonatal transfers, but a larger more diverse sample would benefit the curriculum design process. This study only focussed on the initial steps of curriculum design, but the development of curriculum content and implementation models do create an opportunity for future research. Once developed, implementation of the course should be evaluated in terms of knowledge gained and retained, confidence and clinical impact.

## Conclusion

This study described the need for additional education in neonatal critical care retrieval due to the limitations in the current and past education systems. This study provides a curriculum structure with course outcomes that can be used as a basis for the development of a complete curriculum for education in neonatal CCR, with the potential to greatly reduce adverse event rates.

## Supporting information

S1 File (DOCX)

S1 Data (ZIP)
