# Supplementary material for: Developing a South African curriculum for education in neonatal critical care retrieval: An initial exploration
Source: PLoS One. 2023 Aug 31;18(8):e0290972. doi: 10.1371/journal.pone.0290972 (PMC10470938; doi:10.1371/journal.pone.0290972)
Supplement: S1 File — (DOCX) [file pone.0290972.s001.docx]

## Appendices

**Appendix S1: Coding tree for course outcomes**

| **Course Outcome** | **Topic** | **Code** | **Participant Quote** |
| --- | --- | --- | --- |
| Describe anatomy and physiology as it relates to the neonate | Neonatal anatomy and physiology | Anatomy and physiology  Neonatal specific | The participants shared their opinions on the learning objectives that should be included in the proposed course. Neonatal specific *“anatomy and physiology were to be covered”* and a *“good understanding of the general pathophysiology that we would find in neonates”* **Expert no. 1 (ECP).** |
| Describe the components of an effective critical care retrieval service. | Critical Care retrieval systems | CCR Systems   \| Adverse events \| \| --- \| \| Appropriate receiving facility \| \| Escalation pathways \| \| Network limitations \| \| Patient criteria for transfer \| | The participants mentioned that there should be a better understanding of CCR systems. “…*from the time the call comes in, and how the call gets screened, and how the call then gets dispatched to which vehicles and so on”* **Expert no. 6 (CCA).** Under CCR systems, they mentioned that students must understand “*what adverse events are…and quality assurance and patient safety”* **Expert no. 1 (ECP).** Student participants also felt that quality assurance was lacking in the EMS industry and that it should be included in the proposed curriculum but should implemented across the industry to affect change. *“Definitely, I do think so. But if quality assurance isn't existing with any patient at all, I don't think you know, just trying to implement that for the neonate side will be effective”* **Student no. 4 (ECP).** The importance of choosing an appropriate receiving facility was described as *“you would have to understand something about the critically ill infant and where you are taking them”* **Expert no. 5 (Neonatologist).** The understanding of escalation pathways was also highlighted as you need to *“consult with maybe the receiving specialist to discuss…”* and *“know when and who to call for help”* **Expert no. 2 (Paediatrician).** The network limitations were also discussed where the transferring professional has to understand the *“limitations and the physical sort of directions of care within the networks”.* There also needs to be an understanding of the patient criteria for transfer and that some patients are *“not stable enough to be moved…the outlook is that they are not going to survive”* **Expert no. 2 (Paediatrician).** |
| Apply transport considerations and the effect of transport stress on a range of conditions specific to the critical neonate. | Specific conditions suggested are: Conditions from the study ([1](#_ENREF_1)); congenital heart defects; infection; prematurity; respiratory conditions (bronchopneumonia, diaphragmatic hernia, meconium aspiration, persistent pulmonary hypertension); surgical emergencies (gastroschisis, necrotising enterocolitis) | Conditions   \| Conditions from study ([1](#_ENREF_1)) \| \| --- \| \| Congenital heart defects \| \| Infection \| \| Prematurity \| \| Respiratory:  Bronchopneumonia  Diaphragmatic hernia  Meconium aspiration  Persistent pulmonary  hypertension  Surgical emergencies  Gastroschisis  NEC \| | The participants described the neonatal specific conditions that will have to be covered during the proposed education. Participants suggested that the conditions described by M. Venter (2021) ([1](#_ENREF_1)) should be included. “*The curriculum should be weighted on those specific studies and the cases that we’ve seen (in the study)”* **Expert no. 6 (CCA).** Specific conditions were described as *“…understanding of congenital (cardiac) abnormalities”* was mentioned by **Expert no. 1 (ECP)** and that *“I know that with cardiac conditions it can be a bit tricky”* **Expert no. 5 (Neonatologist).** The student group participants shared their opinion on some specific conditions that need to be covered in the proposed curriculum. *“I think it is very important, what can be included in this is the various birth defects that neonates may have. Whether common or rare”* **Student no. 3 (ECP).** Participants added other conditions that are not that common. *“…when you start working with this little ones you start to experience things that you will never experience in class and all the practical stages. I worked for 10 years in Gauteng and never came across a case of gastroschisis”* **Student no. 5 (CCA).** Infections as a condition in the neonate was mentioned as *“sepsis and how to manage these patients”* **Expert no. 1 (ECP).** Participants added to the conditions with “*prematurity, I would put as its own medical emergency or disease category”* Student participants also shared that how to “*prevent contamination, infection, all those things”* **Student no. 5 (CCA)** should be included**.** The inclusion of trauma in neonates was also shared as an outcome. *“Trauma was not something that was deeply dealt with when it comes to these small little babies”* **Student no. 7 (ECP).** Participants further elaborated on specific conditions under respiratory. It was described that *“a practitioner should know more about ….bronchopneumonia of these patients”* **Expert no. 6 (CCA).** *“Diaphragmatic hernia”,* “*meconium aspiration”* and *“persistent pulmonary hypertension”* **Expert no. 5 (Neonatologist)** was added to the respiratory conditions. Surgical emergencies were described as a condition but more specifically *“gastroschisis…but I think surgical emergencies would probably be a little stand alone”* **Expert no. 5 (Neonatologist)** and *“…your open abdominal things like NEC (necrotising enterocolitis)”* **Expert no. 3 (ECP/Nursing sister).** |
| Explain the continuity and maintenance of neonatal critical care and its application during retrieval and transport. | Examples include feeding and skin care | Continuity of care  Feeding  Skincare | Participants felt that there needs to be an understanding of *“feeding of premature neonates”* and *“aspects of transfer like skin care”* as a continuity of care from the NICU **Expert no. 1 (ECP).** |
| Describe and prepare the documentation required for safe transitions of care during the handover of a neonatal patient. | Specific to referral documents, information gathering, and handover | Documentation  Referral and handover specific | Participants also felt that the necessary *“documentation and the handover communication”* **Expert no. 2 (Paediatrician)** needs to be included in the course. |
| Demonstrate competence in the application and performance of emergency procedures for neonatal resuscitation and stabilisation. | Examples include: airway management, neonatal resuscitation, thoracentesis | Emergency Procedures  Airway management  Chest decompression  Resuscitate | Participants felt that emergency procedures have to be taught on the course. Airway management was described as *“Managing airways …placement of an ET tube”* and chest decompression was also added as *“needle decompression”* **Expert no. 2 (Paediatrician).** Resuscitation of a neonate was described as important to include under procedures as *“..doing a full neonatal resus(citation) from the beginning through straight to the end”* **Expert no. 1 (ECP).** Student participants also felt that *“training with regards to neonatal resuscitation”* **Student no. 6 (ECP)** should be included as an emergency procedure. |
| Demonstrate the safe use of equipment utilised for the monitoring and management of the neonate during transport, heating, mechanical ventilation and infusion devices. | Examples include: incubators, infusion devices, mechanical ventilators (see ventilation) | Equipment  Incubator  Infusion devices  Troubleshooting | Participants further added that neonatal specific equipment will also have to be covered during this course. *“The handling of the incubator ….how to manage it”* **Expert no. 1 (ECP)** was described under incubators. Equipment was further elaborated on by the addition of infusion devices. *“The real, proper use of infusion devices…is quite important because its not just a matter of putting in a syringe and pushing start”* **Expert no. 1 (ECP).** Students will also have to be taught how to troubleshoot equipment as *“tips and tricks, because you’re not always going to have the same equipment”* **Expert no 4 (CCA/Nursing sister).** The student group added to the equipment with humidification and charging stations to the expert group list provided. Specialised equipment described as*“…looking at your equipment as well, you have very limited equipment with treating trauma patients that are that tiny”* **Student no. 7 (ECP).** |
| Describe and demonstrate the monitoring, care, management and troubleshooting of a variety of indwelling attachments as they relate to neonatal critical care and transport, including drains and different methods of vascular access. | Examples include: Colostomy bags, drains, vascular access (arterial access, central intravenous access, intraosseous access, peripheral intravenous access, umbilical vascular access) | Vascular access  Arterial Lines  Central Line  IO access  IV peripheral  Umbilical  Indwelling attachment  Colostomy bags | Participants described vascular access as an important inclusion. Various vascular access methods were described as *“Then line management, select your central lines and you’re a-lines (arterial), reading of that and safety and so forth”* **Expert no. 1 (ECP).** Participants further felt that intra osseous access should also be included*. “…just the whole discussion about intra osseous infusions ….that should be reserved for emergency cases”* **Expert no. 5 (Neonatologist).** Peripheral IV access was also emphasized as *“emergency practice skills such as bag valve mask, ventilation, and placement of an ET tube and then IV access…”* **Expert no. 2 (Paediatrician).** Umbilical vein catheterization was also an important vascular access point mentioned*. “Usually the neonate, you have the advantage of an umbilical cord. So you get into the umbilical vein if you need to use as an emergency”* **Expert no. 5 Neonatologist.** Participants discussed indwelling attachments as an important inclusion with “*colostomy bags”* **Expert no.2 (Paediatrician)** mentioned as a specific attachment on neonates. |
| Describe and demonstrate the monitoring, care and management of a variety of medications as they relate to neonatal critical care and transport. | No specific examples given | Medication  Neonatal CCR specific | Participants felt that neonatal specific medications have to be included. *“And you know, a lot of medications that they (in hospital) use, we (ALS paramedics) don’t have those type of medication on our scope”* and “*when you get to the hospital, you should know what is the adverse effect”* **Expert no. 6 (CCA).** |
| Demonstrate the initial and ongoing assessment of a neonate as it relates to neonatal critical care and transport. | Assessment of the neonate, and specifically for the conditions in 3. | Patient assessment  Neonatal assessment | Participants described that the assessment and monitoring of a neonate is important. “*I would say basic neonatal assessment…what is normal for a neonate?”* **Expert no.3 (ECP/Nursing sister).** Patient assessment was further described as an important inclusion *“…the whole assessment process and the treatment and management of disease processes”* **Student no. 5 (CCA).** |
| Apply and analyse a variety of methods used for the monitoring of a critical neonate during retrieval and transport. | Examples include: arterial blood gas, electrocardiogram, end-tidal carbon dioxide monitoring, fluid balance, glucose management, perfusion, thermoregulation | Patient monitoring  ABG  ECG  ETCO2  Fluid balance  Glucose management  Perfusion  Thermal regulation | Under patient monitoring, more specific considerations were highlighted. Arterial blood gas monitoring was described as *“we would like to see the ABG, especially when we do the transfers that’s very long”* **Expert no.6 (CCA).** ECG monitoring was also mentioned by participants. *“12 lead ECGs on the neonate? I mean, we don’t do those on the road”* **Expert no. 4 (CCA/Nursing sister).** ETCO2 was also specifically mentioned under monitoring. *“…what we are doing is we’ve got your capnography for the little ones”* **Expert no.6 (CCA).** Monitoring of the neonate’s fluid balance was mentioned as “*emphasis on fluid balance, because that’s one of the main things”* and under glucose management *“…is metabolic stability, particularly something like managing hypoglycaemia”* **Expert no. 5 (Neonatologist).** The neonate’s perfusion and thermal regulation was described as *“…absolutely key…how do I manage this baby’s perfusion”* and *“thermal regulation is so important…admission temperature is more predictive of their mortality”* **Expert no.2 (Paediatrician).** The student group of participants added to patient monitoring. They mentioned that fluid balance and thermal regulation are important parameters to monitor and should be included in the course. *“…make sure that the fluids balanced”* **Student no. 5 (CCA)** and *“…warming (kangaroo care etc.)”* **Expert no. 6 (ECP).** |
| Describe the physiological effects that the different modes of transport could have on the neonate and apply methods to mitigate potential risks to the neonatal patient during transport. | Examples include: acceleration and deceleration forces and effects, mode of transport and their stressors, motion and movement, noise, patient packaging. | Transport considerations  Acceleration deceleration  Modes  Movement and sound  Patient packaging | Various transport considerations were described by participants as important inclusions. One consideration was the *“effects of acceleration and deceleration during transport”* and the “movement and sound considerations” **Expert no. 2 (Paediatrician)** on the neonate. Careful consideration of the mode of transfer as *“appraisal of transfers, whether you should be air or road”* **Expert no.1 (ECP)** was also included. The packaging of the neonate in the incubator was described as *“…if we consider how are we going to package this patient so that the patient is safe”* **Expert no. 6 (CCA)** |
| Describe and demonstrate the monitoring, care and management of a variety of ventilation modes and methods as they relate to neonatal critical care and transport. | Examples include: manual bag valve mask resuscitators, device neonatal resuscitator (e.g. NeoPuff), oxygen blending, humidification, heating of air and circuits, continuous positive airway pressure, mechanical ventilator modes, oscillation and oscillation takeover. | Ventilation  Bag valve mask  CPAP  Heated circuits  Humidification  Neopuff  O2 Blending  Oscillation takeover  Ventilators | Participants discussed ventilation considerations in the neonate as an important inclusion. Basic emergency ventilation techniques were discussed. *“… BVM (Bag valve mask ventilation) the patient if something goes wrong”* **Expert no. 2 (Paediatrician).** Participants further discussed more advanced ventilation considerations such as *“something like CPAP, how do we go about doing that?”* and *“The humidification, how the devices work, the limitations in the pre-hospital field and so forth”* **Expert no. 1 (ECP).** Participants further described a NEOPUFF device as an important inclusion under ventilation. *“…doesn’t help you running around with a NEOPUFF and you don’t know how to set it up safely”* **Expert no. 3 (ECP/Nursing sister).** Specific neonatal interventions such as *“How to use your specialized ventilators… methods of ventilation and the air mixtures”* **Expert no. 1 (ECP)** were also discussed by participants. The students also explained that ventilation should be included. *“…how to use the ventilator, ventilation settings”* and specifically equipment like the *“neopuff”* **Student no. 6 (ECP).** |

**Appendix S2: Coding tree for proposed curriculum structure and assessment methods**

| **Sub-category** | **Code** | **Participant quote** |
| --- | --- | --- |
| Course Duration | - Part time post graduate diploma (Proposed by student and expert group) or,] - Part time master’s degree (Proposed by expert group) | Participants also felt that *“There is enough content to learn to justify a full master’s degree”* **Expert no. 3 (ECP/Nursing sister)** for such a specialty. The student group did disagree on the Masters exit level as it will exclude ANT scope ALS providers. *“It should be one year part-time course. It should be a post graduate diploma and not a Master’s degree as the ANTs do not qualify to do the Master’s degree”* **Student no. 3 (ECP).** The student group of participants reflected on the expert group’s suggested duration of education and felt that a post graduate diploma would be ideal. *“I do agree about maybe going about a year or longer”* **Student 7 (ECP).** Participants also felt like it should be completed on a part time basis. “*I don’t have a problem with it following directly after your bachelors because it wouldn’t be a full time thing”* **Expert no. 3 (ECP/Nursing sister)** and *“…a part time course for those people that are still working operationally and would like to increase their knowledge when it comes to neonatal specialized units”* **Student no. 7 (ECP)** would be the best solution to make attending possible. |
| Course Model | - Blended course – Online/Workplace/Simulation-Based Teaching Learning & Assessments - Mentorship programme | Participants gave their opinion on the methods of education they perceive would best suit this kind of course. Participants said that it needs to be interactive. *“It needs something that has opportunity for people to ask questions”* **Expert no. 5 (Neonatologist)** and *“I would say that there needs to be contact time with the student”* **Expert no. 6 (CCA).** Mentorship was mentioned as vital in this kind of education. *“…actual observation and discussion with somebody who has more experience in the world of neonatal transport”* **Expert no. 2 (Paediatrician).** Participants also felt that *“There needs to be theory content but a lot of that can be done online as part of adult learning”* **Expert no. 4 (CCA/Nursing Sister).** Student participants also felt that a theory component should be included. *“… just to level out knowledge across the board, it would be beneficial to have a theory component”* **Student no. 6 (ECP).** It was perceived as best to “*get a specialist to come in and discuss specific topics*” **Expert no. 1 (ECP)** for complex and specialized topics.  Work integrated learning and clinical placement was perceived as very important in this type of education. *“So, in that course I would put, practical time…..on a neonatal ICU transfer vehicle, so that they can get this practical exposure”* **Expert no. 6 (CCA)** and *“The guys need to work on a critical care retrieval service for hands on experience”* **Expert no. 3 (ECP/Nursing sister).** Participants felt that an understanding of the control room processes would also benefit students. *“…and then rotate to the different control rooms, so that they can see the information that they're getting in from hospitals to request transfers, they can see the different resources, understand all those limitations and the decision making there”* **Expert**  **no. 1 (ECP).** Neonatal ICU’s are a limited resource and where these are not available for students to work in *“neonatal units or casualty with doctors and nurses….certainly units where babies are being born”* **Expert no. 2 (Paediatrician)** would be a suitable alternative. *“With our setting, working with specialised teams (CCR) might not be that practical so we might want to look at in hospital exposure to neonates in NICU”* **Expert** **no. 3 (ECP/Nursing sister)** was an opinion on how to increase student exposure to more neonates.  The preferred methods of education were discussed by the participants. They felt that mentorship should play an important role in this education. *“…internship program post qualification … where as you are placed with experienced practitioners for a year, or maybe two years, before you are allowed to make these important decisions on your own”* **Student no. 4 (ECP).** Online learning was also expressed as a practical solution to students who will be working full time. *“… a part time course where they will be able to go in and also have online classes, if they can't go in at school”* **Student no. 7 (ECP).** |
| Resources | - Electronic Devices - Specialist supervision - Cost: self/employer funded | It is important to find out if students will have resources available before developing a course. Participants also felt that electronic devices and internet access to participate in online teaching was not such a barrier any more. *“People, kids in the rural areas have tablets, we have cell phones, like I'm currently using my cell phone”* **Student no. 7 (ECP).**  From a cost perspective, participants felt that employer buy in is very important. *“I think it's imperative for the employer to support this kind of program, because it’s going to be beneficial for both practitioners and their employer”* **Student no. 2 (CCA).** Participants also felt that some students will be able to pay for themselves*. “But if it's something that you personally feel like you will be doing, you can also try and pay for yourself”* **Student no. 7 (ECP).** |
| Targeted Student Group | The course should be inclusive for all Advanced Life Support providers | The student group felt that this kind of education should be inclusive for all ALS providers “*So, I think a course like this, if then allowed afterwards for the CCA and the short courses to have access to, those qualifications where they can now do these transfers (change scope) would be such a huge help”* **Student no. 6 (ECP).** |
| Suggested scope of the course | - Scope of practice should change after course - The course should be considered as an elective speciality for ALS providers in South Africa | The course was also expressed as *“…this should be a specialized field. We're not looking at a one month or two months short course”* **Expert no. 4 (CCA/Nursing sister).** It was also suggested to be a specialty for qualified pre-hospital providers. *“It should be as a standalone course for a specialized team”* **Student no. 5 (CCA).** The student group felt that this kind of education should be inclusive for all ALS providers and that it should change the current scope of practice limitations. *“So, I think a course like this, if then allowed afterwards for the CCA and the short courses to have access to, those qualifications where they can now do these transfers (change scope) would be such a huge help”* **Student no. 6 (ECP).** |
| Formative assessments  Continuous summative assessments | - Work-Based Assessments - Simulation-Based Assessments - Written Assessments | The participants gave their opinion on the methods of assessment. In their experience they *“feel quite strong about continuous learning and continuous assessments*” **Expert no. 1 (ECP).** Discussions around patients were also mentioned as a valuable assessment tool. *“You do need to practically put a baby on a ventilator and move them in an incubator, but for the complex cases you can in hospital go to a patient with transposition (of the great arteries) for example and then discuss how you would move them”* **Expert no. 3 (ECP/Nursing sister).** Participants added on to methods of assessment with oral examinations. *“…you can gauge understanding and insight a whole lot more (with oral exams) when compared to answers from a written test. You can engage with the student and elaborate on the scenario”* **Expert no. 3 (ECP/Nursing sister).** Practically assessing skills were described as *“I would bring in an OSCE station. I mean, this practitioner should be actually very much knowledgeable on using say, an infusion pump or a syringe driver”* **Expert no. 6 (CCA).** Patient simulations were perceived as valuable assessment tool but consideration for the risk on a real patient needs to be considered. *“Another way might be to give them a simulated patient transfer of a real baby in hospital and then you discuss it with them without moving the patient. Then the student can give you his plan. So a combination of simulated and real life stuff”* **Expert no. 3 (ECP/Nursing sister).** Finally, written assessments were described as *“the theoretical written paper needs to be done… for the theory and base knowledge”* **Expert no. 6 (CCA).**  The student group of participants shared their opinion on the assessment. *“I think a more physical approach, your OSCE skills, your actual simulation could prove to show if the knowledge was impacted”* **Student no. 1 (CCA).** The student group also felt that oral assessments add value as an assessment tool*. “… should be more clinical reasoning assessments so that that can maybe be done in form of an oral assessment, a student's capability of reasoning, different topics with regards to treatment, and also problem solving”* **Student no. 4 (ECP).** They felt that written assessments should be included during the foundations phase of this course. *“I think we can begin with written assessments”* **Student no. 3 (ECP).** Continuous assessments by a mentor was also discussed by participants. *“… maybe employ or contract like honorary lecturers, or people that can give feedback regarding the students or to the university”* **Student no. 4 (ECP).** |
| Summative assessments | Portfolio of Evidence | Participants also suggested that the practical component will have to be signed off before completion. *“Which skills are required to qualify for this course….which then you need to demonstrate a portfolio of evidence”* **Expert no. 5 (Neonatologist).** |

# References

1. Venter M, Theron E, Williams W, Khan W, Stassen W. A national retrospective review of neonatal critical care transfers in dedicated critical care transport services in the private sector. South African Medical Journal. 2021;111(10):981-4.
