# Supplementary material for: Developing a South African curriculum for education in neonatal critical care retrieval: An initial exploration
Source: PLoS One. 2023 Aug 31;18(8):e0290972. doi: 10.1371/journal.pone.0290972 (PMC10470938; doi:10.1371/journal.pone.0290972)
Supplement: S1 Data — (ZIP) [file pone.0290972.s002.zip › Data Compressed/Discussion schedule Appendix A.docx]

**Appendix A**

Discussion schedule for:

**INITIATING THE DEVELOPMENT OF A SOUTH AFRICAN CURRICULUM FOR EDUCATION IN NEONATAL CRITICAL CARE TRANSFERS**

*FACILITATION*

*Before the interview/discussion, please refer back to these notes to ensure familiarity with the content. All interviews/discussions should be run by a facilitator. The facilitator is to lead the discussion while taking notes and operates the recording equipment. Each discussion should lead to some conclusions.*

*PREPARATION*

*Test the recording equipment and its sensitivity. The participant consent forms should also be available and ready. Offer the participants refreshment. Make sure that participants are comfortable before the start of the discussion. Ensure that the participants have signed the consent form and that she/he consents to being audio recorded.*

*Read out the statement on confidentiality:*

**There are no right or wrong opinions to any of the topics of discussion. I am here to establish your individual views. Any opinions expressed will be treated in confidence.**

*INTRODUCTION TO THE SESSION*

*Briefly introduce the session. Start by introducing yourself. You may start the session by:*

**I would firstly like to thank you for sparing the time to come and talk about initiating the development of a South African curriculum for education in neonatal critical care transfers. Loosely, the transfer of neonates in South Africa is performed by advanced life support (ALS) providers. This high risk service is reserved for specialist teams internationally. Adverse events during these transfers have been associated with the providers’ level of knowledge. There is currently no specific course in neonatal critical care transfers offered in South Africa. The practitioners that fall under ALS providers have variable education backgrounds. There is no guidance from South African governing bodies on the methods and content of education in this specialised field. The purpose of these interviews and focus group discussions are to establish your opinion on education in neonatal critical care transfers in South Africa. There are no right or wrong opinions, I would like you to feel comfortable saying what you really think and how you really feel.**

*ONE ON ONE INTERVIEWS WITH EXPERT GROUP:*

**Please could you start by introducing yourself and giving a bit of career background.**

| *PROBES AND PROMPTS* |
| --- |
| Demographic data: Age, Gender, Qualification, Location, Position |
| Private versus provincial  Neonatal care and/or critical care transfer experience |

**Understanding the current limitations to training in neonatal critical care transfers within academic and health institutions in South Africa?**

| *PROBES AND PROMPTS* |
| --- |
| - Did the training you received at university or college prepare you for the neonatal critical care transfers that you are currently performing? - What gaps, if any, did you see in the program’s structure or outcomes? - How did you close the gap in education of neonatal critical care transfers if any? - What changes would you recommend in the curriculum that are currently being presented by Universities and colleges? |

**To derive the goals of a curriculum in prehospital practitioner training in critical care transfers of neonates?**

| *PROBES AND PROMPTS* |
| --- |
| - Do you think additional training in neonatal critical care transfers are needed for pre-hospital providers? - How long should such training be? - What method of training would you advise? - What core competencies i.e.: knowledge, skills, and attitudes should a neonatal critical transfer practitioner graduate with? |

**What would you say are the specific needs to teaching, learning and assessment (TLA) as an expert in neonatal critical care transfer education?**

| *PROBES AND PROMPTS* |
| --- |
| - Refer to neonatal data presented in background reading document - Do you agree with the patient presentations from Venter’s study when compared to the patients that you manage? - What knowledge, skills both clinical and non-clinical necessary for TLA of these patient categories? - Any broad categories you would include in addition to these findings? - The literature review yielded the following core modules that should be included in the curriculum. Do you agree? - Refer to core modules presented in background reading documents - In your opinion, what are the best strategies for assessment in achieving the competencies you outlined above? |

Final Reflection

- What is the most important thing you would like to tell the curriculum committee as they work towards developing a curriculum in neonatal critical care transfers?

*FOCUS GROUP DISCUSSION WITH LEARNERS:*

**Please could you start by introducing yourselves and giving a bit of career background.**

| *PROBES AND PROMPTS* |
| --- |
| Demographic data: Age, Gender, Qualification, Location, Position |
| Private versus provincial  Neonatal transfer experience |

**Understanding the current limitations to training in neonatal critical care transfers within academic and health institutions in South Africa?**

| *PROBES AND PROMPTS* |
| --- |
| - How much time was spent on the topic of neonatal transfers during your training at university or college? - Did this training prepare you for the neonatal transfers that you are currently performing? - How did you close the gap in education of neonatal transfers if any? - What changes would you recommend in the curriculum that are currently being presented by Universities and colleges in neonatal transfers? |

**What would you say your specific needs are in learning and assessment (TLA) as a learner in neonatal critical care transfer education?**

| *PROBES AND PROMPTS* |
| --- |
| - Do you need additional training in neonatal critical care transfers? - If so, which specific areas of knowledge, clinical and non-clinical skills would you like further training on? - How much time could you spare for such training? - Do you have financial support to pay for additional training? - What method of training do you prefer? - Would you be able to travel to another city for such training? - Do you have access to a computer and an internet connection? - Do you have specific needs as it pertains to language, religion or culture that will have to be considered during training? |

**To derive the goals of a curriculum in prehospital practitioner training in critical care transfers of neonates?**

| *PROBES AND PROMPTS* |
| --- |
| - Experts in the field of neonatal care and critical care transfers have suggested the following core topics to be included in a new neonatal critical care course. - INSERT SUGGESTED CONTENT BY EXPERTS - Experts have suggested the following methods and duration of education and assessment for this course: - INSERT SUGGESTED METHODS AND DURATION OF EDUCATION AND EXAMINATION BY EXPERTS - Do you agree with the suggested methods and duration of education and examination for such a course? - What would be a reasonable cost of such training? - Would you travel for such training? |

Final Reflection

- What is the most important thing you would like to tell the curriculum committee as they work towards developing a curriculum in neonatal critical care transfers?
