# Supplementary material for: Developing a South African curriculum for education in neonatal critical care retrieval: An initial exploration
Source: PLoS One. 2023 Aug 31;18(8):e0290972. doi: 10.1371/journal.pone.0290972 (PMC10470938; doi:10.1371/journal.pone.0290972)
Supplement: S1 Data — (ZIP) [file pone.0290972.s002.zip › Data Compressed/Background reading Expert Group .docx]

**Background reading**

**INITIATING THE DEVELOPMENT OF A SOUTH AFRICAN CURRICULUM FOR EDUCATION IN NEONATAL CRITICAL CARE TRANSFERS**

**Introduction**

I would firstly like to thank you for sparing the time to participate in the study “Initiating the development of a South African curriculum for education in neonatal critical care transfers”. Loosely, the transfer of neonates in South Africa is performed by advanced life support (ALS) providers. This high risk service is reserved for specialist teams internationally. Adverse events during these transfers have been associated with the providers’ level of knowledge. There is currently no specific course in neonatal critical care transfers offered in South Africa. The practitioners that fall under ALS providers have variable education backgrounds. There is no guidance from South African governing bodies on the methods and content of education in this specialised field. The purpose of these interviews are to establish your opinion on education in neonatal critical care transfers in South Africa.

To inform our discussion, please review the following information:

**What type of neonatal transfers are being conducted in South Africa?**

**Please keep the following information confidential until we have published**

A retrospective chart review that was conducted by M. Venter determined the national patient population of critical care transfers between 01 January 2017 and 31 December 2017 in South Africa. Below is an overview of the data from the n=444 transfers.

| **Gender distribution** |
| --- |
| **Time spent with patient** |
| **Average number of attachments per patient n=4** |

**Top 30 diagnosis distribution for this group**

**Top 30 medications administered**

**Attachments and/or interventions**

**Special group sub-analysis**

Neonates with congenital heart defects (CHDs) are complex cases that justify separate analysis. When we group the CHD cases together we have the following findings:

**Distribution of CHD conditions**

**Demonstration of some complex cases**

**The following tables are cases that were extracted from the cohort of neonates. The aim is to demonstrate the complexity of some of these CHD transfers.**

**Case 1**

| **Diagnosis** | **Transposition of great vessels** |
| --- | --- |
| **Medications** | - Dopamine - Dobutamine - Adrenaline - Prostin - Hep-Saline - Neonatelyte |
| **Attachments** | - ET Tube - Ventilated - Umbilical line - IV - Urine catheter - 3 Lead ECG - Incubator - Infusions |

**Case 2**

| **Diagnosis** | - **Persistent pulmonary hypertension** - **Right Ventricular Hypertrophy** |
| --- | --- |
| **Medications** | - Milrinone - Dopamine - Hep Saline - Sodium Bicarbonate |
| **Attachments** | - Ventilated - IV - 3 Lead ECG - O2 Saturation - Urinary Catheter - Infusions - NT Tube - Umbilical arterial and venous line |

**Case 3**

| **Diagnosis** | **Coarctation of Aorta** |
| --- | --- |
| **Medications** | - Dobutamine - Sodium Bicarbonate - Midazolam - Insulin - Prostin |
| **Attachments** | - Nasotracheal tube - Mechanical ventilation - Nasogastric tube - Central Venous Line |

**Literature review findings regarding local and international neonatal critical care transfer education**

The aim of the literature review was to find and critically appraise current literature in the field of neonatal critical care transfer education. The first strategy included searches on the Google Scholar and PubMed platforms for peer reviewed literature. The second strategy included grey literature searches on the Google platform to include neonatal courses and their curricula. The peer reviewed literature was assessed for reliability and validity during the appraisal process. The search strategy was narrowed down to literature from the past decade (2010 – 2020) which were published in English.

This literature review found that there is a paucity of literature in the field of neonatal critical care transfer education especially within the South African context. The following section is a summary of the curricula that can be associated with neonatal critical care transfer education in the local and international context.

**South African curricula**

The current curricula in critical care presented by three of the South African universities in BEMC were compared in a study by Conradie et al. The study found that one of the universities differed largely from the other two in its curriculum design. The theme of critical care presented by these Universities include neonatal transfer content. The curricula are described in the following table:

| Content or competency descriptors (Usually by accreditation bodies) |
| --- |
| General:   - Need for ICU - Function of ICU units - Pressure ulcers treatment - Maintaining neutral thermal environment - Care of IC drains and wound dressing   Ventilation   - Indications for ventilation - Difference between vol, pressure time cycled - Explain and compare following vent modes: IMV, CMV, SIMV, BiPAP, APRV, - Manipulation, - Set up - Monitoring: Ventilation rate, Tidal volume, Minute volume, Flow, PEEP, I:E ratio, Trigger, Pressure support, Peak airway pressure, plateau pressure, slope, FiO2 - Integrated modes and ventilation settings - Ventilator graphs - Ventilation alarms   Patient monitoring   - SPO2 - Capnography - Troubleshooting - Risk factors of VALI - Weaning of patients from vent - NIV – indication, contra-indications, modes - Set up of NIV   Arterial blood gas   - Role and interpreting of ABG - Obtaining ABG sample   Monitoring   - ECG - Arterial oxygen saturations - ETCO2 - NIBP - Temperature - Haemodynamic monitoring - Role and management of central vascular access   Infusions   - Flow rates and drug dose calculations - Use and troubleshooting of infusion devices - Role and management of nasogastric feeds - Role and management of TPN   Fluid balance   - Fluid requirements and choice according to various disorders - Fluid balance monitoring   Imaging   - Chest X-Ray   Preparation for transfer, transfer and handover   - Patient assessment - Accumulation of data and history taking - Patient packaging - Decision making in prep for ICU transfer - Haemodynamic changes - Stressors of transport - Patient handover   Special populations   - Neonates not mentioned   Obstetrics and gynaecology emergencies   - Conception to birth (Should include foetal circulation and the transition after birth) - General obstetric emergencies and their management. |
| Program structure (subjects, units etc.) |
| The modules of critical care consisted of intensive care, thrombolytics, aeromedical transportation and dive emergencies. It is interesting that neonatal critical care transfers were not a stand-alone discipline. The one university opted to incorporate the critical care curricula with other modules and the other two universities present it as a stand-alone modules |
| Learning activities (Lectures, workshops etc.) |
| Lectures were the main platform with some online content delivered as a supplement. The online content varied from pre-reading material to videos and tutorials. Information technology used by the universities were the Blackboard and Moodle platforms to deliver online content. Work integrated learning was another form of acquiring knowledge and skills in the field of critical care. The learning sites did include Neonatal ICU for all three universities. |
| Assessments (Tests, clinical examinations) |
| The assessment component was divided into practical and theory. Practical assessments consisted of patient simulations and OSCES. The theory evaluations were divided into different sub-components with different weightings. The weightings and sub-components varied widely between the universities. The theoretical assessments consisted of assignments, written tests, simulations, orals and OSCES. |
| Schedules (Date, time, location) |
| Full time study as part of the BEMC qualification. The critical care curricula are presented over either a full year or semester period. |
| People (Students, faculty etc.) |
| The first university employed four staff members (2 x PhD in EMC, 1 x BEMC, 1 x Prof. Nurse), the second 1 x PhD in EMC and the third 1 x BEMC. One could interpret this as a different importance weighting per university on the subject of critical care |
| Resources (Teaching materials, equipment etc.) |
| Not described |
| Course evaluations (By students, faculty and external bodies) |
| Not described |
| Learning portfolios |
| Assessment for the integrated learning was in the form of case studies, oral presentations, reflective journals and patient report forms. |
| Financial information |
| Cost not specified as it forms part of the 4 year BEMC programme. |

###

### International neonatal critical care curricula

Paediatric and neonatal critical care transport is a book published by BMJ books and cited as the only manual of neonatal and paediatric transport practice for the UK. This book only provides content and not a full curricula, which will be specified by the training institution.

Paediatric And Neonatal Critical Care Transport

| Content or competency descriptors (Usually by accreditation bodies) |
| --- |
| Part 1: Planning for safe and effective transport   - Principles of safe transport - Transport physiology - The ambulance environment - Equipment and monitoring - Air transport of critically ill children   Part 2: Practical transport management   - Neonatal resuscitation and stabilisation - Paediatric resuscitation and stabilisation - Management of the airway and breathing - Management of the circulation - Trauma - Special transport interventions - What to do when it all goes wrong - Drugs - Typical retrieval forms - Essential equations |
| Financial information |
| Book cost $64-50 |

NAPSTaR (Neonatal, Adult and Paediatric Safe Transfer and Retrieval) is a short course for medical professionals involved in transfers, presented by the Advanced Life Support Group in the UK. The curriculum is described in the following table:

NAPSTaR (Neonatal, Adult and Paediatric Safe Transfer and Retrieval)

| Content or competency descriptors (Usually by accreditation bodies) |
| --- |
| - Introduction to ACCEPT (Assessment, Control, Communication, Evaluation, Preparation and Packaging and Transportation) - Introduction to communication and Human Factors - Why are transfers different? - Preparation, packaging and transportation Workshops/skill stations - ACCE - Equipment tips, tricks and pitfalls - Communication Discussions - Movement and safety - Mobile environmental physiology - Untoward events |
| Program structure (subjects, units etc.) |
| Single unit short course |
| Learning activities (Lectures, workshops etc.) |
| Lectures taught in class with practical workshops, skill stations and simulations |
| Assessments (Tests, clinical examinations) |
| Continuous assessment during course but methods not specified |
| Schedules (Date, time, location) |
| Two day face to face course presented in the UK |
| People (Students, faculty etc.) |
| Students from medical, nursing and paramedic backgrounds. Faculty not specified. |
| Resources (Teaching materials, equipment etc.) |
| Not specified |
| Course evaluations (By students, faculty and external bodies) |
| Not specified |
| Learning portfolios |
| None |
| Financial information |
| Unknown |

This course appears to be more of an introduction into neonatal transfers but does cover some essential topics.

Advanced Paediatric Life Support (APLS) is a short course that has been adopted by the European resuscitation council as its prescribed training for medical professionals that manage paediatric patients.

Advanced Paediatric Life Support

| Content or competency descriptors (Usually by accreditation bodies) |
| --- |
| - Structured approach - Human factors - Recognition of seriously ill child - Child with breathing difficulties - Child in shock - Abnormal pulse rate or rhythm - Decreased conscious level - Convulsing child - Seriously injured child - Chest injury - Abdominal injury - Head injury - Injury to the spine - Burns and scalds - Drowning - Cardiac arrest - Stabilisation and transfer - Assessing & managing the serious ill child |
| Program structure (subjects, units etc.) |
| Single short course with no subjects |
| Learning activities (Lectures, workshops etc.) |
| - Lectures - online pre-preparation - skill stations - workshops - scenarios |
| Assessments (Tests, clinical examinations) |
| - Multiple choice questions - Basic Life Support - Airway management - Scenario |
| Schedules (Date, time, location) |
| One day online and two day face to face training course |
| People (Students, faculty etc.) |
| Faculty not specified. Student candidates from medical and nursing backgrounds. |
| Resources (Teaching materials, equipment etc.) |
| Not specified |
| Course evaluations (By students, faculty and external bodies) |
| Student feedback required  Supervisory visits at training institutions |
| Learning portfolios |
| None |
| Financial information |
| 450 GBP for the two day course |

This course does cover some essential content for neonatal critical care transfer education but is more structured for in hospital users with emphasis placed on resuscitation and trauma.

The Paediatric advanced life support for experienced providers (PALS) course is a two day short course by the American heart association presented by training centres world-wide.

Paediatric advanced life support (PALS)

| Content or competency descriptors (Usually by accreditation bodies) |
| --- |
| - Review of BLS and AED for infants and children - Systematic approach to the seriously injured child - Recognition and management of cardiac arrest - Effective resuscitation team dynamics - Recognition of respiratory distress and failure - Management of respiratory distress and failure - Resources for management of respiratory emergencies - Recognition of shock - Management of shock - Recognition of arrhythmias - Management of arrhythmias - Post cardiac arrest care |
| Program structure (subjects, units etc.) |
| Short course |
| Learning activities (Lectures, workshops etc.) |
| Two day face to face course with online pre-course preparation |
| Assessments (Tests, clinical examinations) |
| - Online pre-course assessment - Multiple choice test in class - Simulations - Skills stations |
| Schedules (Date, time, location) |
| Two day course presented across the world |
| People (Students, faculty etc.) |
| All medical professionals interested in paediatric care |
| Resources (Teaching materials, equipment etc.) |
| Online content  Videos  Books  Practical scenarios with medical dolls and training aids |
| Course evaluations (By students, faculty and external bodies) |
| By students after course (questionnaire) |
| Learning portfolios |
| None |
| Financial information |
| R3300 |

This course covers essential information that can relate to neonatal critical care transfer education, but it does not cover in depth content and specialised equipment often used in these transfers.

The Essentials in neonatal critical care orientation (ENCCO) course is an online course offered by the American Association of Critical Care Nurses (AACN). The course is aimed at the nursing environment and has a more in depth approach to neonatal critical care compared to some of the short courses reviewed.

Essentials in neonatal critical care orientation (ENCCO)

| Content or competency descriptors (Usually by accreditation bodies) |
| --- |
| \| Module 1 – Patient Care   - Extrauterine Life: Fetal to Neonatal Circulation - Extrauterine Life: Assessment of the neonate’s transition - Comprehensive Assessment of the Neonate - Developmental Care of the Newborn - Family-Centered Care in the NICU - Maternal Factors Affecting the Newborn - Neonatal Abstinence Syndrome - Neonatal Nutrition - Neonatal Pain Assessment and Management - Neonatal Skin Care - Neonatal Pharmacokinetics - Thermoregulation of the Newborn Infant   Module 2 - Respiratory Care   - Apnea of Prematurity - Bronchopulmonary Dysplasia - Meconium Aspiration Syndrome - Pneumothorax in the Neonate - Neonatal Respiratory Distress Syndrome - Transient Tachypnea of the Newborn   Module 3 - Infectious Diseases   - Neonatal Sepsis   Module 4 - Neurology   - Neonatal Cranial Hemorrhage - Neonatal Seizures   Module 5 – Metabolic Function and Disorders   - Glucose Homeostasis in the Neonate - Hyperbilirubinemia - Metabolic Disorders of the Newborn   Module 6 – Neonatal Vascular Access   - Central Lines in the neonate \| \| --- \| |
| Program structure (subjects, units etc.) |
| Six units:   - Patient Care - Respiratory Care - Infectious Diseases and Management - Neurology - Endocrine - Neonatal Vascular Access. |
| Learning activities (Lectures, workshops etc.) |
| Online content in the form of videos and lessons |
| Assessments (Tests, clinical examinations) |
| Online tests |
| Schedules (Date, time, location) |
| One year |
| People (Students, faculty etc.) |
| Nurses that want to work in the NICU |
| Resources (Teaching materials, equipment etc.) |
| Online content |
| Course evaluations (By students, faculty and external bodies) |
| Not specified |
| Learning portfolios |
| None |
| Financial information |
| $468-00 |

**Conclusion**

This concludes the essential reading section that will inform our discussion on neonatal critical care transfer education in South Africa. We look forward to your participation and valued input as an expert in the field.
