# Supplementary material for: Developing a South African curriculum for education in neonatal critical care retrieval: An initial exploration
Source: PLoS One. 2023 Aug 31;18(8):e0290972. doi: 10.1371/journal.pone.0290972 (PMC10470938; doi:10.1371/journal.pone.0290972)
Supplement: S1 Data — (ZIP) [file pone.0290972.s002.zip › Data Compressed/Transcript 1.docx]

**Interview 1**

**Researcher 1**

We are recording now. So just to confirm again, while we recording. Do you consent to us recording the session?

**Participant 1**

Yes, I consent.

**Researcher 1**

Thank you very much. And then you receive the consent form I sent you.

**Participant 1**

A Yes, I have. Can I sign it and send it to you?

**Researcher 1**

Okay, perfect. So just to quickly summarize that, Louis, so this interview is voluntary, and you can withdraw at any time. And that if there's any personal information, it will be anonymized during the transcription. Are you are you happy with it?

**Participant 1**

I'm happy with it and I have no further questions on that.

**Researcher 1**

So, just a quick background to the study. So, as you know, the transfer of neonates in South Africa is performed by advanced life support providers. And that this is a high-risk service. And it is usually performed by specialized teams Internationally. And that adverse events during these transfers, it's been linked to the providers level of knowledge. And then in South Africa, as you know, the ALS providers, they have a variable education background. And there's also no guidance from South African governing bodies on the method and the content of education in this specialized field. So that's the very brief background to this. So, today, I'm after your opinion on education in neonatal critical care transfers, and there are no right and wrong answers to that. And so, to start, can you please briefly tell me about your background, where do you work and your education and more specifically, your experience with neonatal transfers.

**Participant 1**

So just to start off with my experience with neonatal transfers, I worked on the critical care retrieval service, which the majority of the work included neonatal critical care retrieval from across the province, and from various outlying facilities as well as Metropolitan facilities, and then mostly transferring these critically ill to specialized neonatal facilities. Then I moved over to a university, I'm teaching on the fourth-year program for the last three years. And on the fourth-year program, the neonatal training curriculum is in there. So, part of my job is to train the fourth-year students in the neonatal outcomes set up by the program.

**Researcher 1**

So, tell me, your experience with the retrieval unit was that from when to when?

**Participant 1**

So, I think it was 2013 to 2016 that I worked on the critical care retrieval vehicle, not 100% sure, but I think it was about 3 or 4 years there.

**Researcher 1**

And, can you please tell me a little bit about your education?

**Participant 1**

So, education, I did my national diploma, and then worked on road ops. And then I did my B tech straight after my diploma and completed my B tech. Other training included the critical care retrieval course. We also did the PCC TPC exam, which was quite interesting. registered with that. Also, that critical care nursing which included a neonatology block essentially. Did NALS, PALS instructor what else was there? I think that's about it. Remember something also bring it up again.

**Researcher 1**

Okay, great. So, you were working on the ccrs, vehicle 2012 to 2016. And you currently working at university on the fourth-year program and teaching neonatal curriculum, and then said your education started as an N.Dip and that was followed by your B-Tech. And also did some critical care retrieval courses, and critical care, nursing and then some other short courses like pals and so on? Is that correct?

**Participant 1**

Yes, that's correct. All right.

**Researcher 1**

So, tell me when we look at your background in education and your transfer of neonates, if you had to highlight which of this type of training was the most valuable for you, when you were transferring neonates,

**Participant 1**

I think very, very little of the training that we actually did, prepared me for that first neonatal transfer we ever did as part of a critical care retrieval program. I think the shock and awe factor of transferring that first really sick intubated, ventilated neonates just shocked you to the core, and just shows you your amount of knowledge gaps that you actually have. No matter how much training you you've had, I think the nice thing was that I immediately realized that there's quite a large knowledge gap that needs to be filled, and then tried to go and find that information some way to upskill myself, to prepare me for, for doing it in a safe manner. So, I think from all the training, the original training that I had, before I walked into critical care retrieval, which was the diploma and B-tech, added very little to none preparation, and very little to no preparation for actual neonatal transfer medicine. There were some basic things like understanding of neonates and, and some basic assessments, but nothing close to appropriately ventilating and taking care of a neonate. I think a lot of that was missing in the beginning.

**Researcher 1**

All right. So, you refer to your earlier training, so it started as N.Dip and then B-Tech, this is obviously changed to a full-time bachelor's program. Would you say that? You said this didn't prepare you? Do you think the current program that's running? Do you have information about it? Do you think it's changed? Or do you think it's still similar?

**Participant 1**

Yeah, absolutely. I mean, after being at the university, now, for a couple of years, it's quite interesting, seeing the background, seeing how things have changed. And even sitting down and talking to the lecturers that I had back then, and just, you know, talking with them about the absolute knowledge gaps that we had when we left. So right now, for me standing in the university side, there's a good understanding of, you know, where the gaps were. And I do feel that where the program is, at the moment, or at least the program that we're running at the moment does have, in some, some preparation for future graduates. The only problem with the limitation that we have is essentially time. You would want to do so much more. But we are so restricted with time and resources, that it's quite hard to, to get the students where they would need to be where, I feel they would need to be for neonatal critical care transfer.

**Researcher 1**

Alright, thanks. So, just to go back to your earlier days, and you said there were gaps in you would try and find material and close these gaps, what type of sources that you look for during that time? And how would you How would you try and close those gaps back in the day?

**Participant 1**

My main port of call was neonatal ICU and sitting down and chatting with the neonatologist and the neonatal critical care Nurses within those units, it was an incredible way to go about doing it because you sit down and have a conversation with neonatologist and he'll, you'll quickly during the conversation realize that there's quite a lot of gaps from basic knowledge there. So that would highlight those issues. And then as you spend more time within those, those units, and with the with the nurses within those units, you realize this, there's quite a bit more that their focus is on. So, it was great to see those gaps. And but the problem I still had was understanding those gaps and understanding that they have quite a bit of knowledge within those units. None of those prepare you for the actual transport because there's still quite a lot of things that occur during transport that you will have to consider different manner. So that was where with that biggest issue was. And then obviously some literature searching from a lot of the American stuff helped fill some of those gaps.

**Researcher 1**

Alright, so then just to recap, you, you said that your initial knowledge base that you received, on N.Dip and B.Tech was limited didn't prepare you for neonatal transfers. And then your exposure to neonatal ICU and neonatologists helped you understand the gaps that you had, and then you try to fill it with whatever information you could get hold of.

**Participant 1**

Yeah, so I wouldn't call it exposure, I would I would say it was it was more of a specific I had to go to the unit and started discussions. And I feel that's important to mention, because if a paramedic just feels that they don't need to fill the knowledge gap, or they feel that they don't have a knowledge gap. That might actually be more of an issue. But I do feel it, it's important to note that I had to go and sit down and say hi, I actually have a problem. Understanding issue, I would like to understand more from neonatal ICU, if that makes sense.

**Researcher 1**

Makes perfect sense. So, what you're saying is that you didn't initiate this contact with them yourself? And if you didn't seek answers or help, it probably would not have happened.

**Participant 1**

Yes.

**Researcher 1**

Right. So, we spoke a little bit about the transition from the old curriculum to now for B-tech, and you are still involved in that program. And if you could change the curriculum, you mentioned that the problem is obviously limited time, there's only so much that you can do in four years, is there anything you would change in the current curriculum with the time that's available?

**Participant 1**

So just to rectify that, it's not over four years, it's essentially, I'm looking at my timeline now. And would have had, if 2020 was normal, I would have had about two months, just on neonatology. They don't touch a neonate or Paed or anything in four years and when they come to fourth year, that's the first time they get exposure to new Neonatology. And there's an it's the way the program's structured is that it uses scaffolding to, to prepare students to come up from first year getting all of the adult things together. But the way the program is structured now is paeds and neonates are only taught from the for the first time and first exposure only in fourth year itself. So, if I were to change things, it's it would be exposure from their second or third year already so they can at least just have start seeing the exposure. start understanding that this is different population of patients out there. So that when you when they get to 3rd and 4th year and you start with these things that then they're not completely oblivious about it, then I would insist in a lot of in hospital based training is it's all good and while we do some of the training and prac lab, but if you don't see and work with that kid and see how they work with that fragile prem neonate in NICU it becomes quite hard to explain how to do those things, then the ventilation under neonatology would want to spend quite a lot more time and a lot more in depth discussions and preparation for the students. Yeah, and then I would want to spend a bit more time on neonatal specific critical care retrieval. Right now, on our program, critical care retrieval is sort of separate and we add some neonates in it, but it's not very neonate specific. So, we'll do within that short period of time for my critical care retrieval, I have to do adults, paeds and neonates. So, it does make it quite difficult. And the training for neonatal critical care retrieval is quite limited in that regard. And because of our student numbers, I have 45 students that I have to push through that section. And to do one on one sims and training with it with the students, that record becomes quite difficult and quite diluted when you're doing in large groups. So sorry, I did jump around quite a bit there.

**Researcher 1**

Yes, thanks. So, to for me to just quickly summarize that section. So, on the current bachelor's program, you say that the neonatology section is covered in the fourth year. And it's a two-month block before that there is not much exposure that builds them up to this. And it's just the structure of the course and what the vast amount of information that needs to be covered. And so, you said if you could change anything within this program, you would suggest maybe earlier exposure to neonates from let's say, second or third year, and in hospital would be a good starting point. Because of the type of patients, they see and it's a safer environment. And then you said ventilation requires additional time, because that's quite a high acuity skill. And then another problem that you have is the limited time for neonatology section and then also the number of students that you need to get through some of it tends to be a bit generic and not really specific to neonates and packaging, preparing and transferring.

**Participant 1**

Yeah, that's, that's correct. I think I just need to add in that, it's quite important to understand that because of the way that the program is structured, we're not we're not preparing a paramedic to be a dedicated critical care neonatal retrieval specialist. Unfortunately, the program is not designed in that way. And if I would have it my way I would, I would highly recommend that. And from reading some of the literature and the understanding of, of Critical Care Medicine, I would really highly recommend the use of like a postgraduate diploma or a course or even a degree would be great to prepare a paramedic to do adverse event free critical care, retrieval of neonates, and so forth. The training that we do, and the curriculum, as it stands, is quite limited in doing that. And I think the reason we add all of these things in here is because we know at the moment, in our EMS structures and in the industry, our guys don't really have a choice and they will have to just do the neonatal retrieval, medicine. So, we do feel it's quite important to add most of these things in there to try and prepare them as best as we can with a limited time, such as film is important to have add that in.

**Researcher 1**

Alright, thanks. So yeah, just to summarize, you say that because of the program's limitations, you the output is a practitioner that can transfer a neonate, but the course is not designed in such a way that you have a critical care retrieval specialist for the very complex cases.

**Participant 1**

Yeah, that's correct.

**Researcher 1**

Okay, thanks. So, I think you've answered my next question. The question is, do you think additional education in neonatal critical care transfers are needed for advanced life support providers? So, this is not only for your program, but also ALS practitioners in general?

**Participant 1**

And before I answer I see, Researcher 2s, hands up.

**Researcher 2**

Thank you, I think that you're starting to move to towards what I was going to ask, but I think, would you be able to just expand a little bit more on your idea with regards to what is safe practice for, for a new graduate because it sounds like you're saying that there's certainly some need for new graduates to get more training in neonatology. But then that there is an there is an additional qualification that's required for you to now start moving critical care, neonates, but maybe before we move on to critical care, neonates, I just like to get a little bit more information with regards to what you consider to be sort of safe practice for a new graduate and the requirements for that.

**Participant 1**

Sure thanks. So, I do feel and what we are currently doing is, which is part of the curriculum is to do a neonatal resuscitation. So, doing a full neonatal resuss from beginning through straight through to end feel, it's quite important that they have a really good understanding of that, because if they would transfer neonates, they would need to do a neonatal resuss, either on road ops, or during transfer that be found competent in that before starting to work. And then for them to have a really good base understanding of the basic, the really good understanding of the general pathophysiology’s that we would find in neonates. So, if I can read it to you from our program, our focuses are on RDS, meconium, aspiration, congenital pneumonias, under ventilation, air leaks, dehydration, hyperglycemia, infection, sepsis, trauma, including birth trauma, neuro abnormalities and seizures. And then under transport things we talk about infection controls, adverse events, systems, skincare of neonates, handling of neonates, and mitigating noise and vibration during transport, feeding of prem neonates. We also talk about the appraisal of retrieval in ground and air ambulances. We talk about limitations and current transport incubator technologies. So, how to manage transport incubators. And then we do discussion management of congenital abnormalities, brain, spinal cord, GITs, and renal systems. So, I think that that what we do cover at the moment has a good fit. Oh, sorry. And then I've got neonatal assessment. So, assessing birth weight, and then different methods, medical history of the neonatal patient have a proper physical exam, nearly one infant targeted history. And prematurity. And I do think what your original question was, what we think is, is there is the base that they would need for qualifying right now. I feel it's being able to assess a neonate appropriately prioritize what's the most important now for that patient before moving them and also realizing and this is quite important, what we're driving quite a bit is, is realizing whether or assessing whether transferring that neonate with equipment that they have or the skills and process that they have in place and then in within the setting would be appropriate or not. So, we drum down a lot in the decision making to try and prevent adverse events. And in that regard, I think it's quite important to, for them to have a really fairly good base knowledge so that we have enough knowledge to be able to assess Whether it be safe or whether they would be capable of transferring that neonate or not. Does that answer your question?

**Researcher 2**

Thank you. I appreciate.

**Researcher 1**

All right. Thank you for that. So, let me just quickly summarize in that section. What would you consider safe practice? How would you ensure that base knowledge and for the new practitioners? So, you said your cover neonatal resuscitation, and then you get quite a good list of pathophysiology that needs to be covered like RDS, meconium, and air leaks during ventilation, seizures, and so on. And then aspects of transfer like skin care, handling, and so on. And then appraisal of transfers, whether you should be air or road, and then your limitations for the incubator, and also the handling of the incubator setting and so on. And then you mentioned the importance of assessments of a neonate. So, you need to be able to assess a neonate for the different diseases and so on and how to assess a neonate correctly. Is that right?

**Participant 1**

Yes, that sounds correct.

**Researcher 1**

Okay, thank you. So, moving on to the next question, then, we've had quite a bit of a discussion on the limitations of the current program and what can be done with timeframes and so on. So, my question, then would you suggest that advanced life support paramedics receive additional training,

**Participant 1**

I do suggest that practitioners get additional training for neonatal transport. And the reason I say that it's quite a specific thing, there's a lot of things that can go wrong. It's also if a paramedic works on the road, they're not going to see a neonate every day. You're working on ambulance, and you might get dispatched to a neonatal transfer once or twice a month. The other issue is that some services are starting with their dedicated critical care retrieval services. And then road ops, paramedics might not even see a neonate, for years, and then one day all of a sudden, would be expected to transfer a neonate because of resource constraints. So, before the training, I do think there needs to be a better understanding and a better system in place to say, to prevent adverse events in neonates, we do need to have dedicated teams, we need to have dedicated equipment and so forth, that all those all those points. And that dedicated unit or retrieval, paramedics need to go and transport these patients. So now those guys, once we have all those systems in place, those guys would need to have extensive postgraduate training or training afterwards. And not just a once of training, it needs to be continuous training included with the quality assurance systems in place as well, so that there's continuous learning, and not just a once off thing that needs to be absolute continuous training, with time spent in neonatal ICU as well.

**Researcher 1**

Alright, thanks for that. So just to summarize, then, your answer is then yes, we do need additional training. And the reason being because this patient population is very specific, and the guys on the road don't see neonates often. And when they do, it might be a bit overwhelming. So, you suggest to cover this area that's lacking dedicated teams. And then the individuals that do operate on these teams, they need specialized training. So, if we could say that advanced life support providers or these specialized team members for critical care retrieval teams, if you suggest that a postgraduate training needs to be continuous and there needs to be quality controlled exposure in NICU environments and so on? How long would you suggest this type of training should be?

**Participant 1**

I have no idea or any suggestions. What I would want to say, and just from my experience with working and in undergraduate training, is that before you can put on a timeline and say this is how much time we would need to do the training, I do think it'd be vitally important to just sit around and say, Okay, well, these are the things that we would need to focus on. These are the notional hours that we would need to spend on getting towards this point, to this outcome, and work on a program on that, whether it be a year program or a two-year program. Whether it's part time or full time, I do feel that it should be a program that has continuous learning. So, guys are working with another paramedic while they're doing the training program included into work integrated learning, so that it's not just this synthetic type of training, and in a classroom, it needs to be integrated in the healthcare setting. Pre-hospital both for your hospital and aeromedical, control room, hospital based, the training needs to go through all of those aspects. And then a rotation basis or not. Yeah, so I, I don't really feel comfortable giving a timeline saying a year or two years, I do feel that it's important to Firstly, sit down, come up with those outcomes and come up with a program and then be able to calculate those notional hours and figure out the timeline.

**Researcher 1**

Okay, thanks for that. I appreciate that. So, it's not possible to put a timeframe to this. And with your experience in this field, you're saying that first objectives and outcomes for this type of education needs to be determined. And after that a team can decide on the notional hours allocated to this? Is that correct?

**Participant 1**

Yes, that's correct. I mean, I do feel if you make it a postgraduate diploma, there's certain national hours, so either be a two-year program or a one-year program. So, I do feel that it depends on what it is that we would want to achieve by this. And I do feel that the way our industry is working and the way recognition is acquired, and for services to have a better understanding and better support for this type of thing. I do feel it's important to have a well registered and recognized program, instead of just doing, you know, type off a quick short course thing in it. So, I do feel it's important to do that.

**Researcher 2**

Yes, thank you. I just like to get your opinion on. You mentioned for it to be actual postgraduate qualification. And I just like to get your opinion on how you see that sort of working with regards to where we are seated with the different types of both ALS qualifications and otherwise, within the South African context. So, I guess what I'm saying is, if it's postgraduate, like postgraduate diploma or master's level, how do you see that relating to some of the different types of, of qualifications that we have in South Africa?

**Participant 1**

That's a very nice question. Thank you. I'm not actually 100% sure, and I completely agree. I mean, there's they are pre-hospital providers that have been providing neonatal transport for very many years. And whatever program is set up, needs to include all of the different structures. So, I guess if you include if you want to make it inclusive for everybody, and there needs to be a program set up to ensure base knowledge is covered first, and then moving on. So, considering doing a diploma for it would probably be a consideration. Yeah, that's where I'm standing at the moment.

**Researcher 1**

Okay, thanks. So just to quickly summarize that, so your question was, if there was a postgraduate qualification, whether it be diploma or master's level, I would this fit in with our various ALS qualifications. And to make it inclusive, it will have to be a starting point to ensure the base knowledge is covered, and potentially maybe a diploma level. Is that correct?

**Participant 1**

That's correct.

**Researcher 1**

All right. Thanks, you did touch on this earlier, if we had to, in your experience, look at the method of education. So how would we do this type of postgraduate education? Would it be in class or online? Can you elaborate on that a little bit, please?

**Participant 1**

So, from my experience, especially on the critical care retrieval training, I do feel it's, it's quite difficult to just stand in a class and do this this type of training, I do feel it needs to be a hybrid-based training, that's integrated within different healthcare settings. I do also feel that because it's such a specialized thing, we would need to get a specialist to come in and discuss specific topics. I don't expect a neonatologist to come in and tell us about neonatal transportation. But I do feel it'd be important for neonatologist to come and talk about congenital abnormalities and what is being done in hospital and so forth. So, when it comes down to the lectures that you feel it needs to be online based, that's asynchronized online based training. And then it needs to be an integrated portfolio building training program. So, they would have to work in an ambulance or in a setting where they can see patients continuously and rotate them through an ambulance critical care retrieval ambulance with a paramedic that's on there that will assist them mentor, which is quite important. And then rotate to the different control rooms, so that they can see the information that they're getting in from hospitals to request transfers, they can see the different resources, understand all those limitations and the decision making there. Then a rotation in neonatal ICU or pediatric ICU where appropriate, and preferably do that within the setting that are going to be working in or the location that they're going to be working in. Because that would be quite important there. And then a portfolio builder. So, something that that I've seen works quite well with the students are portfolio-based training. So, they talk about a case that they've transferred and what are the learning points and opportunities within that case, and that is then presented to the rest of the group, so that everyone can learn all the way through. So whatever program there is, needs to be quite an active program, or training program that allows for multiple learning opportunities on different levels continuously and that it's quite interactive with everyone on the group. And then mentorship would be absolutely vital, portfolio based and then rotation between different sectors.

**Researcher 1**

Alright, thank you very much. So just to summarize that section, then. So, you said the approach with this kind of education that should not just be class based, that should be a hybrid approach. So, it should take place the learning in different settings, also bringing in specialists to cover different topics. For example, congenital defects and how they manage that in hospital, then also online content. And then you focused a bit on a portfolio building. So, the student would then work in an ambulance, more specifically critical care retrieval ambulance with those paramedics, also in the control room, and then also neonatal ICU or pediatric ICU. You said, a good method of learning and sharing was when they came across a case they would discuss the case and present it to the rest of the class so that everybody could learn from their experience. You also said that it should be an active program, it should be interactive. And then lastly, mentorship is very important. Is that correct?

**Participant 1**

Yes, that's correct.

**Researcher 1**

Great, thanks. So, moving on to the next question. You did touch on this earlier, but if you had to say what core knowledge and skills these practitioners would graduate with, so we are talking about this additional type of training, whether it be postgraduate, at whatever level, and what core knowledge and skill set Do you think these practitioners should qualify with? It can be just very broad.

**Participant 1**

Sure, so the normal anatomy and physiology, understanding of congenital abnormalities, whether this was taught on the programs I do you feel that would be important to have it as your one of the foundation things to start off with Then from the additional foundations would be really critical care retrieval systems. So, teaching the students you know, this is what dedicated retrieval teams are, this is how we decrease adverse events. These are, what adverse events are, these are adverse events, systems, even I would feel even go as far as doing really good clinical governance and quality assurance training and patient safety training for these students. And I mean, even giving them the tools to help them set those type of things up. Because we're not sure that all the EMS systems out there where they would be going to would have this in place. I do feel it's important to provide the tools and the knowledge for them to take with wherever they go to go and set it up. If it is absent. Then patient safety, EMS management, within specialized services, the anatomy and the physiology, ventilation, specific neonatal ICU care, so you're everything from skincare to pressure care to everything there, then it's feeding and glucose management, temperature management, all those types of management, things infection control, and so forth, then specialize things that I mentioned, ventilation. I think ventilation needs to be completely on standalone or integrated with the rest of the stuff but there needs to be quite a bit of time spent on that. The ventilation should include everything from a bvm to a neo-puff to a blending to using an actual baby pack and when not to use it and how to use your specialized ventilators and nitric oxide and everything specialized under ventilation. And then another section would be specialized care. So, you’re really sick neonates that require extra care. And then under the disease processes, everything that we will be able to see in a neonate, we need to have a really good understanding of what they're going to see and what they're not going to see. Then hemodynamics, sepsis should also be included. And then if I haven't mentioned that, transport specific so aeromedical the issues with aero-medical how you change your care there, ground ambulance based and how you manage those type of things. I must say, I haven't gone through all of that. And I'm sure there's probably about 20 more things that I could add. But I think I'm just going to stop there for now. And then I can always come back to it and come up with some more stuff.

**Researcher 1**

Great, thank you. I appreciate that. Obviously, it’s such a big field that you cannot remember everything. But just to quickly summarize some of the points that you made. You said that anatomy and physiology were to be covered. And whether these guys that covered in the course or not, it will have to be repeated just to make sure that the base knowledge is present. And then critical care retrieval systems and how to decrease adverse events, patient safety and providing them with the tools to ensure that this patient safety and minimizing adverse events, and EMS management. And then you placed a lot of emphasis on ventilation and the importance of that, obviously, that's a big field and quite specialized within neonates. So, there are various methods of ventilation and the air mixtures and everything that you mentioned. Then ICU care, and feeding, temperature, blood sugar management and infection control. All the various diseases that these paramedics might encounter when they see the patients and they need a good understanding of these diseases. And then the hemodynamics and sepsis and how to manage these patients. And then lastly, the modes of transport and the considerations for the various types. Did I cover that?

**Participant 1**

Yes, that's correct.

**Researcher 1**

Okay, perfect. Thanks, then tell me, did you review the pre-reading material on the neonatal transfer data?

**Participant 1**

Yes, I have.

**Researcher 1**

All right. And then when, when looking at this, profiles of the of the neonatal data, do you agree with the types of new notes that were transferred over this period? Did you see similar cases and your current exposure? Do you think this is a good representation of the type of neonates that these specialized units would be seeing?

**Participant 1**

Yes, I do feel that that is what we saw, mostly when I worked on neonatal retrieval. I do think that is what most of the guys are seeing, I do feel, um, I'm not 100% sure if the data was from a private service, or both private and state. And if you guys have that information,

**Researcher 2**

Sure. The data was from a national sample, but only from two national private services.

**Participant 1**

Okay. Thank you. So, the thing that I have seen is that you do see some more complex cases from the state from the state ambulance service. So, I do think it'd be quite important before setting up a training program to also have a look at you know, what, type of cases they see quite often in the state as well, and I'm sure that data is available somewhere. But I do feel it'd be important to see what type of cases they would see in state as well. Because just listening and seeing what they doing in state that always made me realize this, there's so much more going on out there that we don't always know. But yes, I agree. The numbers I see does look like what I saw when I worked.

**Researcher 1**

Okay, great. Thanks. So, if we take into consideration that this study was from national private ambulance services cases, and you said that you would like to see more information on the state's cases. In your opinion, if we had to obtain that information Would there be limitations? What would you expect to see? Or are you just curious and you don't know what to expect?

**Participant 1**

I think I'm just curious to see what type of various different things they also see whether they see similar things in the high volume. So, do they see a lot of the pneumonias that we saw here? And the congenital and the respiratory distress? Do they see that or do they see more of the NECs? Or the sepsis? Do they see more of that? And also, would it be quite interesting to see if, you know, the high acuity but low, low volume things? So, like a really specialized thing? Do they? What type of things do they see? Because that would certainly help and guide, what type of training and how you would prepare, guys within this training program that you would offer? I do feel it needs to be inclusive of cases that you would see in both the private and state sector.

**Researcher 1**

All right, thank you very much, I do we I agree with you. There are obviously limitations to the study. And it would be not just nice, but also it is important to know, from the state side, what type of patients they see and profiles and how they manage these cases. I know you've covered quite a big section on the knowledge and skills that these people will need or have when they qualify. But looking specifically at these cases, like the highest amount of cases was congenital heart defects? Is there anything specific that you think, based on these cases that the guys will need to know?

**Participant 1**

Yes, I feel the basic knowledge, the physiologies would be quite important, especially in the neonatal side, because it's quite specific. And then something that we don't really cover within our EMS sectors is the medication specifically that that's being administered, I do feel that as paramedics, we will take it on, read up about the medication a little bit, just make sure we have a bit of understanding about it. So that if something were to go wrong, we would know what to do. But I do feel it'd be quite important to take the medications that these specialized cases need. And really have a very good understanding of all the pharmacology behind that. So, do you feel that that's something quite important to come up? Should I carry on and talk about the technical things that they would need to know, we want to the next one?

**Researcher 1**

And I think you covered quite a bit of the technical aspects before, unless there's something that you would add additionally to, let's say, the ventilation and the specific needs and understanding of the diseases. And you mentioned now the medications and because the medications that are not on the scopes, they need to understand. Anything else you would add to that?

**Participant 1**

Yeah, so I'm just looking at this. Attachments and or interventions that's on here, which is actually really great. Greatest, it's really nice to see that type of data. So quite important to make sure that most of these aspects are discussed in really good depth detail on a really finicky level, if that makes sense. So, something like CPAP, how do we go about doing that. The humidification, how the devices work, the limitations, the pre-hospital field and so forth. Then line management, select your central lines and you’re a-lines, reading of that and safety and so forth. Even though we do that training and on our PMC program, I do feel it's something that's quite important to go through. And then something that that I learned while working was the real proper use of infusion devices. It just baffles my mind; how technical it can be and importantly is to know and understand the limitations and all the finer details of the devices that you make use of and then there's small little things like there's a colostomy bag and like your NEC kids that sit open all the way through. So, like your infection controls and so forth on getting on a tangent. So, I think that's, that's, that's about it for me.

**Researcher 1**

All right. Thank you. So, you said the infusion device is quite important because it's not just a matter of putting in a syringe and pushing start. There's a lot that's involved in that. And then some of your special need’s cases like your NEC babies, colostomy bags and infection control. And yeah, I guess we can elaborate a lot. But I think the important takeaways, we look at that data, and the type of attachments and it should be quite a bit of emphasis and like you said, finicky details regarding those attachments. Is that correct?

**Participant 1**

Yes, that's correct.

**Researcher 1**

Okay. The next section it covered in the literature review the curricula that was available, and the only local study that I could find was the comparison of the university curricula on neonatal critical care transfer. And it was just critical care retrieval. In general, it was not specific to neonatology, but it was the content would be within them. Do you agree with the information that was presented? That doesn't look in line with your experience?

**Participant 1**

Yes, I have gone through that study before. And yes, I think what we mentioned in the beginning of the session was that the programs are quite limited in time, when it comes to neonatal specific training. Whether that's a lack of the program, or whether it's perhaps just a lack of understanding of what's needed out there, or just really highlights that there really is a need for training of paramedics that will be transporting neonates. I think it highlights a lot of those issues. Then also, when it comes down to the critical care retrieval, I do think it is nice that it is being taught through the different universities in the program. I'm just not 100% sure how much of the neonatal things they do there for that.

**Researcher 1**

Alright, thanks. And then if we look at the International courses that I found. Is that sort of in line with neonatal critical care transfer? The information out there was quite limited. Do you agree with what we found? Or are there any other courses that you would add?

**Participant 1**

No, I agree with what you found there. I do agree there is quite limited programs out there for it. And I do think it's quite difficult taking something from an American training program or European training program, or just, we can't just take things like that, and plug it into our setting. I think what we see and especially for what the study that we saw, they just highlighted that we see things and do things and in our setting that I don't think paramedics in other countries would have to do or be able to do just from my own literature reviews.

**Researcher 1**

Alright, thanks for that. So, you are saying that the international curricula that are available, we cannot just use directly on our Southern African practitioners and the patient population that we see. Is that right?

**Participant 1**

Yeah, that's correct.

**Researcher 1**

Then just tell me if we had to say the method of assessment for this additional training, what would you suggest as a method of assessment in your experience?

**Participant 1**

It's a very difficult question to answer. Assessment becomes so variant and different things that you really need to consider whether it's validated or not validated. If I think if I had it my way, feels quite strong about continuous learning and continuous assessments. And then obviously, because this would be a training program afterward, after degree program. Let's not come from the university, I do think it'd be important to include continuous assessments. It's very difficult to do assessments with from a simulation point of view, I feel lot of the universities use simulations and OSCEs, for assessments, but I do feel, especially in this population, that does make it quite a bit difficult. But I don't think you can just walk away from it, I do think there needs to be some aspects that you would have to assess in a simulation environment that you can't really do safely, anywhere else. Others assessments could be well validated, written assessments. And when I say continuous assessments, I'll bring up the portfolio again, and making use of well validated tools to be used by the mentors, that the students would be working with, I think an interesting one was, they had a validated tool that looked at the students working with the lecturers and they could use that validation, validated tool to assess their clinical practice. I think a tool like that would be great, especially in the setting would have to change a little bit. But I do think that would be quite useful. Because you, you could see, you could you could assess so much from a student while doing a transfer. And that would be it would be sad to not include that into some form of assessment if you have a mentorship program. So just for me, I feel continuous assessments, validated tools to be used for work integrated learning assessment. Unfortunately, I do feel that some form of simulation and or OSCEs or simulations or oral should be included in some way or form in their assessments as well.

**Researcher 1**

All right, thank you. So just let me summarize that. So, you said, assessment tools need to be validated, that's quite important. And then you mentioned continuous learning and assessment. You said that simulations and OSCEs do have a place because especially when you consider the type of patient and patient safety, but it shouldn't be the only method of assessment, written assessments, portfolio of evidence, including mentorship, and then work integrated learning, and then orals also would be a method of assessment. Is that correct?

**Participant 1**

Yes, that's correct.

**Researcher 1**

Okay, great. Thanks. The last question from us then would be: If you could give the most important thing that the curriculum committee while they develop a new curriculum, what would that message be?

**Participant 1**

I think it'd be important to, before writing any of the curriculum to really understand what the need is, and how this person would be integrated into the systems and how continuous learning would take place after this curriculum. I think those are important questions to ask before embarking on this type of curriculum build is to have that understanding of what's going to happen to these guys afterwards, so that it's not just the curriculum that's put out. Here's the training, go and do the training and then fight for yourself. I do feel that those questions need to be asked before. And then secondly, to have a mentioned before but have a good interactive training program and not just a sit in class-based training program that really needs to be interactive and involved program, because that's the students of today will learn a lot more in that regard than the normal way of learning.

**Researcher 1**

Okay, perfect. Thank you. So just to quickly summarize, as we need to understand the need for this type of training, it needs to understand how this type of qualification will be integrated within our systems. And then how will continuous learning continue after this type of training? And then you said this type of program needs to be a good interactive program with a hybrid approach? So obviously, various methods. Is that correct?

**Participant 1**

That's correct. And I just want to add on as well, that it needs to be quite inclusive. And I think, well, I mentioned earlier, how do we include practitioners with different various qualifications to be included in a program like this? Which does make it complicated when you have it in a university? But sure, sitting down and figuring that out, that would work.

**Researcher 1**

Thank you. I do agree, I think it also needs to be inclusive. But there are challenges to including everybody with the history and the qualifications. And that's all from my side.

**Participant 1**

Absolutely. It's I think it's quite exciting what you're embarking on. So quite looking forward to see what you guys come up with.

**Researcher 1**

Okay. I'm going to stop the recording now.
