# Supplementary material for: Developing a South African curriculum for education in neonatal critical care retrieval: An initial exploration
Source: PLoS One. 2023 Aug 31;18(8):e0290972. doi: 10.1371/journal.pone.0290972 (PMC10470938; doi:10.1371/journal.pone.0290972)
Supplement: S1 Data — (ZIP) [file pone.0290972.s002.zip › Data Compressed/Discussion schedule Appendix A Student Group[4].docx]

**Appendix A**

Discussion schedule for:

**INITIATING THE DEVELOPMENT OF A SOUTH AFRICAN CURRICULUM FOR EDUCATION IN NEONATAL CRITICAL CARE TRANSFERS**

*FACILITATION*

*Before the interview/discussion, please refer back to these notes to ensure familiarity with the content. All interviews/discussions should be run by a facilitator. The facilitator is to lead the discussion while taking notes and operates the recording equipment. Each discussion should lead to some conclusions.*

*PREPARATION*

*Test the recording equipment and its sensitivity. The participant consent forms should also be available and ready. Offer the participants refreshment. Make sure that participants are comfortable before the start of the discussion. Ensure that the participants have signed the consent form and that she/he consents to being audio recorded.*

*Read out the statement on confidentiality:*

**There are no right or wrong opinions to any of the topics of discussion. I am here to establish your individual views. Any opinions expressed will be treated in confidence.**

*INTRODUCTION TO THE SESSION*

*Briefly introduce the session. Start by introducing yourself. You may start the session by:*

**I would firstly like to thank you for sparing the time to come and talk about initiating the development of a South African curriculum for education in neonatal critical care transfers. Loosely, the transfer of neonates in South Africa is performed by advanced life support (ALS) providers. This high risk service is reserved for specialist teams internationally. Adverse events during these transfers have been associated with the providers’ level of knowledge. There is currently no specific course in neonatal critical care transfers offered in South Africa. The practitioners that fall under ALS providers have variable education backgrounds. There is no guidance from South African governing bodies on the methods and content of education in this specialised field. The purpose of this focus group discussion is to establish your opinion on education in neonatal critical care transfers in South Africa. There are no right or wrong opinions, I would like you to feel comfortable saying what you really think and how you really feel.**

*FOCUS GROUP DISCUSSION WITH STUDENTS:*

**Please could you start by introducing yourselves and giving a bit of career background.**

| *PROBES AND PROMPTS* |
| --- |
| Demographic data: Qualification, Location, Position |
| Private versus provincial  Neonatal transfer experience |

**Understanding the current limitations to training in neonatal critical care transfers within academic and health institutions in South Africa?**

| *PROBES AND PROMPTS* |
| --- |
| - How much time was spent on the topic of neonatal transfers during your training at university or college? - In your opinion, did this training adequately prepare you for the neonatal transfers that you are currently performing? - What strategies or mechanisms did you adopt to improve your knowledge and skills in neonatal critical care transfers since your basic qualification? - Do you have any recommendations for the current neonatal transfer curricula presented by Universities and colleges? |

**What would you say your specific needs are in learning and assessment (TLA) as a student in neonatal critical care transfer education?**

| *PROBES AND PROMPTS* |
| --- |
| - Do you need additional training in neonatal critical care transfers? - If so, educating in which specific areas of knowledge, clinical and non-clinical skills, do you consider important to enhance your competency in neonatal critical care transfers? - In your opinion, how much time should be dedicated to such education, and if implemented, how much time will you be able to dedicate to it? - What format should this education be presented in, i.e.: would a short course be sufficient, or online or pgdip..? - Within the curriculum, which teaching methods should be included e.g.: workplace attachments; simulation; lectures etc. - Would you consider financial support to pay for additional training an important consideration? - Would you consider travel to another city for such training a potential limitation? - Do you have access to a computer and an internet connection? - Do you have suggestions to ensure that this education/curriculum is inclusive, permits diversity, and equity? |

**To derive the goals of a curriculum in prehospital practitioner training in critical care transfers of neonates?**

| *PROBES AND PROMPTS* |
| --- |
| - Experts in the field of neonatal care and critical care transfers have suggested the following core topics to be included in a new neonatal critical care course. - **Please refer to the background reading document** - Experts have suggested the following methods and duration of education and assessment for this course: - **Please refer to the background reading document** - Do you agree with the suggested format, methods, duration, and assessment for the curriculum as suggested by these experts? - Is there any component/factor/topic that you consider significant which has not been mentioned by the experts? - What do you consider a reasonable cost of such training? - Would you travel for such training? |

Final Reflection

- What is the most important thing you would like to tell the curriculum committee as they work towards developing a curriculum in neonatal critical care transfers?
