# Supplementary material for: Developing a South African curriculum for education in neonatal critical care retrieval: An initial exploration
Source: PLoS One. 2023 Aug 31;18(8):e0290972. doi: 10.1371/journal.pone.0290972 (PMC10470938; doi:10.1371/journal.pone.0290972)
Supplement: S1 Data — (ZIP) [file pone.0290972.s002.zip › Data Compressed/Transcript 6.docx]

Interview 6

**Researcher 1**

Good morning. Thanks for joining me. Just to confirm on the recording that you okay with me recording the session just to give consent?

**Participant 6**

Yes, No, I'm fine with you recording it.

**Researcher 1**

Okay, thanks. So, just to confirm that this interview is voluntary, you can withdraw at any time. And then if there's any personal information that comes out during the discussion, I will remove it during the transcription. Are you happy with it?

**Participant 6**

No, I'm happy with it.

**Researcher 1**

So, just a quick background to the study. As you know, advanced life support paramedics, conduct, these critical care transfers that we're going to discuss today. And every ALS that I've spoken to before said that when they started doing this, they were not prepared for this. And they had to close that knowledge gap themselves. You know that these transfers are high risk in nature. And we've also seen that the adverse events that happen are linked to the level of knowledge of the provider. So that's what our discussion is going to be around today. So, to start off, can you just tell us about your background? workwise, especially neonatal critical care transfers, and your education? And how long have you been doing it? If you can give us an idea of that, please?

**Participant 6**

I'm a critical care assistant (CCA) paramedic that started doing ICU transfers in 2017. And very much on the neonatal side. We started with a new state hospital that opened, trying to provide them that service for transporting patients into the hospital and also out of the hospital. Yes, it was a great struggle, especially with the newborns and we do not have that knowledge that was given to us on the course that we did on these little infants, little neonatal babies, you transport these patients, and you're most likely like, most ALS say the same thing was that we had to get this knowledge ourselves and start preparing ourselves for these little ones but went better when transporting them. Currently, I'm still doing them quite a lot. It started picking up after this COVID that we had, and we starting to do more neonatal transfers again.

**Researcher 1**

So, you said you started doing the transfers in 2017? When did you qualify as a CCA?

**Participant 6**

Qualified as a CCA in 2008. And ever since then, was on an operational paramedic. Since 2017, when I went over to state, and started working on an ICU vehicle.

**Researcher 1**

So, tell me, how did you close these knowledge gaps? How did you figure out how to manage a neonate properly? Can you just explain that to us?

**Participant 6**

So, something that was very fortunate for me when I started at state ICU was that we had a lot of your emergency medicine registered students. They started working with us on the vehicle itself, and they had like a two-month pre-hospital block that they had to do. And what was great that I always had these doctors with me when transporting some of these patients, and I could get the information and the knowledge from them on what to do better. And what was very nice is that at the state hospital there was quite a great doctor as well, that every time we went there with a patient, we could get some knowledge from her and especially on things that we did not know about. And then also went into the local things like books and Google and all that stuff to go in and educate ourselves better. And by transporting these patients safely.

**Researcher 1**

So, what I'm hearing is that you explaining like a mentorship system. So, at the state hospital you had some specialists that you could consult with. And then also you had registrar doctors working with you sometimes and you could also discuss it with them. Do you think the doctors that worked with you? Their shifts? Were they knowledgeable with neonatal transfers? Or was it also new to them?

**Participant 6**

No, some of them, just before they started the practical block with us was actually beneficial because they did like a three-month block at state neonatal ICU as well. And basically, when we had a difficult patient, that we could then phone colleagues and peers that we can discuss this call with, before we transport the patient. And you know, a lot of the medications that they use, we don't have those type of medication on our scope. So, like I say, EM guidance, Google, all these was our very best friends at that stage.

**Researcher 1**

So, for example, in the UK, they use a multidisciplinary team where you would have ICU sisters or doctors and paramedics mixed team, do you think this is a good approach? Because you explaining you and the doctor with different scope from the other person on the vehicle? Do you think that is something that would work?

**Participant 6**

It would definitely work. At this stage, we looking at our staff availability in our country. And we do have a bit of a problem with staff availability. And so, if we can look at it at effect that there are no problems with staff available, if it is something that will work quite well. Because if you've got those different disciplinaries with different scope of practice, it would work great at the end of the day. And we can all then put everything together and do the transfer to the best of the ability of the team. And we also do know that the Americans love the team approach to everything. If we look at just all the American Heart Association courses like ACLS, PALS and all those things that they love the team approach. And that is something that I think we in, in South Africa, we need to take that more into consideration and work as a team.

**Researcher 1**

So, what I'm hearing is, if we had the resources, it definitely would be a best practice approach.

**Participant 6**

Definitely, yes, it would be beneficial to the patient itself.

**Researcher 1**

Tell me, with you being stationed at a university. Do you work with students as well, from the program, the BEMC program?

**Participant 6**

Yes, we do. We've got the practical block for the third years and the fourth years are starting very soon now in April, and they will be rolling out and the students to work with us every so often we've got the third years. And the third years did not have a lot of natural experience here yet to have neonatal knowledge at that stage. So, it's a bit of a difficult but it's also beneficial to me, because I am then turning myself into a trainer then and starting to educate them on the little knowledge that I've got. The fourth years are quite a bit of a better group of people working with the neonates because they already have a little bit of a neonatal knowledge that they also bring to it and challenging our education and our knowledge at the end of the day. And that makes it far better you know, being in the training environment with students and myself being the basis on my toes as well. Educate the students and it's actually great for me.

**Researcher 1**

So, what I'm hearing is you said that third years don't really have much neonate exposure, but fourth years do. How much time would you say the fourth years will be spending on the critical care vehicle like yours?

**Participant 6**

Well, at this stage, I think they do 12-hour shifts. The fourth years on my vehicle, and then there's a couple of shifts that they do on a vehicle at some of the private services as well. So, to me, the amount of time that these guys do have on an ICU vehicle, doing ICU transfers, is very minimal. And the fact is that they might be doing 48 hours on an ICU vehicle. But the problem is, they're not doing constantly 48 hours of neonatal transfers, though, or even ICU transfers as such. The daytime is very much limited. By the time that they qualify, they might have seen one neonatal transfer, it could have been a simple transfer of a little one that might have a bronco pneumonia. And they haven't actually seen the other diseases and so on. That is out there with these little ones, especially on the ones that we do the most is the congenital heart diseases.

**Researcher 1**

All right, so what you saying is, it would be great if they had more time and more exposure to patients. But time is limited. So, with that in mind, what would you say would be the solution? Would we have to drop something else within that program or additional training after they qualify?

**Participant 6**

I'm not saying that they need to drop anything in that program. That program is quite a vast great program at this moment. And the amount of knowledge that they gained and the little bit of experience on an operational side and so on is great. But they're going to have to add something to the qualification postgraduate, like, you know, a little bit of more intense neonatal transfers, or it's critical care retrieval transfers afterwards. So, a post-grad type of course, would be great for these guys, because I can just get more comfortable with transporting these little ones.

**Researcher 1**

Tell me, the BEMC guys that qualify, the new graduates, are they ready to conduct neonatal transfer by themselves? Or do you think they should be transferring different levels of patients or work with mentors? Or what do you think would be the solution?

**Participant 6**

My opinion is that they are totally not ready. My experience that I've had with some of the newly qualified BEMC paramedics that came onto the road and started working with state on an ICU vehicle, is what we've seen is that they are thrown into the deep end, and they need to transport the little one for the first time by themselves. And what's really great about them is that they then start phoning us for help, you know, they're not very comfortable in doing it, that they will then phone either me or phone one of the other ALS paramedics at the bases and ask for help that we can assist them in transporting these little ones safely. And I personally think that any one that once he is qualified, any starts transferring these neonatal patients should actually have somebody to mentor them and guide them through the time as a neonatal ICU transfer paramedic.

**Researcher 1**

Yeah, I agree. That sounds like a good solution, mentorship, but I think the gap in knowledge would still be there. And you did touch on that earlier with saying additional education. You mentioned postgraduate education, what length of education would you suggest and also what method If we look at in class, is its full time or part time, online, distance? How do you see that type of education rolling out?

**Participant 6**

That type of education is at this moment, I would say that there needs to be contact time with the student itself that's going to do this postgraduate course. And it could also be online. I'm all for online studying, especially if the student has self-discipline to do these things. But there needs to be contact done with patients. That's all good and well, that you've got all the knowledge that you've taken in, but there was no actual contact time. So, in that course I would put, you know, practical time in a neonatal ICU, and also on a neonatal ICU transfer vehicle, so that they can get this practical exposure, and see that as well. From a transfer point of view, and, you know, if we look at the time frame for this course, I would suggest this could be anything from six months to a year that you need to look at these courses to, or the length of the course, shall I say it like that?

**Researcher 1**

For who should this type of training be? Should it only be for ECPs? In other disciplines? What do you think the target audience should be for this education?

**Participant 6**

The target audience for this education should definitely be all ALS paramedics. If we look at anybody that is a critical care assistant (CCA), we have some of your diploma graduates that also do a little bit of transfers. Not as intensely as you know, neonatal but they do other transfers. From critical care point of view, your ECP is also those guys do, you know that get onto an ICU vehicle. And ECPs, CCAs and your diploma graduate should be able to do this postgraduate course. And on the other hand, you always have a colleague that has to work with you on this vehicle. And usually, it is an intermediate class or practitioner that they place with you. And in some cases, you've got a basic life support practitioner that works with you. But I'm just saying that it should be something for those colleagues and those crew members that works with you on the vehicle to also do understand the nature of a neonatal transfer. Because at the end of the day, how we working in South Africa at this moment, if I've got someone that is just my driver, they can just as well give me an Uber driver. Because if he doesn't understand these little things, you basically on your own, and I would say that it would be nice for this postgraduate course is an intermediate life support practitioner decides that he is working on an ICU vehicle. And he's very interested in transfers and so on that course should be available to him to do as a postgraduate and specialize a as a crew member with the ALS paramedic, working on this ICU vehicle.

**Researcher 1**

So, what I'm hearing is different levels of education or different exit levels. So, your assistant would not necessarily have to have the in-depth knowledge to conduct the critical care transfer like someone like you, but they will have to have a base knowledge to be a good assistant. So how would we approach that? Would it be different lengths and then at certain stages to people complete with a different level of qualification? Or must everybody finished with the same exit Level qualification?

**Participant 6**

No, I think for them, it could be a smaller, I would say not as lengthy course.

**Researcher 1**

Tell me, when we look at governing bodies and employers? Do you see their involvement as barriers? Or do we need a buy in for this to work? How do you think that should work?

**Participant 6**

All your role players in the health sector, especially in the pre-hospital sector. And the role players like your companies that these guys work for, should buy into it, and should also assist these guys in getting to that knowledge so that they know that if they have a practitioner on a vehicle that is transporting neonatal patients, that they know that we are sending out the best team that there is from our company. So that they also know that they would be correct persons on those vehicles, and not just throw anybody on a vehicle and you got to do a neonatal transfer. And that is actually how it's happening now. I mean, you get employed by let's for instance the state sector, and you get thrown onto an ICU vehicle and do any calls. I'm talking about from adults, to little infants to neonatal transfers, and you have to just go out there and do what you can. At this stage with the little bit of knowledge that you've got going forward, the more you do it, the more you get comfortable for it, the more you start reading books, reading, and then going over and speaking to your peers and speaking to any of your colleagues about it and getting more comfortable with it, you start relaxing on these calls. But the very first time, any practitioner that has not worked on an ICU for the time and now he gets thrown on to an ICU vehicle and he sees these patients with over five infusions up and his getting ventilated, and it’s a really sick critically ill patient that you need to transport. They do at the end of the day, get to that point where they say Wow, wait, I need to phone someone now. That knowledge that you need. It's really, really important for us to know.

**Researcher 1**

Did you see the document with a neonatal data from the study?

**Participant 6**

Yes, I did see that document. I went through it. And I would agree a lot with what was said, especially on the cases that's put on the about what are the most patients that we see? And especially on the congenital heart diseases, we have seen so many of those as well. And I do agree with exactly on the study. From a state point of view, I also see the same, looking at the document. On the top, let's say congenital heart diseases, and then we look at the medication that's also put on. I mean Prostin how many of our ALS paramedics have ever seen Prostin and what does Prostin do? So, I love the study and I think those are the things that we need to target in a postgraduate course. We as a practitioner should know more about congenital heart diseases, bronchopneumonia of these patients, especially on neonates.

**Researcher 1**

So that's, that's good to hear because you are working in the in the state sector. And this study was a national sample of critical care transfers of the two biggest private companies. And then we extracted the neonatal population so that we can use their data for my study. And so, it's good to hear that you feel that it's, close to what you see as well. So, on that, if this is the type of patients that we see, can we put some broad categories for what the curriculum content should be when we look at, what should we be teaching these guys about?

**Participant 6**

Oh, yes, definitely, we can do a very broad-spectrum categories on what we need to do. Like, if we look at what the most is, and what the least of these diseases, and the disease progresses that we see on the study that's done, I think it's going to be a great way of using those things. And let's focus more, especially on let's say, the congenital problems, and the birth defects that there is, then some of the other little stuff that we can also put it in there. But I mean, focusing more on the critical things that we see on a daily basis would be great. So, the study that has been done would be if we can use that, to actually put together the curriculum of this course, it would be awesome.

**Researcher 1**

So, when we look at this document, and the most cases were congenital heart defects, and then respiratory distress syndrome, and then prematurity, and then NEC babies, so those were the most patients. So, what you're saying is the weighting of the course, should be quite focused in that area, because these are the most critical care transfer patients that we see.

**Participant 6**

Definitely. The curriculum should be weighted on those specific studies and the cases that we've seen. We should also not forget about the other, maybe one or two transfers that we do that does not have those things on board.

**Researcher 1**

Then under medication, you mentioned Prostin. So, this is not on the scope of any pre-hospital personnel. But it is used quite often with these transfers. And you said that most paramedics don't really know what the side effects are, and so on. So, she can we say then that these type of medications should be included?

**Participant 6**

I wouldn't say that these medications should be included on the scope of practice of these practitioners. But what I do say is that it should be in the course, so that it can be covered. I mean, when you get to the hospital, when you're going to transport this patient, you should know, what is the adverse effect? What are the contraindications? And what is the indication for this drug? And why are they using this drug? A little bit more in depth knowledge on the drug? If we just look at the fact that the first time that I've actually came across that drug, I had a 15 to 20-minute discussion with the transferring, Doctor, just to find out what the drug does before I just leave with this patient and something goes wrong. And I don't know, why is it happening? And it's actually the specific drug that is used, or the patient that is doing the effect on the patient, and so these things definitely should be included onto the curriculum of this course that they take as that knowledge of these drugs.

**Researcher 1**

Thanks. So, what I'm hearing is we need to teach the paramedics that are going to do these transfers, what the drugs are, how they work. But it will not be necessary to include them in the scope of practice just as a continuation of care, they will then understand what they are administering.

**Participant 6**

That is correct.

**Researcher 1**

On ventilation of neonates, are there special considerations that should be included? Because it's quite different to an adult. Do you agree?

**Participant 6**

I do agree. It's quite different. I mean, if we look at just the pressures and the volumes that we are getting on these little babies, and patients, is that it's a good idea to in the curriculum, add those little things on for these little patients. And I agree 100%.

**Researcher 1**

In the state sector. Do you continue with CPAP on neonates? Do you use heated vent, humidified circuits or not?

**Participant 6**

We actually use it more often. At this stage, now that we've got the ventilators that can do the CPAP for the neonates. It's much better, the hospitals are actually now also finding it more comfortable for us to transport and not do invasive maneuvers like endotracheal intubation, where they know that this is not what that little one needs. So now they know that we are there and we are capable of humidified heated circuits and also on CPAP. So, they they've got this relaxation in them that says, okay, cool, we've got these vehicles. When we phoned for this transfer, we'll ask that they send someone that are comfortable. It really made life very simple. And the other thing that I have to say about that is that at the end of the day, we need also that little bit of a training on the type of ventilator that you're using, if it is capable of doing that. Because what I've also seeing is that you've got your newly qualified person that comes out and he's got no idea how the CPAP actually really works for these little babies.

**Researcher 1**

So, what you're saying is we should be including the special modes of ventilation for neonates? But do you think this is the norm for state to have this equipment? Or is it isolated to your unit?

**Participant 6**

No, no, at this stage, it's not just isolated to my unit itself. It is the norm. Well, the state started to realize that we need these specialized ventilators and equipment. And what's nice is that they started to realize that lets purchase them. I mean, we can't use a ventilator that hasn't got CPAP on these little ones. If the patient, where you are going, is on CPAP. That makes it difficult for the hospital as well when you get there and tell them I can’t do CPAP then they need to then go and do invasive maneuvers. And make it comfortable so that you can transport the patient. So, what we've seen is that, from a state point of view, they have also realized that this is what we need. So please give this to us. And it's becoming a norm in the state side as well. It's not just isolated to us specifically.

**Researcher 1**

That's good to hear. So, on your patient monitoring side, any special inclusions that you would do, would we include ABG blood gases, or special types of monitoring for neonates?

**Participant 6**

Well, at this stage, what we are doing is we've got your Capnography for the little ones. We've got a lot of these little things that we do. We would like to see the arterial blood gases (ABG), and especially when we do the transfers, that's very long. It is long, more than an hour drive, you know, some of our transfers. I mean, you do an arterial blood gas at the hospital and you've got a 15 to 20-minute drive, and then you can do the next arterial blood gas. We do see a bit of an issue when we are transporting long distance, we do see the need to do an arterial blood gas (ABG) on this little one that our ventilation strategies, and the strategies that we are using on these patients are actually working. And it's keeping the patient in a safe manner, in a stable condition as well.

**Researcher 1**

So, what you're saying is we should be including ABG, for in case you do a long-distance transfer?

**Participant 6**

Definitely, it would be great if we can include those little things also on that course.

**Researcher 1**

What method of capnography do you use? Do you use mainstream or side stream side stream? What type of machines do you have?

**Participant 1**

Our machines that we are using are mainstream. And then the vehicle that I'm using at the state is the mainstream, it also has the capnography on the ventilator. I think other vehicles now also have mainstream capnography.

**Researcher 1**

So, we touched on the medications you mentioned. Some patients might have five infusion pumps, some of these transfers get quite complex. On infusions, anything special that we should be including in that?

**Participant 6**

Infusions, there is potassium free Neonatally, that we use as maintenance fluid. And there are some other drugs that we infuse, one of them being Prostin, Dormicum, Morphine. But then you get something like Dobutamine, which was never covered on my course, but they do touch on it in the BEMC program. So those guys do know a little bit of it. But there are so many of these infusions, and drugs and especially if you look at the list of drugs that the study put on, and that is looked at, and there is a lot of them that we have not seen. And we don't know what is the dosages even for these things, including these dosages. On the curriculum for the school it would be actually so great in helping the practitioner at the end of the day to understand the drug and how much the doctor is giving and why the doctor is giving.

**Researcher 1**

Tell me, the curriculum that I included after the neonatal data. Did you have a look at those? There was a local study. So, it compared the curriculum for the universities. And then I included some international courses that were available. I don't know if you got to that section?

**Participant 6**

I actually love some of the things that was mentioned in that curriculum, basically the drugs, the special transport interventions and stuff that needs to be done on there.

**Researcher 1**

So, would you say that some of the content from those international courses could be used when we develop this curriculum?

**Participant 6**

Definitely it can be used. There's quite a lot of them that we can look at, that we can include into the curriculum of the course that you're looking to develop.

**Researcher 1**

Would you say that taking an international course and using it as is in the South African context, is that a good idea or not?

**Participant 6**

We need to know that international course that is there and it's available, and just bring it across this side is that course going to be relevant to the statistics that you see? And would that give the practitioner sufficient knowledge, and information to be able to transport these little neonate patients in our setting? At the end of the day, if we can look at those courses, and just adapting our course, accordingly to what we do in South Africa, then I think it could work.

**Researcher 1**

So, what you saying is we can take some of the content to make it relevant to our context? If we look at assessment during this course. What worked? What do you think works well, for this type of education, looking at the students that you've seen, the doctors and discussions that you've had? What do you think is a valuable type of assessment to gain someone's knowledge? To see that they're competent in this field.

**Participant 6**

If we look at clinical skills that the practitioner has done, while he was doing his critical care assistants, diploma course, or even the BEMC program, they were found competent in a lot of the skills there. I'm always saying, theoretically, you need to put that down on paper and see if that person understands the number of drugs, the ventilation strategies on these little ones. And though the theoretical written paper needs to be done. Then from a practical point of view, a patient stimulation where he basically gets even a patient, we need to actually transport this patient from one ventilator on to his transport ventilator and transport equipment. Getting him across to see, that he understands how to do these things, and also what the drug that he is using. Like the Dormicum. Why is he putting it across over to the patient simulation at the end of the day, is the way forward to see if this person has knowledge on transporting neonates and is he competent in doing it. And it goes about the curriculum of this course, on how long this course is going to be. Literally since it's a year course. And then there should be at least not just a final, practical simulation, or a final written paper. But it should be a continuous assessment on the on the student during the timeframe of this year. That's where contact time with the student comes in. Not just an online program. I mean, complete everything at home. But there still needs to be that contact and to see that the student understands from a practical point of view what he needs to do. It's all good and well to see everything online and see something somebody's showing you how to do it on a video, but you doing it yourself at the end of the day, I would say that is the power of the assessment that is very important on these neonates.

**Researcher 1**

So, you saying that there is a place for written theory assessments just to test the base knowledge, but then there should be quite an emphasis on the practical side. So, you mentioned patient simulations, and you mentioned continuous evaluations throughout the year. I just want to touch on the patient simulations. Are we talking about a doll in a lab or are we talking about a real patient with supervision? How do you see that being assessed?

**Participant 6**

That assessment can be done either on a doll in a lab. But I would say that contact time with a real patient with a mentor or assessor on board with you on the vehicle on way to hospital, or whatever the case may be, would be actually quite great. I mean, just you mentioning that would be also a great idea of assessing that person. Having someone standing over your shoulder and looking at what he's doing. And you can also correct him if he's going wrong some way. Yeah, I agree with that one as well.

**Researcher 1**

Because if we're looking at working with real patients, there's also risk involved. So, would we agree to say that there should be supervision then from a senior?

**Participant 6**

Yes, we agree, there should definitely be supervision from a senior personnel.

**Researcher 1**

Do you see value in oral examinations as well? Should it be standalone? Can oral exams be brought into a practical assessment as well? For probing into the knowledge and the understanding?

**Participant 6**

Your oral exam is always a good exam. Because you can also get the knowledge and assess his knowledge on what he's doing. But you know, for me this is very personal, you know, putting something on paper, and talking about it is sometimes are two different things. So, if you are adding an oral exam as well, it would be great because, you can always cram a lot of stuff into your brain and put it down on paper. But sometimes it's very difficult to cram stuff in your brain and talk about it. Yes, an oral exam may be also a standalone part from the theoretical and the practical part.

**Researcher 1**

Then skills assessment, because there are various skills involved with this, this type of education, would they be assessed as you go along in hospital and signed off? Or do you have the old traditional OSCE stations? Or how do you see that being assessed?

**Participant 6**

On this postgraduate course I wouldn’t bring in an OSCE station. I mean, this practitioner should be actually very much knowledgeable on using say, an infusion pump or a syringe driver. And what I'm saying is, this could be something that you sign off in hospital, get it done in hospital, or on the vehicle that you're working with someone on an ICU vehicle. I think that would be a patient with him, we know that this person has been signed off on using a syringe driver, for instance.

**Researcher 1**

Then special considerations for transport, packaging the patient. The modes of transport. Do you agree that not everybody knows everything about the different modes or how to package a neonate for an ambulance properly?

**Participant 6**

I can say its very much agreement on that. The different modes of transportation need to be included into this course. Packaging of these little ones is very important. And just on a personal thing as well, what I've seen is that you've got a six-month-old baby. Putting him into an incubator. He doesn't fit into that incubator, but you want to keep him in a safe environment. Now you put him on a bed. The bed is so big that you can actually strap this patient down. So, if we consider writing how are we going to package this patient so that the patient is safe, you know the circuits that you're using is not dangling everywhere around. A little bit of packaging these little ones especially as six months, even up to a year doesn't fit on these big adult stretchers. You got maybe one securing strap on this patient on the bed itself to secure this little six-month-old there must be packaging of these little ones should be included into this course. And that is of vital importance for these little ones.

**Researcher 1**

Do you think we should include anything on systems? How ambulances are dispatched and the criteria? Should people understand how systems work within critical care retrieval? Or would that not be necessary?

**Participant 6**

You've got someone that's both a graduate, and he's already been working in emergency medicine field or emergency department. It should be able to know how the system works. But I mean, including those that they do know how it works from the call gets taken in, and how the call gets screened, and how the call then gets dispatched to which vehicles and so on. So, I think, if you include a little bit of a basic understanding on that would be beneficial for the student as well, and to the practitioner.

**Researcher 1**

We mentioned earlier that we're looking at it to be inclusive, so not only pre-hospital people, but also in hospital people that could potentially join these types of teams. And an ICU sister or a doctor might not have a lot of knowledge in that field. So, we should be including some of that information.

**Participant 6**

Definitely, in that sense, you know, including the information for working as teams, doctors and nurses, it should be a basic understanding of how this system works.

**Researcher 1**

So, a final thought for the team that's going to work on this curriculum design? Any final thoughts, the most important things that they should focus on or some advice?

**Participant 6**

The one part and we've not touched is patient safety. Now you've got this big ambulance that you put in a little person. And, anything can happen on the road, and packaging this patient for the safety of the patient. Is your patient secured in the back of the ambulance? But if something happens, God forbid, whatever happens, your patient at the end, and your practitioner is safe. And you're in the back of the ambulance. I mean, you've basically touched on a very sensitive point for myself, because I've seen so many of these guys transporting patients. That is not even strapped down in the vehicle. The equipment is just placed on this big stretcher, and things are not working as what it should. And if something happens, and your driver tries to avoid an accident and slams on breaks. What's going to happen to the patient is going to just fly through the air in the ambulance. We're looking at this curriculum, that is something that really, really needs to be looked at. And from a state point of view, I've seen that securing patients in the back and packaging these patients correctly is a big problem in the state sector. I have not seen from a private sector how they strap the little ones down. And then you know, the medication that there are all these little ones. I need to look at all the medication that get used. And we need that information as practitioners, postgraduate to know that these medications exist. And these are the type of medications for the type of diseases that's out there and set up the curriculum so that we know these medications much better at the end of the day.

**Researcher 1**

Thank you. So yes, I agree patient safety, because those considerations are not the same for when you're in a neonatal ICU. There's a lot to consider when you're in a moving vehicle. And then yes, like we touched on it earlier, the medications the knowledge of it and the safe usage, whatever what to do if that goes wrong, should be included. So, thank you. If there's nothing else you want to add, I'm going to stop the recording.

**Participant 6**

Thanks very much.
