# Supplementary material for: Developing a South African curriculum for education in neonatal critical care retrieval: An initial exploration
Source: PLoS One. 2023 Aug 31;18(8):e0290972. doi: 10.1371/journal.pone.0290972 (PMC10470938; doi:10.1371/journal.pone.0290972)
Supplement: S1 Data — (ZIP) [file pone.0290972.s002.zip › Data Compressed/Invitation letter WW.docx]

**Dear Colleague**

I, West Williams, the primary researcher, am currently doing my master’s in philosophy: Emergency Medical Care at the University of Cape Town. I am conducting research with the aim of initiating the development of a curriculum for education in neonatal critical care transfers. I am approaching experts in South Africa to participate in one-on-one online interviews.

**Study details:**

**Title:**

INITIATING THE DEVELOPMENT OF A SOUTH AFRICAN CURRICULUM FOR EDUCATION IN NEONATAL CRITICAL CARE TRANSFERS

**Research Team:**

Primary Researcher: West Williams

Research Supervisor: Dr Willem Stassen

Research Co-supervisors: Elzarie Theron and Dr Waseela Khan

UCT HREC clearance received with reference number **474/2020**

You have been purposively selected to participate in this study as an expert in the field. Your perception regarding education in neonatal critical care transfers in South Africa will be explored during the interview. Being part of this research is entirely your choice and voluntary. Whether you decide to participate in the research or not – there will be no consequences to you. You may also decide to change your mind and may withdraw from participating in the research, even if you agreed to it at an earlier stage.

If you are interested to participate, the following process will follow:

- Agree on appointment date and time.
- Sharing of the background reading document that will inform the interview.
- Obtaining your consent to participate in an audio recorded interview so that it can be transcribed at a later stage.
- The interview should take approximately one hour.

If you are interested in participating in the study, you may contact myself, West Williams on my mobile number: +2765 903 9271 or email address [WLLWES004@myuct.ac.za](mailto:WLLWES004@myuct.ac.za)
