# Supplementary material for: Developing a South African curriculum for education in neonatal critical care retrieval: An initial exploration
Source: PLoS One. 2023 Aug 31;18(8):e0290972. doi: 10.1371/journal.pone.0290972 (PMC10470938; doi:10.1371/journal.pone.0290972)
Supplement: S1 Data — (ZIP) [file pone.0290972.s002.zip › Data Compressed/Transcript 4.docx]

**Interview 4**

**Researcher 1**

All right, we are recording now.

**Participant 4**

Excellent.

**Researcher 1**

Just for the record now that we are recording. I just want to confirm that you give consent for me recording this.

**Participant 4**

Absolutely.

**Researcher 1**

Thank you. You received the consent document because you sent it back signed to me already. So just to quickly summarize the consent part of this. So, the interview is voluntary and you can withdraw at any time. And then if there's any personal information that comes out of this, it will be anonymized during the transcription phase. Are you okay with it?

**Participant 4**

Okay.

**Researcher 1**

So, just a quick background to the study. So, the transfer of neonates in South Africa is performed by advanced life support providers. And it's a high-risk service. And adverse events have been linked to the providers level of knowledge and South African ALS, providers have variable education backgrounds. And then there's no guidance from South African governing bodies on the method and content of education in this specialized field. So that's just a brief background to the study. So, to just start, can you please tell me about your background, your education, and specifically neonatal critical care transfers and your experience in that field?

**Participant 4**

Okay, so I'm a qualified registered nurse with a degree in occupational health and experience within the ICU, the emergency care divisions and theater, in hospital and general surgery. I'm also qualified as a critical care assistant through the government services and that was over 20 years ago. Experience in neonatal transport, we started our own ambulance service. And we started a neonatal critical care transport service, then, that was 19 years ago, doing all of the neonatal transfers for the hospital group that I attached myself to plus another private group. There were several hospitals groups at the time. Since then, life moved on. Several things been all over the place, but then came back to critical care about eight and a half years ago. We started off doing all sorts of interesting things with critical care with ventilation of the neonates from non-invasive, trying to figure out how to do high flow into and out of hospitals with portable cylinders, with dive cylinders, etc. So really just following international trends and trying to get up and running within the Western Cape, retrieving babies from outlying areas, bringing them back into level one hospitals and to the neonatologists, which there are about only 6 in the Western Cape. So, we were doing a fair amount of transfers. That's the background.

**Researcher 1**

So, you've got quite a unique background within this field, I would say. I don't know of any other nursing sisters, you know, and then also the EMS qualification, and someone that's been very part of the development of critical care retrieval. So, I understand it's different to let's say, your new ECPs or your older N.Dips. Which part of your education do you find most valuable, that prepared you for critical care retrieval of neonates?

**Participant 4**

I think it's a combination of both. I can't say that any one specifically equipped me for what I'm doing now. I think you have to take from both sectors and I think that's where the shortfall is, to be honest in the ALS or whatever you want to call it on road qualification. I think there needs to be, you know, we need to be working together.

**Researcher 1**

So, you obviously interact with other advanced life support providers. What has been the general impression from your side with regards to critical care neonatal transfers? Do you think they are prepared for doing it? Is there a shortfall?

**Participant 4**

Oh, absolutely not prepared for it. Just trying to get anybody to come and do voluntary shifts, or to take over a neonate is, a huge problem. I will honestly tell you, and I think you can back me up here. I'm absolutely terrified of neonates, and they all tell you the same thing. Just born out of not being exposed enough. And not having the background knowledge to deal with it. And understandably that I don't think they do have enough knowledge to stand alone, because that's what we do in the back of those vehicles. We stand alone.

**Researcher 1**

So, I guess it's then the lack of exposure and knowledge that then causes this fear or hesitance to work with neonates.

**Participant 4**

Lack of exposure, which leads to lack of what's the word courage, or I don't know what you want to call it, but familiarity. So, they're not familiar with what they're doing. So, they haven't got any, you know, learned procedures and experience within the procedures of doing it on their own and having to take the initiative and actually monitor and watch and look and make decisions and call on the right people when they need to. So, I think it's a mixture between education and the experience. You know, if you can educate somebody as much as you like, and I can use my own educational background and occupational health, I hold a degree in occupational health. Don't put me in an occupational health clinic because I wouldn't know what to do.

**Researcher 1**

Yeah. It's not what you do every day. I guess. So, I think we can't paint all the EMS qualifications with the same brush, but would you say that there is a general lack then within those programs of neonatal critical care content?

**Participant 4**

Absolutely. Definitely. It needs to be a specialty. I've been saying this for years and years and years that, you know, we need to have a general course like we do in nursing, you know, you become a generalist, and then you do your specialty. You can't squash everything into those four years, three years, whatever how many years you've done, you can't squash it all into there. Nor should you. Some people are just not interested in neonates, you know, I'm not interested in rescue. Yeah, specialty courses. That's what we need to develop.

**Researcher 1**

I don't know how familiar you are with the current ALS programs that are running. Do you think those curriculums will be able to be changed so that a person that walks out of there is completely prepared for critical care neonatal transfers, or do you think that time is not enough and additional training postgraduate is more in line with what's needed?

**Participant 4**

I think in order to make it safe for the practitioner and safe for the client, or the patient, and the treating team, I think it needs to be a specialty. You know, in, medicine, when anybody's dealing with neonates, it becomes a specialty anybody dealing in pediatrics, it becomes a specialty. Because they're not small adults. It's completely different medicine, you can't tag it on. It's not a tag. When you're dealing with these incredibly fragile, incredibly complicated transfers.

**Researcher 1**

Okay. You were obviously part of the development of critical care in the Western Cape. You must have identified some gaps along the way with regards to neonates and if you did have any, how did you close those gaps? How did you improve your knowledge in the field?

**Participant 4**

Well, basically what we did in the Western Cape is we identified in the beginning that we knew nothing, really about evolving care and treatment of neonates, because it is evolving rapidly. And you know, just going back from my day, in the beginning, where everything was an oxygen headbox, you know, you put an oxygen tubing into a goldfish bowl upside down over the head, and every neonate got the headbox. Down to now where, you know, we don't want to give oxygen. So, things were evolving rapidly with a neonate. Ventilation wasn't just, you know, you only had one form of ventilation, now you've got several modes of ventilation. And the distances, it was no longer we can't take that baby, you know, six kilometers, because you know, they’re going to die. Now you can take them six kilometers, you can take them six hours away, and they're not going to die because you're mimicking the transportation. The way we overcame the gaps was we chose the leader. Now, say we, there was always two of us as two of us now we've done a short week-long week to turn it out to offer a 24-hour service. We chose the industry leader, as far as neonatology was concerned. And we attached ourselves to him and said, okay, you know, we need you to criticize what we're doing. We want to know how we should be doing it, we want to know what you require. And this is pretty much what I've done. All right, from the beginning of when I started the neonatal transfer service, found the leader, you always find the ringleader, what do they want? What do they want you to do with their babies? How can you optimize their care, according to them? And how are you going to get them there? And what are they prepared to do for you? So, you we equipped the vehicle with what they needed us to equip the vehicle with. And we followed practices according to what they required. We kept in contact with them right the way throughout the transportation with the neonate, which we still do now. You know, if that particular neonatologist calls for us, we communicate right from the beginning. When are we going to get there? What did we find? How can we optimize? Should we be transporting? Shouldn't we? If we are what are we going to do if this happens in transit? What drugs are we going to use that we don't have? What should we be taking with? What can we get from the hospital? What can't we get from the hospital? What time are we going to be arriving? Does that suit you how all of these things? It's a communication. Let's look at the professionals. What do they need from us? What the South African environment need us to do to get those babies safely transported?

**Researcher 1**

Alright, so what I'm hearing is, like a mentorship by specialists, people in the field. And then also the exposure to these neonates and working alongside these specialists and then having conversations and that method of learning you found very valuable.

**Participant 4**

Yes, absolutely. Absolutely.

**Researcher 1**

Right. So, we touched earlier on additional training, postgraduate if we looking at the paramedic program, this type of education, if you had to put a timeframe on it, or the method of education, how do you see this type of training happening?

**Participant 4**

Well, I think, you know, after your basics, you'd have to try and figure out where the start off level would be and who you would, you know, bring into your course and what the baseline knowledge or knowledge gaps are. Yeah, I would hope like, you know, internationally and like a lot of other courses that it wouldn't just be leveled at the pre-hospital emergency care personnel. It will be leveled at the in hospital as well. So, you would be including doctors and nurses in this program in order to cross pollinate in order to make available in order to best utilize the resources available, so that we can, you know, purpose, the multitude of transportations that need to take place in a safe manner. So, I think identifying those knowledge gaps would be the first thing to do. And what are the knowledge gaps? And how do we address those. And then you could develop a course and the timeframe, I think, you know, online has become the, you know, almost known, thanks to COVID. So online would have to, you know, account for majority of it, I think if you look at people who would look at this course, after the basics, you know, you've done your basic four years, or whatever it is seven years as a doctor, you're not going to want to sit in college again. So, you know, if you're aiming to do ALS, they're not going to want to go back to tech, they're not going to want to go back to varsity. Online you can do so you know, a year's online course, followed by a, you know, the practice that you need to attain. Attaching yourself to hospitals, attaching yourself to services that are doing these, these type of transfers, that's how I envisage it happening.

**Researcher 1**

So, what I'm hearing is, we're talking about someone that starts working in the EMS or that is already working. And also, it needs to be inclusive. So, it can be for doctors that are interested in the field, and also nursing that's interested in the field. And so, for it to be all inclusive. You said it needs to be people that's working, so maybe a one year online so that it's accessible and possible. And that will be followed by more practical work, would you say?

**Participant 4**

Yeah, possible, you know, things and hurdles that they have to jump through. And however long that takes, you know, giving a limit to a year or whatever, I'm not sure. And I'm not sure, depending on what's put into the practical component, you know, how many hours they have to do in each? NICU, you know, each theater, on road, maternity, and primary health care, you know, depending on what you want to put into the curriculum, you know, how much time they have to do what waiting it is.

**Researcher 1**

So, would I be correct in saying that, because you feel this, this should be a specialized field. We're not looking at a one month or two months short course, we're looking at more of a postgraduate diploma or something.

**Participant 4**

Absolutely. Post grad diploma.

**Researcher 1**

Okay, that sounds good. So, you mentioned, that online is quite accessible these days, thanks to our current pandemic. You said that the practical work can follow that once the base has been set of knowledge. And where do you see this kind of practical experience happening?

**Participant 4**

I think you can draw, from all sorts of segments. I mean, you know, private, government, depends where your relationships live, doesn't it? I mean, we all know how this goes. You start off in the government sector, and they say, No, hang on, you're a private student. You start off in the private and they say, Oh, hang on, there is confidentiality clauses here, so go away. So, you know, I think that that's something that would be a tricky point, as always, is the practice you know, organizing the practice are always the worst thing. That's the job that you get that you hate doing. Depends on the political climate.

**Researcher 1**

So, I would imagine that working with the critical care retrieval team would be quite beneficial. But it is a very limited resource. So, I guess, working in the hospital environment would also be very necessary to close this gap.

**Participant 4**

Absolutely. But I think you know, you're not going to see all your anomalies on the road, you know, that you're in, you're probably in a busy NICU you see a lot of. Yeah, so you definitely have to and you know, to be attached to the neonatologist and their wealth of information, you're only going to gain that in hospital.

**Researcher 1**

So, what you saying is a bit of a hybrid approach between different units so that you can cover all the type of patients that you can see.

**Participant 4**

Absolutely, you know, just taking oscillation, for example, I mean, you're not going to oscillate somebody on the road. No, but you definitely need to know about oscillation. 12 lead ecgs on the neonate? I mean, sure, we don't do those on the road. So where are you going to learn about those things?

**Researcher 1**

If we can dig a little bit deeper into the content that needs to be within this type of education program, if we can give it some broad categories, what would those be?

**Participant 4**

Well, I think that your courses that you've given as a reading letter, actually are quite interesting. The ENCODE course, I'm going back to the nursing course, again, kind of summed it up quite nicely, because they started right from the extra uterine, and ran through what I kind of thought was really, really important topics, you know, going through the systems, respiratory care, infectious, neurology, metabolic, vascular access, which is vitally important, all of those things. So, they've done a nice, you know, that they've got a nice program, and from what I can see, but what they do lack is the pitfalls of transport. And the, you know, the mobile environmental physiology, the untoward events, movement and safety, which comes from your napstar Course. Yeah, equipment, tips and tricks, because you're not always going to have the same equipment. So, I think, you know, you could literally take the courses that are that you've outlined here, and pull pieces out and put them all in? To make a course. Because you, you know, if you look at the courses, they're all good. There’re good parts in every one of these courses, but you need to, you need to pull out the meat and throw away the bones. That’s the expression I think you need to use.

**Researcher 1**

So, what I'm hearing is, we can't just take one of those courses, international courses and use it as is in our local context for pre-hospital critical care retrieval. And that there are good modules within various courses, but they sort of need to be combined, to be what we are after.

**Participant 4**

Yeah, I would say that, yeah.

**Researcher 1**

All right. Did you look over the local curricula that the universities are presenting?

**Participant 4**

Yes, I did. I can't really comment on them. Because, you know, I am sitting here in the Western Cape, I've sat in on some of the lectures there. I've had loads of prac students come through me. To be honest, out of each batch of the pack students, you might find one person that's mildly interested. Okay. Two or three of them to actually know some of the stuff. So, you know, this all goes back to, I don't think it should be taught. I think it should be a specialty. So, whether there's a curriculum for it or not, if there's no interest in it. We need to define what the generalist is and what the generalist needs to know. And then we need to define what a critical care paramedic is and what they need to know. And I don't think there needs to be a great overlap at all, there needs to be a basic understanding. So, I think, you know, the course that they're running now in the critical care module can be born right down to, you know, a couple of lectures and separate out critical care.

**Researcher 1**

So, do you think the current paramedic program that the universities are running. The ECP programs, those newly qualified individuals, do you think they should be doing critical care retrieval of neonates straight out of the bat? Or do you think something else needs to be done first?

**Participant 4**

No, I don't think they should be doing them at all. But having said that, who's going to do them? Yeah, they are the most qualified people to do it at the moment. Yeah. So, you know, it's a yes or no scenario. Yes, they should be doing it compared to anybody else. Because at least they've had some training. Do they have enough training? No, I don't. So, is it leaving these people wide open for criticism? And disaster? Yes, it is. And are they prepared to take up that challenge? Well, some of them are, and are, you know, trying to do learning, you know, extra added on extra learning to try and help themselves along like you were and guided, and everybody else is working in the environment. Is it good enough? No, it's not. Thank goodness, we don't have medico legal attorneys wondering. Yeah, that's pretty much my thought on that.

**Researcher 1**

So, I guess we kind of stuck between the need for these neonates to be moved. But also, we have practitioners that are not completely prepared, sort of forced to do it.

**Participant 4**

Correct. And I also think, you know, that there's, we've got developments with the CPGs that are taking place. And then we've got the Health Professions council saying, incubator use, tick, tick, tick, tick, tick, everybody can use an incubator. You know, unfortunately, hospitals are saying I want this neonate out of my hospital. Now. I realize you can't take care of a baby with a drip. So, we'll just make it a j-loop. and off you go. Yeah, on your six-hour trip on the road. So, we've got you know, CPGs, which were a wonderful thing, the interpretation by health professions Council was a terrible thing. So, we're going backwards and forwards, backwards and forwards, we need you need something to tie it together. And I think making specialty is will tie things up. It'll tie things up for neonatal care for primary health care, for rescue, you know, all of those things that need to develop?

**Researcher 1**

So, do you think now that we talking about governing bodies, if we had to do develop additional education in this field and make it a specialist field? How do you feel about accreditation and buy in from governing bodies and how they should work?

**Participant 4**

Well, I think the buy in from health professions council will probably take you several years. And I think that would probably depend on the amount of practical application you add to it. Cuz I know they don't look too fondly on online learning. So, I, you know, I think you're definitely get buy in from the universities, they're always looking for challenges and looking for ways to earn income. And online is a great source for them right now, as they've just done the post grad, emergency care thing they've started. I think, you know, I think they would be very, very willing. So, I don't think from the university point of view, you would have too much of a struggle. Okay. I just think from the Health Professions Council, you that's where your fight would be.

**Researcher 1**

But would it make sense to say that, it would be best practice to say that people with this type of qualification should be the people doing critical care retrieval of neonates and not everybody?

**Participant 4**

Absolutely. Yes, definitely.

**Researcher 1**

But I guess then it comes back to how many people are actually available to do it, and how many people hold that qualification versus the need of how many neonates need to be moved?

**Participant 4**

Well, yeah, I think, you know, if you had to, if you had to look at it. I know we're very fortunate with our company that, you know, we've pretty much got a moratorium here that anything that smells sounds or looks like a neonate we'll be going with the critical care retrieval team. End of the story, not from our home to hospital, but certainly into facility. So, you know, if you get the buy in from the facilities, then that's just the way it is. Because, you know, not every theater cases a, you know, an open chest. You have tonsillectomies, too. So, it's the bread and butter that keeps you going. So, I definitely think there would be the volume. And I think it's those not so exciting cases that give you the courage for the exciting cases. So, I don't think volume is a problem. And I don't think sourcing the people to do it would be a problem. I think what people the problem people have is asking them to join a critical care retrieval team, when there's no training. They don't know what they're doing, and There's no benefit, monetary or any other way for them to be part of that team. So, I think once you develop that course you develop, you develop the, the desire people go right, I can actually specialize. Finally, I can specialize fantastic. And this is something I've been interested in. I've never said anything. And yeah, count me in. I'll be there. I can guarantee. I mean, you know, you'll have lots of applicants.

**Researcher 1**

So, you touched on this earlier, when you said, when you reflecting on some of the students that you've encountered, you said that some individuals are interested and some are not. And I guess there are similar trends within the nursing and the doctor programs where some people are interested in certain fields, and some are not.

**Participant 4**

Yes, yes. Absolutely. So, I’m sure you must have had this in your years in ccrs, that so many of the nurses in the NICU say how they wish they could be on the road with you, I would love to do this, it would be my ultimate.

**Researcher 1**

Yeah, no, we definitely came across that. So, the type of individual that should be doing critical care retrieval? When we look at? What type of person, personality, those type of things? What do you think that type of person that would be doing this course?

**Participant 4**

I think somebody that's shown an interest in pediatric and neonatal care. Obviously, if there's no interest, no point. Somebody that's, you know, trying to think of a personality that's a difficult one. It's really as I think you've just got to have an interest for it. an aptitude and an interest for it. I mean, it's like, who does primary health care? For goodness sake, that's just madness. Especially if you've got an interest and then you do it. There's nothing else that you really require. I mean, go and read a Shakespeare book. I couldn't. Yeah, but if I was interested in it, I would.

**Researcher 1**

So, I guess what we are saying is that, the most important thing is you need to be interested in the field, the personality traits. There are different types of people in our environment that do it? And it doesn't mean it has to be a specific personality.

**Participant 4**

Yeah, absolutely.

**Researcher 1**

And so, you mentioned something earlier, the payment for this type of training, do you see that as a barrier? Or do you think it will be easy to overcome?

**Participant 4**

Well, I think that Yeah. courses, you know, payment for courses they, how do people pay for their university degrees? I mean, there's bursaries you can have bursaries offered by you know, interests, interest groups, you can have a centers, medical, you know, offering a bursaries, you can have all sorts of institutions offering bursaries, you can have companies making it mandated, you know, companies might become a mandatory thing for us in this country to have the course. You know, if you want to do those, you can put it through the board of healthcare funders. Okay, so the only way you're going to get paid to do the course via the medical aid is if the people are qualified to do it. So, what are the private companies are going to go fantastic. We're going to have to get some people qualified here, right, that's enter a contract with you. The government system, you know, we want to say, I know what's happening here in the Western Cape, they've just bought out three ICU transport vehicles, and a couple of them are going to be doing the neonatal transfers. So, what do they need? They're going to need the course to keep up with profit. You know, they got lots, you know, they've got my tax money. So, you know, I don't see a problem there. They seem to be putting their people on all sorts of courses. So, I think there's many avenues to it. You just got to look broad. Look at your, your target audience and look at where the money's coming from with the private sector. Look at the motivations for the government sector. I you know, I just think just like any marketing plan, you would need to look at those factors.

**Researcher 1**

So, what you're saying then is there's enough ways to fund this type of training. And you just need a buy in from employers. And if it was a mandate from governing bodies, then this can happen.

**Participant 4**

Yep. And the funders. Yeah.

**Researcher 1**

Tell me, did you look at the first phase of my study with the neonatal data?

**Participant 4**

I did. I was interested in where, where that data was from I know it said, South Africa. Private or government?

**Researcher 1**

So, it was a national sample of private services only within South Africa. So, it's, it's obviously not the representation of all the neonates that were moved. And it was extracted from a bigger study that looked at all critical care transfers, not just neonates. But for this study, we extracted the neonatal data. But it gives us an idea of what type of neonates are being transferred, and medications, attachments and so on. When you look at those graphs, and what type of patients how does that look compared to what you guys are doing?

**Participant 4**

I did look at it, and I was, you know, my eyebrows were raised on a few of the things, but then I realized that, you know, this was South Africa. And obviously, you know, I think how can you do a lot of CHDs. We do a lot less we do a lot of respiratory. Okay. Obviously, we do congenital heart defects as well, but majority of ours are respiratory related. So, it's going to depend on areas. So, I think it correlates it probably would correlate This is research. It's done. It's dusted you know. Look on your medications. And right down the bottom, it's got phenobarbital, then I believe you guys don't use phenobarbital where we do. You know, that's what we use. That's, you know, patient their seizures. We use phenobarbital. We don't go for the midazolam. Attachments, capnography in neonates we don't use it, you know. 12 leads, we don't do. Oscillation, we don't do.

**Researcher 1**

Yes, I think the information that you see, it's not a picture of what's happening early during the critical care transfer. It's sort of how the patient was handed over. So, if it was oscillation, then the neonate would have been on oscillation and then taken over by them. And also, many of the medications is what the neonate was on when they arrived. They would have continued most of it. Some of it.

**Participant 4**

Okay, so it was the entire package deal? Yes. Okay. So yeah, so I did look over it. It's relatable. Yeah, it is relatable.

Interesting, because I don't know of any other studies that looked at it. And obviously, some services see a lot more of some patients and specific areas, like you say Western Cape might see more of some conditions. And I guess it also depends on the specialists that operate within those areas and what they do and what they prefer and all those types of things.

**Participant 4**

Yeah, absolutely.

**Researcher 1**

So, if we look at these neonates, is there anything that's specific that you would add to this type of training that we are talking about the additional education and to cater for these special needs?

**Participant 4**

I think where we need to develop and where the education might develop as in the actual transport mode, where, you know, overseas, they have special facilities that allow for non-vibratory non-bump sort of ride, where we don't have those. So, you know, we're increasing our triggers on our vents, so that we can, you know, not ever have a ventilated micro-prem, by the time we get to the other side, you know, there's a whole lot of advances that need to happen from start-ups or from vehicle developers. And that's something that we need to bring into the education side of it. That's not being properly taught, that should be taught, and it needs to bring itself up to international standards, if you're going to be teaching these courses, and it's a massive lag, it's something that I've tried to overcome, we've developed some system is still sitting with a developer that made half of it, it's been sitting there now for four years, and it's just, you know, it's mind numbing, you know, it's part of the lack we have in the country, and part of transport itself is a big shortfall, the actual mode of transport, you know, putting every baby possible in a helicopter, because we have the contract and we know we have a helicopter Not every baby should be sitting in a helicopter. So there needs to be some treatment and practice no no's and treatment and practice developments taking place, according to road transportation. Not yet developed, needs to be developed, a lot of research needs to happen on that side, I suppose it's just another factor, you know, once you start these courses, you're going to start a whole new research chain of events.

**Researcher 1**

Alright, so what I'm hearing is that there's a lot of considerations for out of hospital with regards to transportation, and then the modes, whether it be fixed wing, or helicopter or ambulance, and the exertion it has on the neonate. So not only do we still need to develop what we do in South Africa, but also, we need to consider that for education, because we need to teach these potential students then what to consider and which patients would do best in which environment and so on. Tell me your experience with various type of training, and then the assessment of students. What methods of assessment do you think is best to test the knowledge in this field? If there was education, to become a specialist in critical care retrieval services?

**Participant 4**

Well, I think if you're looking at making it a post grad course, obviously, you're going to have several methods of assessment. I think it needs to be an ongoing assessment, there needs to be an exam process, there needs to be practical assessments in the practical attachment section. There needs to be you know, verbal assessments, it needs to be orals attached. Orals are a very good way of putting people under pressure, saying, right, what would you do now, even the back of an ambulance bang, this is going down? I don't entirely agree with simulation. The way simulations are done. I don't think that did anything other than develop my knee jerk reaction, my nervous system, Twitch, and I think that's the only thing I've ever developed in me. I've Yeah, I think there's got to be an ongoing assessment, especially if it's online. I mean, you know, online assessments.

**Researcher 1**

So, you mentioned that oral assessments. It's a good method to test a student's knowledge. So, do you think it is very intimidating, or do you think it's at that level it will be a good method of getting information and probing more into what they actually know.

**Participant 4**

Well, absolutely, I think if you think about practice in the back of, you know, where, you're going to be treating these babies, you're thinking through the baby's problems, you're thinking through what you're seeing, and you're thinking through what you're going to be doing and how you should be doing it. Prior to transport during the transport and post transport. It's a constant process of thinking out how you're going to be going through this. And I think if you're thinking it out, you can talk it out. So, I think verbally, you know, if you have a verbal assessment, and you've got a patient, right, this is the patient, talk to me about this patient, here's a picture, how are you going to deal with that baby that you can see there on the screen? Simple. You know, this is what I can see this is, these are my considerations, this is what I'm thinking, you know, needs to be done. This is what I can see, according to the charts needs to be done. This is what registered nurse x, or Dr. x is just handed over to me, it's you know, not everything has to be handled in a college, these are very simple things. You can visualize it, you can talk about it, that's I'm going to do it. The practical stuff can be done in an attachment form, you know, later, where, you know, did she know how to draw up the drug? Did she know how to mix them? Did she know that certain lines cannot be disconnected other than during an aseptic technique? Did she know that an arterial line does this does that this is how you test it? Those are just tick boxes, those are there to actually think out logically and talk it out. That's what you need to know, you need to know that somebody is thinking about these things that they're considering. So, I think it's an excellent tool. And it's at our disposal online.

**Researcher 1**

All right. So, you're saying that verbal is then one of the best ways to really give a deeper understanding or assessment of the students understand?

**Participant 4**

Yes.

**Researcher 1**

And then do you think the simulations can be changed to be valuable or are simulations is not a good tool for assessment?

**Participant 4**

Well, you know, what I've just spoken to you about is probably a simulation. But it's not sitting in a classroom with a plastic baby having five cameras on you and seven doctors, and then under equipped jump bag, and, you know, that that's not useful. It's time consuming. It's resource consuming. And it's, it's, you know, what we're doing now, you know, on a team's call, you could have a picture up and a bunch of notes. And you know, your time goes now there's the notes, this is a baby you've been sent for. And here's a picture of the Registered Nurse, that's going to hand over to you. And these are the notes she gives you. sample that these this is your simulation? Well, you could be running through several of these on the day. This, could be an ongoing process of, you know, you've learned the respiratory system. Here's your assessment form. And these are how you're going to practice you need to do 20 of these in the next week. And then you'll have your final one, you know, it's all at our fingertips. Really, it is doable.

**Researcher 1**

Your opinion on mentors, mentorship, once people qualify from this program that we are suggesting, do you think it's important? Do they need to shadow first or can they start or what is your opinion on mentorship?

**Participant 4**

Mentorship or internship? Yeah, definitely. I think, most in Hospital Medical qualifications do just that. And yeah, it's vitally important because, they're say that you only learning when you're making mistakes. But wouldn't it be better if we didn't? Wouldn't it be better if somebody said I did this and it's wrong, do this rather, this is how you do it, or ah gees, this has just happened. And guess what, you can just do this stuff that the book doesn't teach you stuff that you know, the content, the content isn't there because people don't want to write those things down. People are too shy to write down their pitfalls. But you'll see the pitfalls on the road. You'll see the pitfalls in the NICU. You'll see the pitfalls in the theaters.

**Researcher 1**

Okay, great. So, we at the end of the interview. Is there a last message or something you would like to give if there was a committee that developed this curriculum? What is the most important thing that they need to take into consideration when they develop this course?

**Participant 4**

Don't lose heart. Go for it, make it happen. Because there are so many hurdles are going to come your way. But just go there. Just make it happen. And what do they say about procrastination? It's the fear of not succeeding. So, don't procrastinate. Just go for it. Make it happen. Do it.

**Researcher 1**

Great. Thanks. I'm going to stop this recording now.
