# Supplementary material for: Developing a South African curriculum for education in neonatal critical care retrieval: An initial exploration
Source: PLoS One. 2023 Aug 31;18(8):e0290972. doi: 10.1371/journal.pone.0290972 (PMC10470938; doi:10.1371/journal.pone.0290972)
Supplement: S1 Data — (ZIP) [file pone.0290972.s002.zip › Data Compressed/Codebook - Neonatal Critical Care Transport Curriculum Design 1 - 2021-08-05.docx]

Neonatal Critical Care Transport Curriculum Design 1

Nodes

| Name | Description | Files | References |
| --- | --- | --- | --- |
| Current status of neonatal CCR in South Africa | This section describes the current state of neonatal CCT in South Africa, as perceived by the expert participants. It outlines current practices, shortcomings and caseload/volume. It also outlines the importance of neonatal IFT. | 0 | 0 |
| Case Mix | This node describes the perceptions on the presented case mix of the experts. Instances of agreement and disagreement should be coded hereunder. | 0 | 0 |
| Agreement | This sub-category describes the perceptions on the presented case mix of the experts where agreement with the presentation was indicated. | 0 | 0 |
| Agree with representation |  | 4 | 9 |
| Differences | This sub-category describes the perceptions on the presented case mix of the experts where some differences with the presentation was indicated. | 0 | 0 |
| Different disease distribution |  | 3 | 8 |
| Other specialised medications |  | 2 | 4 |
| State less complex cases |  | 2 | 5 |
| Study should include state cases |  | 3 | 5 |
| CCR team members | This section describes the qualifications of the CCR team members and their distribution geographically | 0 | 0 |
| ANT's in rural areas |  | 1 | 1 |
| ECPs in urban areas |  | 1 | 1 |
| Variable competency levels |  | 4 | 22 |
| CCR transfer conditions | This section gives a description of the conditions that these CCR transfer are conducted under. | 0 | 0 |
| High risk |  | 4 | 6 |
| Limited backup |  | 1 | 3 |
| Limited Dedicated teams |  | 5 | 12 |
| Long distance transfers |  | 3 | 3 |
| Need improved systems |  | 2 | 2 |
| Time delays |  | 1 | 4 |
| Variable equipment levels |  | 1 | 1 |
| Need for transfer in South Africa | This category outlines the reasons why there is an essential need for the Interfacilty transfer of neonates, with specific reference to the South African context. It establishes the importance of having transfer resources available. | 0 | 0 |
| Critically ill neonate |  | 4 | 5 |
| Limited Neonatologists |  | 3 | 4 |
| Limited NICU units |  | 3 | 5 |
| Transfer to tertiary unit |  | 4 | 5 |
| Expert participant credibility | This section describes the expert group’s credibility to participate in the study | 0 | 0 |
| Experience | This section describes the experience that the expert participants gained in neonatal critical care over their career. | 0 | 0 |
| Extensive in hospital neonatal experience |  | 3 | 12 |
| Extensive neonatal CCR transfers |  | 5 | 20 |
| Neonatal educator |  | 2 | 4 |
| Formal education | This section describes the formal education that the expert participants had to make them credible for participation | 0 | 0 |
| ALS Paramedic |  | 4 | 10 |
| Neonatologist |  | 1 | 1 |
| Nursing sister |  | 2 | 3 |
| Paediatrician |  | 1 | 5 |
| Informal Education | This section describes informal education that the participants had in addition to their formal education qualifications | 0 | 0 |
| Transport courses |  | 3 | 17 |
| Ideal CCR team | This catgeory describes what the expert participants described as an ideal noenatal CCR programme/system. This included team composition, training and equipment requirements. | 0 | 0 |
| Advanced Equipment |  | 3 | 6 |
| Dedicated CCR teams |  | 6 | 19 |
| Extensive documentation |  | 2 | 11 |
| Multidisciplinary team |  | 4 | 13 |
| Learning and education in neonatal CCR | This category describes the current state of education (incl barriers to access) in neonatal CCR and how any identified gaps were bridged by expert participants. | 0 | 0 |
| Bridging the learning gap | Description of how expert participants bridged the gaps in their knowledge as it relates to current education in CCR in South Africa (other node). | 0 | 0 |
| Exposure to neonates in transfer |  | 2 | 4 |
| Mentorship |  | 4 | 25 |
| Self Study |  | 4 | 13 |
| Spend time in NICU |  | 4 | 10 |
| Current education in CCR in South Africa | This section describes the current education in neonatal CCR in South Africa, as percevied by the expert participants. | 0 | 0 |
| Additional education in Neonates required |  | 6 | 38 |
| Limited neonatal exposure |  | 4 | 16 |
| Limited time to cover neonatal content |  | 5 | 35 |
| Not local context specific |  | 4 | 7 |
| Not prepared for neonatal CCR |  | 4 | 16 |
| International Neonatal Curricula |  | 0 | 0 |
| Barriers to access |  | 0 | 0 |
| Barrier Physician only access |  | 1 | 1 |
| High cost |  | 1 | 1 |
| Limited courses |  | 1 | 1 |
| Extraction of sections from curricula |  | 3 | 6 |
| Proposed curriculum elements | This category outlines the structure of the proposed course, including cost, duration, assessment and learning methods, and the key learning outcomes - as outlined by the expert participants. | 2 | 2 |
| Cost and funding |  | 1 | 1 |
| Funding needed |  | 1 | 3 |
| Self funding is barrier |  | 1 | 5 |
| Duration |  | 1 | 1 |
| Continuous education |  | 2 | 7 |
| Determine outcomes before timeline |  | 2 | 5 |
| Introductory course (Lead and secondary members) |  | 6 | 28 |
| Masters degree (Lead members) |  | 3 | 11 |
| Not short course |  | 3 | 3 |
| Part time |  | 1 | 1 |
| Post graduate diploma (Lead members) |  | 6 | 21 |
| Unknown duration |  | 3 | 5 |
| Learning objectives | This section describes the learning outcomes of the curriculum that the expert group suggested. | 0 | 0 |
| Anatomy and physiology |  | 3 | 5 |
| CCR Systems |  | 4 | 6 |
| Adverse events |  | 1 | 4 |
| Appropriate receiving facility |  | 3 | 5 |
| Escalation pathways |  | 2 | 9 |
| Network limitations |  | 2 | 3 |
| Patient criteria for transfer |  | 3 | 14 |
| Conditions |  | 2 | 4 |
| Conditions from study |  | 2 | 4 |
| Congenital heart defects |  | 4 | 14 |
| Infection |  | 2 | 8 |
| Prematurity |  | 2 | 3 |
| Respiratory |  | 0 | 0 |
| Bronchopneumonia |  | 1 | 1 |
| Diaphragmatic hernia |  | 1 | 1 |
| Meconium aspiration |  | 1 | 1 |
| Persistent pulmonary hypertension |  | 1 | 1 |
| Surgical emergencies |  | 1 | 1 |
| Gastroschisis |  | 1 | 1 |
| NEC |  | 3 | 5 |
| Continuity of care |  | 0 | 0 |
| Feeding |  | 2 | 6 |
| Skincare |  | 1 | 4 |
| Documentation |  | 1 | 7 |
| Emergency Procedures |  | 0 | 0 |
| Airway management |  | 2 | 4 |
| Chest decompression |  | 1 | 2 |
| Resuscitate |  | 3 | 4 |
| Equipment |  | 3 | 8 |
| Incubator |  | 1 | 2 |
| Infusion devices |  | 2 | 3 |
| Troubleshooting |  | 2 | 3 |
| Indwelling attachments |  | 0 | 0 |
| Colostomy bags |  | 1 | 2 |
| Medication |  | 4 | 14 |
| Pathophysiology |  | 3 | 6 |
| Patient assessment |  | 4 | 21 |
| Patient monitoring |  | 2 | 2 |
| ABG |  | 1 | 2 |
| ECG |  | 1 | 1 |
| ETCO2 |  | 1 | 2 |
| Fluid balance |  | 1 | 1 |
| Glucose management |  | 2 | 3 |
| Perfusion |  | 2 | 6 |
| Thermal regulation |  | 4 | 12 |
| Transport considerations |  | 3 | 4 |
| Acceleration deceleration |  | 1 | 2 |
| Modes |  | 4 | 10 |
| Movement and sound |  | 3 | 7 |
| Patient packaging |  | 6 | 17 |
| Vascular access |  | 0 | 0 |
| Arterial Lines |  | 1 | 1 |
| Central Line |  | 1 | 1 |
| IO access |  | 2 | 3 |
| IV peripheral |  | 2 | 4 |
| Umbilical |  | 1 | 1 |
| Ventilation |  | 6 | 20 |
| Bag valve mask |  | 2 | 3 |
| CPAP |  | 3 | 5 |
| Heated circuits |  | 2 | 2 |
| Humidification |  | 4 | 4 |
| Neopuff |  | 2 | 2 |
| O2 Blending |  | 1 | 3 |
| Oscillation takeover |  | 2 | 2 |
| Ventilators |  | 2 | 2 |
| Scope of applicability | This category outlines the scope of who the course is applicable to, hhow it is standardised and who is eligible to enrol. | 0 | 0 |
| Inclusive |  | 5 | 10 |
| National standard |  | 5 | 11 |
| No change in scope of practice |  | 2 | 4 |
| Teaching Learning and Assessment |  | 0 | 0 |
| Method of assessment |  | 1 | 1 |
| Continuous assessment |  | 4 | 9 |
| Discussions |  | 3 | 11 |
| Oral assessment |  | 6 | 19 |
| OSCE Skills assessment |  | 5 | 8 |
| Portfolio of evidence |  | 3 | 12 |
| Simulation |  | 6 | 19 |
| Written assessment |  | 4 | 10 |
| Teaching Method |  | 0 | 0 |
| Interactive |  | 4 | 10 |
| Mentorship |  | 6 | 19 |
| Online learning |  | 6 | 12 |
| Specialists discuss topics |  | 1 | 3 |
| Work-intergated learning and clinical placement |  | 5 | 9 |
| CCRS vehicle |  | 5 | 12 |
| Control room |  | 2 | 4 |
| EC that receives neonates |  | 3 | 12 |
| NICU |  | 6 | 28 |
| Theater |  | 1 | 1 |
