# Supplementary material for: Developing a South African curriculum for education in neonatal critical care retrieval: An initial exploration
Source: PLoS One. 2023 Aug 31;18(8):e0290972. doi: 10.1371/journal.pone.0290972 (PMC10470938; doi:10.1371/journal.pone.0290972)
Supplement: S1 Data — (ZIP) [file pone.0290972.s002.zip › Data Compressed/Transcript 5.docx]

Interview 5

**Researcher 1**

Just to confirm on the recording that you give permission for me to record?

**Participant 5**

Yes, I do give permission for you to record.

**Researcher 1**

Perfect, thank you very much. I sent you the consent form and to summarize that form, all it says is that this interview is voluntary, and you can withdraw at any time. And then if any personal information comes out during this discussion, I will anonymize it during the transcription. Are you happy with it?

**Participant 5**

Yes, that is perfectly all right.

**Researcher 1**

All right. So, Doctor, just a quick background to the study. So, my background is I'm a paramedic, I currently work in the state sector. And I've also worked in the private sector. And I've done a lot of ICU ambulance transfers in both sectors. And the general consensus between myself and my colleagues are that with these complex transfers, we feel that we were not prepared for them. Our education did not really prepare us adequately to handle these sick new neonates. I think that's how the study originated and this is something we are trying to change. Historically there are also variable education backgrounds. Paramedics have different qualifications. It used to be short courses. Now, it's only higher education courses. So, it's been a very confusing last decade, for our industry. We are trying to close this gap but even at university level with a four-year degree, similar to the nursing degree, I guess, there's only so much time and you cannot spend years on preparing someone for neonates so we feel that there should be additional training to help us with it. So that that's roughly the background and why are we having this discussion. Can you start with just telling me about yourself your background, where you worked and your education?

**Participant 5**

Okay, um, so I qualified from medical school and my undergraduate and then I stayed in the same university circuit and did my pediatric specialist training there at two state hospitals and then there after I did a little bit of general pediatrics for about a year or so with neonates and then went back to the state hospital to work in the neonatal unit there which includes neonatal ICU, transitional care, low birth weights, KMC, etc. A very big unit and I basically was there from 1996, up until about 2004. I did register as a neonatal sub specialist in 2002 based on time remembering those days, they weren't these exams, but we did register as neonatologists. So that's basically my background. I've done subsequent to that some work in the district. In Johannesburg district as the district pediatrician for about the last eight years, that's where I've been, but I've done teaching in between, at medical school, at the university, etc., and a little bit of private work, but I am essentially a neonatologist still at heart. I've taken early retirement, but I'll be going back to do some sessions in public. Hopefully in the next month or two, I'll be starting sessions again. So that's where I come from.

**Researcher 1**

So, I know that in state and government sector, a lot of neonates are being moved on a daily basis. Obviously, there are only so many specialist centers and a lot of patients. So, the aim of this study is targeted at the high acuity patients, so not so much the patients without oxygen and attachments, that would be okay for kangaroo care, we are looking at the specialist transfers with ventilation or CPAP, and multiple infusions. And you know, some of these transfers can be quite complex. So, that's the level that we were looking at, and also the document that I sent you, that data, it was of that type of neonate that was being moved by critical care transfers as we call them. So not, the big volume of neonates moved by ambulances in general. Tell me, doctor? Did you physically receive patients from EMS? Or was it your juniors that would receive them and then you would consult after? What was your experience over the years?

**Participant 5**

Well, mixed. The sickest ones that were coming through to the neonatal ICU itself for ventilation or for surgical management, for example, were received, often by ourselves as the consultants in the neonatal ICU with registrar's as well. And so, we did have some interaction with the ambulance staff. And interestingly, the reverse when I was working in the district, we would sometimes be in a midwife obstetric unit where there was a sticky, a little newborn needed actually to go across to a tertiary hospital, and actually was intubated, say by us, because we happened to be there at the time, and then was to be retrieved by an ALS team. So, we sometimes had the experience of being on the other side, we were handing over to an ALS team to take up. But much of my time, I was on the other side and receiving such an infant from a team that would have had to retrieve the child and was now bringing the child in.

**Researcher 1**

And tell me, when you look at this data that I presented. Just the background on that sample was not from state transfers, it was from a national sample of private services. The reason for that I'm not sure why it was only private. Maybe it was easily accessible, or I'm not sure, convenient. But when you look at the spread of those patients and medications, attachments, is it similar to those sick patients that you would have received? Or did what you received look different?

**Participant 5**

I think I was going to ask first of all, you know, who the sample, where it was collected. Because it struck me that almost about 16 or 17%, the top diagnosis was congenital heart defects, which would have not been our number one in the provincial sector at all. Our number one would then be respiratory distress syndrome / prematurity, perhaps followed by something like birth asphyxia. Yeah, and low birth weight, something like that. So, in actual fact, that was the one surprise for me, and I suppose that does explain it if the sample was a private hospitals, because it is a little unusual to have that as number one in in the public sector. So that was the main difference that I noticed there really. But obviously, if you've got preemies with respiratory distress syndrome then maybe many of the attachments would be similar, but then would be far fewer, who would need inotropes and things like that. So perhaps be a little bit less cluttered and a little bit less complex, maybe very sick, but maybe not as complex as this. We occasionally got into hospital transfers, when, for example, there were no other beds at other tertiary institutions, or the institution did not offer the service like surgery for neonates. Then would get slightly complex cases. But yeah, certainly the cardiacs are not a common feature for what we will see on our side.

**Researcher 1**

Alright, so I think that's why it's important, for us to have the discussion because you need both sides of the story. And I've worked on both sides, and in the private sector the cases can get quite complex and overwhelming for a paramedic, especially a new paramedic, because, the number of attachments and methods of ventilation. Sometimes these neonates are on nitric oxide gas mixes or oscillation, and then you need to take them over, or congenital heart diseases and the oxygen concentrations are very important and the infusions. So, those cases can be very overwhelming for people like us. In the state sector, also very sick babies, but I've never transferred a congenital heart defect baby in state. I've done mostly like sepsis or prematurity. You know, those type of transfers and the attachments are less complex or less so. Why there is a difference, I don’t know. And I think that the point is on both sides, it can be a bit overwhelming if you're not supervised or experienced. So, it's important for us to say, what do we think we need to teach paramedics after they're qualified to help them close these gaps? One of those case demonstrations I did, there was some of the attachments and medications that these babies had during the transfer. And I'm sure it's was quite a tricky transfer for a medic to do.

**Participant 5**

Absolutely. Yeah. I mean, one of the things that I've been thinking about and we've talked about it to some extent, you know, there was and from the provincial, Maternal Child, women's and nutrition department of the province. About two years ago, we sat together and as neonatologists from the province representing some of the tertiary and maternity hospitals in the province to write up a kind of neonatal plan for the province. One of the areas we did touch on was transportation in neonatal transportation. There wasn't necessarily consensus, which often happened. But my own personal opinion, was that and you made some reference to it when you talked about the international standard is that transfers or transport of neonates, especially the sick ones should actually be a multidisciplinary team event? I don't think that it should be done by paramedics alone. And my suggestion had actually been that, they should be like they do overseas and either neonatologists or even pediatric registrar's in training, and or even clinical associates who are they can give them a specialty in emergency medicine or transportation to work together with the paramedics as a team. I think that's the best way to transfer neonate but I don't think it found traction necessarily in that discussion at that time. But I see that that I think that that would be a much more workable way of doing it and less stressful for the individual paramedic, for example?

**Researcher 1**

So, this has also been the consensus amongst other conversations or interviews, that would be the best approach to have specialized teams. And if you can have doctors and nurses and paramedics mix, just to give that neonate the best chance. But I guess in South Africa we have limited resources. So, I don't know how long that would take to get something like that on the go? Because, these transfers are high risk, and these neonates are very sick. And I've found myself being alone in the back of that vehicle with a neonate, and it's a four-hour transfer. And it is extremely stressful and, overwhelming. So, you know, this is what's happening at the moment. This is why having this research or the need for it.

**Participant 5**

I wanted to just check with you, what sort of proportion of your cases are these critical? critically ill neonates? As an advanced life support? What proportion of your cases? I mean, is that every day you would have one? Or does it depend on how you allocated or how what burden? You know, what sort of percentage burden would that be of your time?

**Researcher 1**

I think this is a difficult one to answer. Because when I was with private, I was on a critical care retrieval unit. So that's all we did. And I would say eight out of 10 transfers were neonates. And on average, we probably did about two neonates a day. And we only took neonates that were on CPAP or conventional ventilation. So that was like every day in the private sector for that company. And I think other private companies, the bigger ones, they move similar volumes on a daily basis. When it comes to the state sector though, not that many. If it was probably like five a month, it would be a lot. And yeah, I mean, you got the NEC cases that went for surgical consult and they were very ill. But the volume was not as much. I wouldn't be able to say why. Or, I guess the hospitals that we took them from would be able to manage them or I don't know. But in private sector, a lot of these babies are being moved, I must say.

**Participant 5**

Okay. All right, I've got a sense now.

**Researcher 1**

So, like I said, there was a general consensus that we feel there needs to be additional education for paramedics. But it doesn't only have to be limited to paramedics. So, what we were thinking or the consensus was that we're looking at like a postgraduate diploma or master's level, and this should be the standard of education for this, this complex field, but it shouldn't be reserved for paramedics only it should be for nurses as well. And also doctors that are interested in the field and the paramedics with different backgrounds in education. So, whatever the NQF level was, or which University they attended, you know, it should be inclusive for everybody. I don't know how you feel about that. Does it sound about right or do you have another suggestion or opinion?

**Participant 5**

Yeah, in fact, I wrote down on my piece of paper here that I had circled here something like a one-year qualification, diploma, for example, a diploma in critical care transfer of the neonate or something like that. So, it sounds about right to me. With opportunity, I think for plenty of practice, yes. But that already implies that they bring somebody to supervise or be present, from whom you could then sort of be the mentor to give the guidance. So that's obviously something to think about. Who is that? Is that someone who's been through such a course before and becomes a trainer themselves? Or are you looking at a neonatologist or you're looking at a mix of people? But that to me sounds about right. When I was sort of mulling over what kind of thing it would be. Yeah.

**Researcher 1**

Yes, the common theme that's come through so far has been, it shouldn't be a short course, like an ACLS of a couple days It should be longer. And everybody so far has put a lot of emphasis on practical exposure. There’re not many specialized retrieval teams to work on. So, a lot of people were saying in hospital exposure, whether it be NICU, or casualties, that see neonates and so on. But something that also come through, people feel that this should be, you know, an accredited course or, like a national standard. It doesn't help we change something in a private company only. It needs to be buy in from regulatory bodies. I don't know how you feel about that.

**Participant 5**

I would agree with that. I mean, I think that's very important. Because it allows you to be able to standardize, you know, you set your level, this is what we expect. And then you standardize that across the country. I mean, it has lots of other implications, it means that if you work in Cape Town, and you want to go to Joburg, you know, you can move your resources, so to speak around, but you can also be reasonably assured that you've got the competency that you're looking for, I think it would probably need to go along with a refresher, you know, refresher course, to just, you know, if there's new things coming up. However, you want to set it up, whether it be every year, every two years, or something like that, then to let people just get CPD points, and so you know, be able to keep up with things that might be new, but i would i would agree that it shouldn't be something that's standardized, and accredited.

**Researcher 1**

Then, so looking at your exposure to the types of neonates that were transferred, and the document that I sent you with the type of patients that private companies are transferring? Can we try and give some kind of an outline of important topics or content that should be covered within this type of education? I don't know what your thoughts are on that.

**Participant 5**

I want to look at some of the things that you put through some of the courses that came through. There is a page by page eight, where you will look at the curricula of the universities that had some neonatal transfer content. I think its page eight, where the big table starts. Yes. So, I mean, some of it is not all necessarily because it's some of it talks to long term management in an institution like under the general Section there. You would have to understand something about the critically ill infant and where you are taking them. So yes, the functioning of an ICU or need for ICU, and general areas of importance in the newborn particularly. I like that they put maintaining a neutral thermal environment there. I mean, we know that that's very important. Hypothermia is a predictor for death. And included is metabolic stability, particularly something like managing hypoglycemia. And that you could package however you want to whether you want to put it under a general knowledge, whether you want to look at specific modules and break them up, according to body systems, or just sections. I like that the ventilation section, the way they have put it out, I mean, you can play with that. But almost all that they've got in there, I thought was quite relevant. They didn't really talk about oscillation there but I mean, something like oscillation would obviously be important, because you're going to be taking through babies with meconium aspiration and persistent pulmonary hypertension. Many of whom need oscillation though, that I like the fact that patient monitoring is a separate section, because it really sorts of is. There is such a lot to monitor on the sick newborns that are coming through, though, quite like the way they've set it out. I mean, you can play with that added subtracted packaging, or something like that. But I think much of what is in there is correct.

**Researcher 1**

So, that study was a comparison of the four universities on the degree level, so that's the four-year course. So, it's the critical care section of the curriculum. And yes, it includes adults, then all the way down to neonates. So most of them did not focus only on neonates as a separate entity. Maybe mentioned the special considerations under those headings. And then, speaking to some of the lecturers on these programs, they recognize the need for the time that needs to be spent for neonates specifically. But it's sort of mixed in between everything in the final year. So, I think that may be a problem, but it's also the time limit. Because if you spend more time only on neonates, then something else will have to be sacrificed. I think that's the problem.

**Participant 5**

That was the problem they had, you know, but all of these topics can be applied to just the neonates. So, the headings can be applied to the neonates themselves. And I liked the emphasis on fluid balance, because that's one of the main things. Maybe like chest X-rays may not be that relevant here. But preparation for transfer, which you would adjust I suppose, because it's got to do also with including the mother. And in terms of you might have to take her with or you at least have to communicate with her and get a history or something like that. So, we're the pregnant mom comes in, you need to spend some time on an understanding her and her condition, because that might have impacted on the neonate themselves. So yes, I saw your note that it wasn't like a standalone module, but just the sort of heading that they've included, I thought was quite useful to structure around. What I thought I didn't see. And I'm not sure if in the other curricular courses, you've mentioned whether they talked about them or not, was surgical emergency in neonates. There were one or two I saw, you had a few gastroschisis and things that were transferred but I think surgical emergencies would probably be a little standalone. For gastroschisis, diaphragmatic hernia although it's repository in presentation is surgical. And I thought that might be important just to think about as a section to add. I'm just trying to quickly see that nursing that last one, I think the nursing course, which was a yearlong, that you referred to, also, I thought, I like at the first part of that first module, I thought was very important, for example, transition from fetal to neonatal circulation etcetera. You know, there's almost a whole module on that, which is important to understand. And they also structured it quite nicely. Obviously, not all of it is necessarily relevant for just short-term transportation. But, you know, the respository care is looking at almost the majority of things that in a public sector we would be very familiar with. And those would be subjects or topics that I think that would be important for someone transferring a neonate to understand well, in terms of managing the neonate themselves.

**Researcher 1**

And then I've had different opinions on the approach with regards to should you really know the, let's say, the congenital heart defects, the pathophysiology behind that? To manage that patient, or most you just understand your systems approach, your respiratory, your cardiovascular and how to manage that presentation. How do you feel about that?

**Participant 5**

Um, to be honest, I think it would be a bit difficult to manage them well, without having a fairly sound understanding of the pathophysiology. I know that with cardiac conditions, it can be a bit tricky. And you'd need to figure out how you present the different congenital conditions whether you package them in a certain way. Because it's going to be a clinical approach, you may or may not have a specific diagnosis. It was just born and they happened to be very sick, but they were born in a place that can't say what's wrong with them, you just know that you've got a cyanotic neonate. And there the approach is the cyanotic newborn? How to manage that, what are the possibilities? What are the critical things to remember? And what's the safe way to manage such a child. You know you want to probably keep the ducts open, you probably need to give Prostin but you need to watch for complications. And you might not know exactly what it is, but almost the safest way to deal with children. But I think you would need a little bit of insight into what the possibilities could be so that you could explain maybe slightly further what other options would be available or what else to look out for if the first thing you do doesn't really work or makes the infant worse. So, I think you should have some competence in those areas to allow you to be able to troubleshoot in a way and manage them competently.

**Researcher 1**

Alright, so what I'm hearing is a bit of a mixed approach. So, I think it depends also on your setting if I can refer to myself. Sometimes you picked up a neonate from a setting where a specialist has already made the diagnosis with extensive notes so you already know exactly what is going on. And it's just a continuation of the care. Sometimes it is very early stages and like you said it will just be a cyanotic neonate so obviously not our place to make the definitive diagnosis but you need to troubleshoot and your management will be based around that. Is that correct?

**Participant 5**

Yes, yes, I would say so.

**Researcher 1**

So, you already mentioned, you think, like a year program would be practical? How do you think that should be structured if we look at method? Is it in class? Is it online? Is it a mix? Is it only in a hospital? How do you think that should be structured?

**Participant 5**

Okay, so I mean, I'm not a huge educationist. And then I haven't got experienced per se, in the theory of education. I would think you have to be able to give some sort of background theory. Corona is obviously influencing how we think about giving it you know, information, sharing information, if you like. So, doesn't have to be a classroom, but you know, it needs something that has opportunity for people to ask questions. If you provide videos, for example, video teaching, or lectures, PowerPoint lectures, let's say for argument's sake, there should be opportunity for question sessions. Question and answer session so that students can clarify certain concept videos. I think they are very nice, specifically for explaining skills, particular skills, because you can follow the steps quite nicely often on a video. But then you would have to have the opportunity to do simulation practice for example. And then ideally be able to do whether it's five or 10, or whatever it is, you can decide in your logbook. Which skills are required to qualify for this course? I mean, to just to complete the course to be competent, how many there are, and they probably would need to be done in an ICU environment, where somebody who's trained, can observe the person doing the skill, and be able to, you know, walk them through it, and then they can do the prerequisite number of interventions, whatever it is that's required. So, I would see it I think it should be very practical. The focus should be on getting enough exposure at a practical or at a clinical level once these theoretical background are covered.

**Researcher 1**

So, what I'm hearing is an initial phase that covers the groundwork or the base knowledge. And it can be online, it can be maybe the current setting, not so much in a classroom, but online, but it needs to be able to ask questions. Videos need to be interactive. And then once that is covered, then followed by practical, which then you need to demonstrate a portfolio of evidence. So, you need to have a logbook and your skills and you need to have a certain amount specified to finish this course. Is that correct?

**Participant 5**

Yes.

**Researcher 1**

So, some people have mentioned before that with paramedic students. We're not the front-line people in the ICU. So, they often tend to just observe from the side. But it depends on especially if it's a sick neonate, then you understand why but the opportunity to really touch that patient and do these skills is quite limited for us. I don't know how you see that?

**Participant 5**

Yes, I understand how that can be the case. But I think what I would hope would happen is that with the development of the course and neonatologists working in neonatal ICU across the country, would be brought on board in the sense that they would understand that there is a course like this, that is either being developed or has come into being or something like that. And that whatever accreditation body could literally write to the South African Pediatric Association, for example, just as an example, and say, this is happening, whatever it is, please know that in your institutions, we will be having students who require the opportunity to do practical training, and they would need your support to allow them to do this to be hands on. And then it would allow it to be to be genuinely understood. So that when arrangements could be formally made like, when students of various categories come through to the wards, it's known, okay, for this week, or change, we've got these types of students who are coming in and they're going to do this. We know that the interns when they have got to do five, whatever, the six, whatever's. I think if it's communicated beforehand, it can be helpful. I know, in some instances, it's tricky. I mean, if the kid crashes, you might not ask the paramedic to intubate. But we do that with a registrar. We're standing next to the registrar. And we say, okay, it's very reasonably controlled. I'm bagging the child for you now, you intubate, I'm standing next to you, I'm going to let you try one time or twice if you fail, I will step in. I mean, it's not impossible to do, I just think that if it's well communicated, and it's accepted, or understood, then it can be structured in a much more structured way. In private, I imagine it will be difficult. But in provincial, you know, places where training is happening in different categories of stuff more regularly then I think it can be done.

**Researcher 1**

So, you don't learn much just by standing in the corner and observing. You need to be part of it. But like you said, especially if it's a stable neonate, and that's controlled and enough supervision, then I'm sure there's enough opportunity for hands on experience. All right. So practical experience important. The base knowledge, theory should be covered first. Your opinion on assessment methods? What has worked for you, or with your students? Or what have you seen that works, that's valuable?

**Participant 5**

Honestly, I must say that, in an ideal setting, I suppose one would say a kind of mixed type of assessment. Some degree of theoretical understanding, but I wouldn't make it too heavily theoretical. Because the true work is handling both the neonate and all the attachments, plus machinery or equipment. That's really the job. I would include assessment of those aspects of the management of such a sick neonate. And I now know it's going to be slightly tricky. But, for example, you can do scenarios, case scenarios, whether you paint the picture as an example. And then you ask the person to manage the child for you, and you can set up your stuff, or get them to set up the necessary equipment and tell you what they did. This is this and here's what I'm going to do. And here's this test. You might want to have also a mix of paper cases. But I think that it would be very tricky to do an actual real-life assessment. Because that's very patient dependent and opportunistic dependent, and it's a little bit difficult. But if I guess if we really wanted to you might be able to arrange one or two of such assessments, where you've got the examiner or the supervisor riding if you like, with the student on the road. Okay, but obviously, it's not very easy to get that right because it depends if you're getting cases every day, that's fine. We know one case will come in the day, I can ride with you all day. But it would be a little bit heavy, I think on resources in terms of the examiners. So, you might have to either do a partial assessments, you know, bits of the assessment, say, in the neonatal ICU. Other bits might be on the vehicle. You can do it in portions. And otherwise, you might have to do it by case or by simulation. Yeah.

**Researcher 1**

I agree with you. Because I would imagine that the person that would examine or supervise would be someone like yourself, and to ask you to spare a week of your time to try and find a complex case, just so you can see what we do, that would not be practical. A nice suggestion that I've heard before was, we would go to a NICU, and you would have a specific neonate, maybe a complex neonate. And then you would have a simulated oral exam where the student would assess the patient in front of you, and then just verbally discuss how would you manage this patient? How would you transfer without actually doing the transfer? Because it is very high risk with actually moving the neonate. And you wouldn't do it if it's not necessary?

**Participant 5**

Yes, that would be sufficient. Normally you'd want to see, but that you can simulate. So, for example, you know, the handling of the machine and things like that, you know, whatever. Some of those you could do, but the one you described is probably the most efficient and practical.

**Researcher 1**

And tell me, doctor, the equipment that you've seen paramedics arrive with, how does it compare? Is it very different? Or is it similar to what you would have in the ICU? What was your opinion of the equipment?

**Participant 5**

So, I mean, I suppose it's all linked to resource and provision and constraints that might be within the public sector, but the transport ventilator, for example, I mean, we were not originally familiar with them ourselves, because they are many of them actually, quite simple. The ones we saw, yeah. And other things would be slightly different, you know, everything, it tends to be smaller. Everything is mobile. So, a little monitor for SATS a little everything is tinier, a smaller oximeter, a smaller everything. So, we would had to figure out. Oh, this is what it is, this is where you do it. And this is how you use this particular equipment. What I think was a bit concerning, and it's out of the realm of the education, per se, but it's more to do with resources, as I've mentioned. Are competencies was one that sometimes the staff weren't perhaps as competent as we thought they would be. But sometimes they were also let down to a certain extent by the quality of the equipment they had. And the fact that by the time delays, by the time they reach the neonate, they're going to treat. Because of the time delays very often that child was much sicker than they probably would have been if they had been able to get there much quicker. So, all in all, you find that they're in a very difficult situation sometimes. So, I think for me, those were the things that I mainly noticed, and then we also found the documentation of the transfer and that the patient's vitals during transfer, were sometimes not as well done. So those were areas that could be improved on.

**Researcher 1**

This has come up before. We spoke to a specialists that worked in the UK and she also mentioned documentation is very lacking in South Africa, maybe medico legal wise we not at the level that they would have been at, but definitely room for improvement. And then. Would it be fair to say that this should be included in this type of education, the importance of documentation, what should be included, and so on?

**Participant 5**

Absolutely, and I would actually test that as part of that case scenario description in the ICU. I would love to see them actually writing, fill it in, you know. This is the case that is at this point. So, I really think it deserves that level of emphasis.

**Researcher 1**

Then you mentioned a problem with the system. So, whether the problem is on the control room side, or the length for dispatching or mobilizing on the ground, or however it works, would it be then fair to say we should include an understanding of the system the EMS or the dispatching system and how that should work?

**Participant 5**

I think so. What we understand is that the advanced teams are very few. Yeah, the number of ALS trained are very few as all the vehicles, ICU, you know, buses and things. So that is a resource issue. So, it's sort of painful in a way that he would be training people and they would have the requisite skill. But, you know, because of system challenges, they actually have to have a more difficult job. So, you know it's even beyond just the curriculum, they would know what to do. And they would know, it should work like this. But the system itself might actually disadvantage them. So, it's very rare that they delay, it's just that they don't have them. And so, they would send the basic guys, which I understand is how it works. Just to kind of, I don't know, be there. While the ALS guys are still coming.

**Researcher 1**

Yeah, so I think with our resource limited setting, they’ll send the closest team that's available, and then they'll wait for someone that can actually do the transfer. And then it's similar with private, they often come from far, because there are very limited teams, and they feel that those guys must be moving them and not anybody else. And then there are big delays.

**Participant 5**

In public, I wish they would do what they do in private. I mean the guys that was driving a response car. But I think you need that kind of thing. So then even if the first guy can get me to start stabilizing, while the bus comes with everything on it is fine. You know, it's better than nothing at all. So, I would say along with this wonderful, you know, development of the of an actual course, would be intended improvements in natural systems. So, something like that, I think would also make a huge difference in the outcomes of the neonates.

**Researcher 1**

So, we've got a long way to go. Yes, there are paramedics on response cars, but I think the need from the primary emergencies is very high. And then by the time the neonatal transfer comes along, to get them to be available or rush. Yeah, so it depends. I don't know what the answer is, or what works best. But there's a lot of room for improvement. I do agree. And then considerations for different modes of transport. Have you got exposure to the effects of being in a helicopter or a plane or ground all these type of effects?

**Participant 5**

I actually haven't, but I've from time to time, asked about Heli’s and things like that. And so, I've never been on one with a child. Or you might probably already have interviewed some of them. So, I don't have any experience with that. There were kids that came through to our province. But they came on an ambulance. And I wondered why they didn't just get into a helicopter. Yeah. But I don't know all the ins and outs around that. But I know what you're talking about a four-hour ambulance journey, which for me sounded as if it was perhaps too long. I'm not at all familiar with the actual physiological changes, and I don't have experience.

**Researcher 1**

So, I think this is something that should be included in the education because I think something that comes through is just because you have a helicopter, or a plane doesn't mean it's in the best interest of that premature neonate to be in that noisy and vibrating machine if it's not a very well rated machine, if that makes sense. Yeah, so I think, then there also needs to be an understanding of the effects and what is best. Is it better to go for hours by road? Or can you do it quicker? But maybe the effects? Yeah, and then I guess the equipment needs to be covered, because it's not all the same as in ICU. And also, the environment in the back of the ambulance is also different. So, if you take an ICU sister, she might be very well skilled with the equipment that you put in the back of the ambulance that might be a bit different, because the equipment and the space and so on. I've heard someone before mention that. The hospital that is sending the neonate away for transfer, should be packaging the neonate? Do you think they know how to package a neonate in the ambulance nicely? Or in the incubator? Or is it a joint effort? Or how do you see that come into this education?

**Participant 5**

Yeah, I would think that it needs input from the actual paramedics themselves, because I think as you say, we don't always know how best to arrange everything, such that it is ideal to enter the ambulance and to be stable, but for the infant to be visible to be monitored, and all that sort of things. So, I think that would have to be if it was a nurse who was doing the course for example, then you definitely need to go through how you do it and what you're aiming for, and how best to put what we know, in order to be able to visualize, monitor and intervene if necessary without wasting too much time. But also, without compromising the infant you know, hypothermia and things like that. So probably a joint effort is what I'm imagining.

**Researcher 1**

Then I just want to touch on the ventilatory support for these special need’s neonates. Have you seen ambulances with advanced equipment like humidification and heated circuits and these type of things in state or does it not exist?

**Participant 5**

I haven't seen them but I wouldn't know whether or not they're actually exist. So, I'm afraid I would stand under correction. I think that's something that EMS guys would have to talk to. So, I don't really know what they have there.

**Researcher 1**

So, on the private side, I wouldn't say it's standard now. Only specialized vehicles. But humidification is present. So, you can take the same chamber and circuit over from ICU. Or you can start a new, heated, humidified circuit for that neonate. Depending on what the ICU is using. So, do you think that is very important? Or does it depend on the time that the neonate will be ventilated? Or how do you feel?

**Participant 5**

I think that's very important, you know, the newborn, especially the little ones. They have such a short trachea. So, any obstruction from secretions or drying out, can quite quickly affect your ability to move air. So, I actually think that it should be standard, to be honest. I mean, you know, even if you're going for 15 minutes, we sometimes say we did make do with what we have, but I don't think we should approach it that way. Yeah, I would say we should have standards that are to be expected. And we shouldn't go below that. And that's what we should be doing. And that's what we should be training on.

**Researcher 1**

Because something that was tough for me coming from private. We had the capability to take over CPAP with the same nasal masks and the heated circuits and so on. So, we don't change much for that neonate. And I think it would be much better for them and the outcome to just continue the care that was initiated. You would take a patient that appears to be quite comfortable on CPAP, and then we have to wait for the doctor to intubate for transfer. And that always seems like it is quite unnecessary. If we had the right equipment.

**Participant 5**

Absolutely. I would 100% agree with you. And I think sometimes, again, it's not so much a knowledge thing, or Oh, it's again, a resource discussion. And wouldn’t it be great if national provincial health departments were able to support the improvement of neonatal care by ensuring some of these standards in the public sector. It should go really together with their improvement in competencies and skills for paramedics.

**Researcher 1**

I agree. Because, we won't fix all the problems just with education. There are various things to address. But I think this type of ventilatory support methods should be included in this education because it might be new to some people. Let's say the guy's doing transfers in the state sector never seen it, but this might happen in future so it's important to understand everything. That's all the questions I've got from my side, I don't know, if you want to add anything else, or are you happy with what we discussed?

**Participant 5**

Yes, just the one thing. It might be covered under vascular access, but just the whole discussion about intra osseous infusions. Usually the neonate, you have the advantage of an umbilical cord. So, you, you get into the umbilical vein if you need to use as an emergency. But if for some reason, you know, that all doesn't work, or you need additional, I know, a resuss has got its own problems, and maybe with transportation, but it's just perhaps an additional skill. Whether it's in I don't know if it would have been covered in the vascular access section, but perhaps something just to remember can be done.

**Researcher 1**

I'm glad you mentioning that. Because I think, as you said, the emphasis would be on umbilical access. Or, you know, the teams would already have something in place, like peripheral or PICC lines and so on. But we don't really see neonates with interosseous access. And I think the general teaching for pre-hospital people is that should be reserved for emergency cases. And I think it tends to lean more towards adults, because I think, I don't know if it's the risk or the lack of exposure, but I think there often is quite a hesitancy to put a needle into a bone of a very small patient.

**Participant 5**

So, it's obviously not as easy. The risk is probably a little higher. So yeah, perhaps I suppose it's one of those background skills, should you find your back against the wall, and there really isn't anything but as you correctly say, probably the majority of cases, there's some kind of access that someone is managed to find.

**Researcher 1**

But you do find you go to remote settings, and then you do find an IV, but it's not patent and there's nobody around to assist you. And so, we've gone past the window of umbilical access. So then as an intervention, I agree, I think it needs to be definitely included as a skill and as an option because that is better than no access for a long transfer.

**Participant 5**

As long as the people as you correctly say have been properly trained.

**Researcher 1**

I'm going to stop the recording unless you wanted to add something else.

**Participant 5**

I think that's all I can think of at this point,
