# Supplementary material for: Developing a South African curriculum for education in neonatal critical care retrieval: An initial exploration
Source: PLoS One. 2023 Aug 31;18(8):e0290972. doi: 10.1371/journal.pone.0290972 (PMC10470938; doi:10.1371/journal.pone.0290972)
Supplement: S1 Data — (ZIP) [file pone.0290972.s002.zip › Data Compressed/Background reading student group [4].docx]

**Background reading**

**INITIATING THE DEVELOPMENT OF A SOUTH AFRICAN CURRICULUM FOR EDUCATION IN NEONATAL CRITICAL CARE TRANSFERS**

**Introduction**

I would firstly like to thank you for sparing the time to participate in the study “Initiating the development of a South African curriculum for education in neonatal critical care transfers”. Loosely, the transfer of neonates in South Africa is performed by advanced life support (ALS) providers. This high risk service is reserved for specialist teams internationally. Adverse events during these transfers have been associated with the providers’ level of knowledge. There is currently no specific course in neonatal critical care transfers offered in South Africa. The practitioners that fall under ALS providers have variable education backgrounds. There is no guidance from South African governing bodies on the methods and content of education in this specialised field. The purpose of these interviews are to establish your opinion on education in neonatal critical care transfers in South Africa.

To inform our discussion, please review the following information:

1. Experts in the field of neonatal critical care retrieval in South Africa were asked to give their opinion on what **learning objectives** should be included in a new neonatal critical care retrieval course curriculum.

| **Learning objectives** | |
| --- | --- |
| **Anatomy and physiology** | Neonatal specific |
| **CCR Systems** | \| Adverse events \| \| --- \| \| Appropriate receiving facility \| \| Escalation pathways \| \| Network limitations \| \| Patient criteria for transfer \| |
| **Conditions** | \| Conditions from study \| \| --- \| \| Congenital heart defects \| \| Infection \| \| Prematurity \| \| Respiratory:   - Bronchopneumonia - Diaphragmatic hernia - Meconium aspiration - Persistent pulmonary hypertension   Surgical emergencies   - Gastroschisis - NEC \| |
| **Continuity of care** | Feeding  Skincare |
| **Documentation** | Referral and handover specific |
| **Emergency Procedures** | Airway management  Chest decompression  Resuscitate |
| **Equipment** | Incubator  Ventilators  Infusion devices  Troubleshooting |
| **Indwelling attachments** | Colostomy bags |
| **Medication** | Neonatal CCR specific |
| **Pathophysiology** | Neonatal specific |
| **Patient assessment** | Neonatal assessment |
| **Patient monitoring** | ABG  ECG  ETCO2  Fluid balance  Glucose management  Perfusion  Thermal regulation |
| **Transport considerations** | Acceleration deceleration  Modes  Movement and sound  Patient packaging |
| **Vascular access** | Arterial Lines  Central Line  IO access  IV peripheral  Umbilical |
| **Ventilation** | Bag valve mask  CPAP  Heated circuits  Humidification  Neopuff  O2 Blending  Oscillation takeover |

1. Experts in the field of neonatal critical care retrieval in South Africa were asked to give their opinion on what the **duration** should be for such a neonatal critical care retrieval course.

| **Duration of education** | |
| --- | --- |
| **Suggestions by expert participants regarding duration** | - Continuous education - Determine outcomes before timeline - Introductory course (Lead and secondary members) - Master’s degree (Lead members) - Not short course - Part time - Post graduate diploma (Lead members) - Unknown duration |

1. Experts in the field of neonatal critical care retrieval in South Africa were asked to give their opinion on what the **method of education** should be for such a neonatal critical care retrieval course.

| **Method of education** | |
| --- | --- |
| **Methods of education** | Interactive  Mentorship  Online learning  Specialists discuss topics  Work-integrated learning and clinical placement   - CCRS vehicle - Control room - EC that receives neonates - NICU - Theatre |

1. Experts in the field of neonatal critical care retrieval in South Africa were asked to give their opinion on what the **method of assessment** should be for such a neonatal critical care retrieval course.

| **Method of assessment** | |
| --- | --- |
| **Methods of assessment** | \| Continuous assessment \| \| --- \| \| Discussions \| \| Oral assessment \| \| OSCE Skills assessment \| \| Portfolio of evidence \| \| Simulation \| \| Written assessment \| |
