# Supplementary material for: Developing a South African curriculum for education in neonatal critical care retrieval: An initial exploration
Source: PLoS One. 2023 Aug 31;18(8):e0290972. doi: 10.1371/journal.pone.0290972 (PMC10470938; doi:10.1371/journal.pone.0290972)
Supplement: S1 Data — (ZIP) [file pone.0290972.s002.zip › Data Compressed/Transcript 7 FGD Additions.docx]

**Neonatal Critical Care Transfer Education Curriculum Design FGD Transcript**

**Researcher 1**

By remaining in this chat, we assume that are giving us consent. Okay, so this focus group discussion will be exploring your opinion in neonatal critical care transfer education. So how does a focus group discussion work? The idea is that you guys will have a discussion with each other on the topic. And then we will facilitate this by asking questions. So please keep your microphone on mute, and then raise your hand if you want to say something. So, let's start with a quick introduction. All I need to know is what is your name? Where do you work? And what is your involvement with neonatal transfers?

**Student 1**

Morning, everyone. So, I am a CCA and based in Gauteng. I qualified in 2018. Interesting topic. I know there's a lot of uncertainty, especially for us ANTs. But hopefully, by the end of the session, most things will be clear.

**Student 2**

Good morning, everyone. I am an ANT paramedic working in Gauteng. I'm currently an ALS paramedic doing ICU transfers. And we do a lot of neonatal ICU transfers from our hospitals to Academic hospitals. I'm very active in terms of neonatal transfers.

**Student 3**

Good morning. I am an ECP based in Gauteng and I also do lots of neonatal ICU transfers.

**Student 4**

Good morning, everybody. I am based in Port Elizabeth in the state sector and involved in operations on the road. Neonatal transfers, I am an ECP. Also doing a lot of aeromedical transfers with neonates.

**Student 5**

Thank you very much. Good morning, everybody. I am a CCA, and I qualified in 2007. I'm currently working in Limpopo province. Here in Limpopo we have about seven hospitals, of which six of those are primary. And one is secondary. There's the one with about two three neonatal ICU beds. And the distance that we cover is about a range from 24 kilometers to about 140 kilometers. Then we have a tertiary hospital that is about 197 kilometers away from the base that I work from. But that's basically my involvement with a neonatal ICU transfer, I think.

**Student 6**

Good morning, I realize I'm probably the youngest here. I'm qualified as an ECP. With my BEMC in 2017. I've recently just left the road about a month ago now lecturing, however, I worked for a private company in the Western Cape. My involvement regarding neonatal transfers was limited. In our company, in the Western Cape, we have a dedicated neonatal transfer unit, on which I worked a few times. Not as often, mostly in a training capacity. But yes, that was more or less the extent of my exposure, and then some transfers whenever they were sick as well.

**Student 7**

I am an ECP ,currently an operational paramedic in Gauteng for the state sector . Not all the time in neonatal ICU transfers. So yeah, knowledge and experience not too limited. But yeah, not too much. So nice to meet you all.

**Researcher 1**

But the idea is that you guys are having a conversation with each other on this topic. So, we'll just jump in and prompt a little bit and try and guide the conversation. But yeah, so if you want to say something, you just raise your hand and then I will facilitate that. Okay. So, the first question I have for the group then would be with regards to how much time was spent during your education on neonatal transfers. So, I understand we had variable education backgrounds. But my question is, how much time was actually spent on preparing you for neonatal transfers? So, is there anybody that would like to give a comment on that?

**Student 7**

I'm sure if I remember correctly, ICU neonates, I think it was about six months, because I think we did half the year of neonates and we did half a year of ICU which was done in fourth year. So maybe five months or so. But we did pick up a little bit of the neonatal knowledge in 1st year, second year, third year, but not in depth. But you went more in depth when we were doing the neonate part, which was for about five months or so in fourth year.

**Researcher 1**

I just want to separate the two, you said the neonates and pediatrics. So, if we look at neonates by themselves, how much time is it still like six months in fourth year?

**Student 7**

I'd say basically, because they didn't actually separate it. It was pediatrics and neonates, but then obviously, the doses and how you treat them. So, if we doing cardiovascular, they wouldn't say okay, we focus in now only on neonates, we're doing cardiovascular, and the doses and then knowledge and everything, which is okay, in the neonate. This is how much you would give or this what you would do and stuff like that.

**Researcher 1**

Okay, great. So, you said it was a bit of a combined approach and it's more or less six months, mostly in fourth year.

**Student 7**

Yeah. About five or six months.

**Researcher 1**

Okay, great. Thank you very much. Anybody else who'd like to add to that their experience how much training they had?

**Student 6**

Thank you. I did my degree in the Western Cape. And regarding neonates, the way the curriculum works is that during first and second year you do anatomy and physiology and that included some neonatology, anatomy and physiology. And then you only really deal with neonates again in fourth year towards the second half of the year, where we did intensive neonatal ICU training, including medication dosages, pathology, ICU transfers, etc. And then we did what they called a high-fidelity simulation, including a neonatal transfer as well. And then throughout the fourth year, we did a lot of clinical shifts in the neonatal theatres, as well as ICU shifts. And yeah, that was the extent of university training. And then as I said before, I did some training shifts with the CCRs vehicle. And that was doing transfers with the crews at the time, I guess.

**Researcher 1**

Okay, thank you very much for that. The focus was in in your final year of study.

**Student 3**

So, in third year, you will be rostered onto a PRV and ICU ambulance. So, the first time we actually start doing the neonatal ICU transfers would be in third year. But then you only do things practically without really knowing your theory and everything. So, you get to see how the qualified practitioners do the ICU transfers, especially when it comes to the neonatal ICU transfers. And then we go into detail in our fourth year.

**Researcher 1**

Okay, thank you. So, what you're saying is you started seeing neonates in your third year as a student, but the bulk of the knowledge and the content was more covered in fourth year.

**Student 4**

I think most of the university curriculums were probably based on the same time tables for the BEMC program, I was just wondering if you were referring to education regarding neonates, just book knowledge, as opposed to the practical side of education? Because as you know, you never got taught any of the tricks on how to effectively manage a neonate during a transfer all the nitty gritty things you have to do. Like for instance, securing a tube a certain way. Do not make it pull in an incubator. So that's the other side of education. So that's what I was, might be asking what you were referring to us as when you asked about education?

**Researcher 1**

Thanks for that comment. So, it's nice to separate the two. So, I guess I'm asking both. On the programs that you guys did your education in both practical and theory, what was covered? So, I think what you're alluding to is that there was maybe not as much focus on the practical side and the tricks as you would say, on how to transport a neonate properly. Is that correct?

**Student 4**

Yes, most certainly. Yeah. For me, it's correct. The experience part only came after I qualified and had to do it by myself. I think most of the time, a lot of practitioners are very careful of allowing a student to manage a critical neonate, three days old baby with a lot of comorbidities, you know, and handing it over for a student to secure the tube or do that to do this. I think a lot of the guys are careful. To you allow anyone else but themselves to do it? But definitely, from a university perspective. At the university that did not adequately train us I feel in a way, you know, to manage the practical side of the transfer. Knowledge was fine, but the practical side I felt was lacking a bit.

**Researcher 1**

All right, thank you very much. Student 2, can you give us your side of the story? I know you've been qualified for some time. And just your side of how much training did you do in your day?

**Student 2**

Remember, I did the CCA program of which the program was about nine months. Now we have to cover the literature and the practical’s within that nine months. Now we didn't spend much of the time in terms of pediatrics and neonatology. We had to be exposed and the clinical practice to do Neonatal ICU. But we didn't cover much of how to manage a neonate in the ICU transfers. That was when the course was compressed for nine months. We didn't cover much. Like everyday transfers, it's a new learning curve for us as a CCA. Now, there was no much education in terms of neonatal transfer.

**Researcher 1**

So, I hear what you saying, it was a very intense course. And the limitation was time. So, if there was more time, I'm sure they could have spent more time on practical’s and so on, but what you saying is, there wasn't enough time really to cover neonates in depth, practical wise and theory.

**Student 2**

Absolutely. I think we spent something like a month on paediatrics.

**Student 1**

Yeah. Just to add. Remember, as CCA's most of the experience would come from the BLS faze and ILS faze. Then, I think in a way made things a bit easier. Dealing with the module though, the time was not as adequate as we would have liked. But considering the amount of time we spent on the road before we went to, to do the actual course it did help. And yes, like I said, it's always good to have additional knowledge and self-training in a way.

**Researcher 1**

So yes, we can appreciate that by the time you get to the CCA course you are already very experienced, and you've seen neonates and you've probably transported some. But unfortunately, the course was only nine months long. So, the time was limited. Is that correct?

**Student 1**

Absolutely.

**Researcher 1**

All right. Thank you. So, if I can ask you guys then. Were you adequately prepared to do neonatal transfers after you qualified? Were you ready? Or did you feel you were not ready? If somebody wants to add to this?

**Student 5**

Thank you. I just want to add some more on the preparation part. Like when I qualified in 2007, I was working for the municipality service. And it was quite different from the provincial service. They were the ones who were doing the planned patient transfers. So as the municipalities were not doing planned patient transfers. So, my experience was like, almost zero, from 2007 to 2010. When I resigned, then when I come to Limpopo, it was a whole different game, because now I was working for the provincial service. This is where things were happening. Although the experience was very limited. But when I come here, I started working with doctors, pediatricians, and they started to push this load of work, and experienced that to grow from there. In class, it was an issue of learning anatomy and physiology, when we do the OSCES, patient simulation, and when we go to road pracs. That's where we get lots of experience as a practical part. But other than that, I got my experience when I started working in Limpopo in 2010.

**Researcher 1**

Okay, thank you very much. I think it's a very important comment that you are making. I think there's definitely been a change in the industry, you've been qualified for a very long time. And in the beginning, you're saying that moving of neonates or planned patient transfers was not so much present at the time. And then when you came back to Limpopo then you started getting exposure to moving neonates. And we also can appreciate that the industry has changed and then you saying that you gained a lot of experience then from doing neonatal transfers, is that correct?

**Student 5**

That is correct. Yes. Thank you.

**Student 7**

I just like to add on the preparation side. I remember on my third year, I only did one neonatal ICU shifts, which I didn’t even get a patient on that day. So, but I do remember doing one or two shifts when I was working in private sector. Where I had to assist in the neonatal side of transferring. So, when it comes to preparing me in terms of the practical sides, it was a bit limited. But I also could understand in a way from the university side that at that time, maybe they couldn't get the students enough ICU shifts to be focused mainly on neonates and stuff. But when it comes to the knowledge itself, looking at the time that we do spend with these patients, during our transfers, obviously, depending on the area that you work at the province that you will, because we know other provinces, they spend quite a lot of time when transferring these patients. But looking at the time that I have to spend with a patient, I feel like the university did pull through in like preparing me on the basics that I need to, to know about, about transferring a neonate or treating a neonate patient. And I think, for me, I felt like they did their part. Obviously, we learn every day, you will learn something from university, and will go to the actual patient, and you'll find that it doesn't really work. Like the book says it should work. But you learn every day, when it comes to how to handle the patient you learn every day because you handle a patient in a certain way, when you transferring a patient when it comes to the tube securing or the other attachments with a patient. And you find another patient where you actually need to do it a little bit more differently than your previous patient. But on the preparation side, I felt like we could improve on maybe increasing their knowledge a bit because things are changing. But also, that's a practitioner to practitioner's responsibility to update themselves and always learn new stuff. But yeah, there could maybe be a bit of improvement when it comes to preparing us. But I felt like the knowledge that I attained from the university when I was doing my block was quite sufficient. It really did help a lot.

**Student 6**

And I think it feels similar to student 7. But I think we also have to distinguish between confidence and knowledge learned on the road by experience. And then book knowledge and practical knowledge and OSCEs obtained in class. So, when referring to knowledge learned in class, I think theoretically, we were very well prepared. I was fortunate in that one of the critical care transfer lecturers, had joined in my fourth year, so we've managed to get information on the nitty gritties. Like, how to fasten a tube correctly when you move a patient. When we did our high-fidelity sims, we were also exposed to how to move a neonatal ICU patients. So, we were very much aware of those things. On experiential shifts, I also worked some ICU transfer shifts. However, it was just luck of the draw, I had no patients. There were other members in my class that had multiple neonatal transfers. Whereas I got more experienced learning in the neonatal ICU shifts where the sisters were really kind and I gave you a baby for a week to look after and you would feed the baby, clean the baby to all those things with the nurses. So, however, qualifying and then working on the road, you see minimal neonatal transfers in the private sector, just because of medical legal risks, and they have a specialized unit. The confidence then dwindles the longer you go without having seen one. So yes, if I had done neonatal transfers pretty soon after qualifying more often I would have probably had a bigger confidence level doing them. However, the way some private companies operate in the Western Cape, that confidence dwindled over time because of lack of exposure.

**Researcher 1**

So, what I'm hearing across the group is that there was quite a bit of theory preparation for neonatal transfers. But because it's such a specialist service, there were not that many neonatal transfers going around as a student to get exposure. So, my next question to you guys then would be, how did you other than exposure of neonatal transfers? How did you cover any knowledge gaps that you felt you had? Did you guys do anything to add to your knowledge as you went along? Or do you feel that there wasn't a gap from theory, it was only the practical side of things?

**Student 7**

For me personally, I'd say the knowledge that I got, I felt was sufficient. But obviously you will get a patient where you feel like it's something that you want to read up on. Maybe they did cover it in university, but you may be forgot, or maybe they did miss that part, depending on the kind of patient that you have. Like journals would help. Go into the internet and read about that patient and what they had and how to manage them better in future if you do come across that kind of patients because they can't teach us all the things. And when you do come across as a practitioner, something that you feel like you could improve on or you didn't really understand it, you just winged it. I find that reading up on it in journals did help on my side.

**Researcher 1**

Okay, thank you very much for that. So, you said you covered it, by reading journals, and so on. So, my next question to you guys then would be, do you think there is a need for additional training in this area? Do you think people would be interested? Is there a need? Do people need to go for additional training?

**Student 3**

Most definitely. Let me tell you about my experience. I remember doing my first neonatal ICU transfer. It was really nerve wracking because now you don't have anyone to rely on. If you could get a course and do it part time. That will make sure that when it comes to practice, we are really solid.

**Researcher 1**

What I heard was you saying yes, you think there should be additional training because you were a bit out of your depth with your first transfer? Was there anybody else that want to give their opinion?

**Student 1**

Oh yes, I think there's a need, knowing that the medical field evolves every time so there's definitely a need to always get more knowledge and training on the subjects.

**Student 6**

I do think even having had six-month training in neonates that there is definitely a need for a secondary program or more focused program for neonatal critical care. Because even within the BEMC things felt rushed, there were definitely topics that we could have gone in more depth or spend more time with. It was definitely a case of trying to meet deadlines and targets more so than having a sole focus on neonatal critical care. And a lot of the learning that took place was also reliant on what you learned during your experiential shifts. So, I do think there will be a benefit of a more focused neonatal critical care course. What you call a curriculum as well, which includes experiential learning shifts in as well.

**Researcher 1**

So, if I can put the question to the group then, if we think there is a need for additional training, what areas do we think would need additional education? So, when you started doing your transfers, you started seeing your first neonates. What areas do you think other than just physically touching the neonate was lacking for you all that should be focused on if there is additional education?

**Student 5**

Thank you. Yeah. I believe so. You know, when you start working with this little ones you start to experience things that you will never experience in class and all the practical stages. Like for example, I worked for 10 years in Gauteng and never came across a case of gastroschisis. And when I came to this side, it's like around every corner, there is a case of gastroschisis that I had to deal with. And it is the first time that I'm experiencing this kind of patient. I only know that if I have a patient with protruding intestines, I had to keep them warm. But other than that, I had to just treat the patient as I see fit. So, with this little ones, it's a list of treatment that I need to concentrate on, I had to make sure that I cover those intestines with the clean, sterile plastic, I had to make sure that the fluids balanced. Prevent contamination, infection, all those things. So, I think if we have some sort of a curriculum that deals with all this kind of diseases and surgical problems,

**Researcher 1**

You are very correct. There's a lot of specialist conditions that would need to be covered that maybe was not covered, that you are alluding to. So, are you saying that it's the disease process that you need a better understanding about? Or how to approach the patient better? Or what do you think was lacking? Did you know about the disease or did you just not know how to manage the condition?

**Student 5**

Thank you again. I didn't know about the disease and never came across it in class. The only first time I came across it is when I'm doing the neonatal transfer from one hospital to another. So, the whole assessment processes and the treatment and management of disease processes, I think it's very important. The curriculums will include the assessment and management of disease processes. Thank you.

**Researcher 1**

Thank you very much. So, you saying that it's both the disease and the approach. I just want to quickly touch on something. How do you see experiential learning being done, postgraduate? How do you think this should be approached?

**Student 6**

And I think it would be beneficial to approach it more in a block formation. So, do a block of shifts. If it's part time versus full time, I would imagine a part time course would be more beneficial considering people attending would be all qualified working practitioners. However, there would have to be a certain number of experiential shifts, either guided or not guided by neonatal professionals. But yeah, blocks or just slotted into which ever days the practitioners have available, and then preferably in hospital, more so than on the road. But some road experience as well, the road experience will have to be on these critical care transport units. Otherwise, they would just be a waste of time.

**Student 7**

What could we improve on or attain more knowledge on? if I come across a patient, maybe a baby that's been transported to home after birth, and they get involved in accidents and stuff like that. Trauma was not something that was deeply dealt with when it comes to these small little babies. We understand. They're small, and they are fragile. But looking at your equipment as well, you have very limited with treating trauma patients that are that tiny. So, I'm just thinking maybe trauma is something that we could maybe increase on.

**Student 2**

I just want to add something, you know, I was listening to the colleagues here. They did cover six months of your learning in neonates. For CCA, the whole theoretical part of the course was six months and the rest were clinical practice. I recommend additional training in neonatal transfers. Remember, as CCAs we were treated as jack of all trades, we have to play a role of the cardiologist to cardiac patient, you have to play a role of a gynecologist when you get these types of a call of a breech presentation or whatsoever. I personally recommend that you should give us a training program for this. We need a neonatal transfer course. We did cover some congenital heart defects and diseases but like with every day ICU transfers you get something like the Poland syndrome, sometimes you will be asked that you have to give them a Thomson score. That was something that I had to go and read about and every time when you arrive at other facilities, they assume that we will be waiting for you to come in do some other management before you can transfer patient. We really need this kind of a training to protect us also, you know, all the adverse events during this long-distance transfers.

**Researcher 1**

Thank you very much. So, I what I'm hearing is that you're in agreement with that there are a lot of specialist conditions that are not covered, and additional education and those areas, which would be great.

**Student 4**

Yes, I think it's all valid points. For me, on my side, I would just like to say that I think it depends on where you are based and how you're qualified. And also, I know for us in the government sector, quality assurance isn't existing at the moment. So, if you are going to conduct a case, and there are no quality assurance measures in place, there is not going to be anyone or any repercussions for maybe, you know, doing a wrong thing unintentionally. And also, good criticism to help you go forward and understand more. So that's the first aspect for us. There is no accountability really, given from my perspective, if a neonate passes away, due to malpractice, but unintentional malpractice, not because you know, intentionally doing harm. And then it also refers to this profession of pre-hospital EMS not having any sort of internship program post qualification or com-serve, you know, where as you are placed with experienced practitioners for a year, or maybe two years, before you are allowed to make these important decisions on your own. Because for me, personally, I do feel that a lot of the neonatal side effects caused by practitioners are due to unintentional lack of experience, or skill. And we often just put that to the sideline and blame that there are not enough healthcare workers or not enough people with the training to do the calls. So yeah, that's all I would like to add to that.

**Researcher 1**

Thank you very much for that comment. Would it be fair then to say that if there is a lack of quality assurance, to have some content on that? Of what quality assurance is in an education program like this?

**Student 4**

Definitely, I do think so. But if quality assurance isn't existing with any patient at all, I don't think you know, just trying to implement that for the neonate side will be effective. I think it's a, it's a broader issue that needs to be addressed. And also, that will also help the practitioners in SA to actively, you know, enroll in involvement with training purposes. And not only when the HPCSA is sending you an email, that you're going to be audited, that you then all of a sudden start looking for CPD points. So, I do think it's, it's very important. Yes.

**Researcher 1**

Thank you. You also touched on mentorship? Do you think mentorship should be introduced in this form of education, someone that will coach you and guide you, and then maybe when you're done with the education will stay with you, assist them stay in touch?

**Student 4**

Yeah, for me, personally, when I qualified. We were fortunate enough to have experienced practitioners around us. So, I remember working for one month with one of my colleagues when I entered the government sector out of school. So that really helped me a lot to understand the system better. Although I had knowledge from treating patients, you know, understanding the system and our how to approach people from different perspectives. That surely helped me so much. So definitely, I felt there was a lot of value in in having a mentor for that month after qualifying.

**Researcher 1**

So, if we had to put a timeframe on this type of education after you qualify, how long do we think would be a fair amount of time to spend on this type of education? I know it’s difficult to do, just estimate or think but in your mind, how long do you think this type of training would be? Or should be?

**Student 4**

I would like to add, I think that neonatal care transfers should be a selective thing in the EMS. Although we cannot afford it right now with a lack of people and you know, finances in SA to do it, but as in any other specialist environment, like doctors, I think it should be a selective thing. And also, you would get practitioners that will tell you upfront that they are not comfortable, and they do not really want to deal with neonates. And we should be able to, I think respect that. Although you have to know the basics about it. It should be a selective thing for the practitioners that feel that they would like to do that on a more constant basis.

**Student 5**

Um, in my opinion, I believe that this kind of course, will be in two phases. It should be as a standalone course for a specialized team. So, for people like me, myself, I just do neonatal transfers whenever they available. So, if we have our kind of, maybe a year course, that we can just do neonatal transfers. And then there'll be another course for specialized teams. Maybe three years, four years degree, I think that will make a difference.

**Student 3**

I think if we could be granted an opportunity to go to specialist neonatal hospitals so we could also be hands on, on how a receiving facility would like or prefer the patients to be treated. I think we could learn a lot from that.

**Student 6**

I do think that possibly considering a yearlong post-grad course would be beneficial. I do agree with others that half of the course should be focused on experiential learning. So again, to neonatal ICU units. I do recall in our BEMC, we worked in the NICU however, we are lucky to have those facilities in the Western Cape. So, it would also be location dependent, obviously. And then the experiential learning section could also include working in the neonatal specialized units. So again, in Western Cape government has a NICU bus that only do pediatrics and neonatal transfers. And then private, we have the dedicated CCR teams, that runs, predominantly neonatal transfers, and then some of the more critical adult transfers as well. However, I do think that there should be some theory component to it, as well as simulation, OSCE training with regards to neonatal resuscitation, how to use the ventilator, ventilation settings. And those things as well. Just because there is definitely a difference in the education having been on BEMC. And then the gaps that need to be covered, having had the short courses previously. So, I think just to level out knowledge across the board, it would be beneficial to have a theory component with practical’s and in class assessments and then have experiential component afterwards.

**Student 7**

Yes, I think maybe I do agree about maybe going about a year or longer. Because we did about six months, though I still feel that we were adequately prepared. Because it also depends on practitioner to practitioner as well as to what you want to specialize in. When it comes to when you're on the road and you are independent. So, there are those people that focus solely and only do ICU and neonatal transfers, for those kinds of people, then maybe we could try to implement, like a specialized neonatal course. We will go in depth when it comes to neonatal treatment and the pathologies and conditions and all those things. Yeah. But for people like me, that's not really focused entirely on neonatal transfers. Yes, there is improvement that minimally so as to the increase in the knowledge that I would maybe try and attain because I'm not only focused on neonates, I also deal with adult patients. Other people that will rather do the rescue side. But you can also have like a program, which is specialized for those people that are really passionate about it. And yeah, just to increase their knowledge on it, and so on and so on.

**Researcher 1**

So, what I'm hearing from the group is that there's quite a focus on experiential learning and exposure. And then also theory components should be covered. What I'm hearing duration wise is a year or longer. So that shouldn't be like a couple of weeks and just a short course. Is there anybody with an opinion with regards to whether we should bring online or in class or only practical’s in hospital? Or what format should this education be in?

**Student 7**

Adding on what I did say previously, it will depend from practitioner to practitioner, but online or maybe a part time course for those people that are still working operationally and would like to increase their knowledge when it comes to neonatal specialized units, so maybe a part time course where they will be able to go in or and also have online classes, if they can't go in at school, maybe they still work and stuff like that. And they will be doing that course to go in depth more when it comes to learning more information about it. So, I do think actually part time where you go in facility when learning and online courses, and they should also try and get them to get more involved on the practical side, when it comes to that, on the practical side and learning different ways on how to handle the different patients and the different pathologies.

**Researcher 1**

What you're saying is that online teaching would work well. And on a part time basis, seeing as people are working today. In our South African context, is online learning practical for everybody? Or do we see some barriers that might be in the way?

**Student 5**

Thank you. Um, I believe it's possible. Myself, I'm involved in an online class. So, if I am doing it, they can do it. But I also believe that this kind of learning should require some practical involvement. So, we need to do some simulation and stuff. But yeah, we can do this online. I agree with. Thank you.

**Researcher 1**

If we look at the financial aspect of this kind of education, do you think paying for this type of course, is a problem? Whether it be yourself or a grant or employers support? Do you guys think this type of education will be paid for?

**Student 7**

If you want to focus more and specialize on the neonate. And you can Yeah, try and pay for yourself. But from my side, I feel like employer support to be very beneficial for our patients. And for us, as well, to make sure that we do the right things when we treat this kind of patients. So, it depends, actually, it depends. Employee support would be wonderful. But if it's something that you personally feel like you will be doing, you can also try and pay for yourself. Thanks.

**Student 5**

The employer support is the key. It's just that different employers deal with this kind of issue differently. Some employers, they will require you to pay for your tuition, and then when you graduate, they will refund you the certain amount of money that you paid. Some, they pay the full amount and they will give you your monthly salary. So yeah, I think if they support us on this, we will enjoy going to school.

**Student 2**

Yes, I think it's imperative for employer to support this kind of programs, because it’s going to be beneficial for both us as well. practitioners and their employer. Number one, it improves patient care. And number two it will reduce the number of litigations and adverse events during long distance transfers of this patient. Definitely, the employer should support this kind of course.

**Researcher 1**

So, what I'm hearing from the group is that employer support and payment would be great, it would be in your benefit and theirs. Tell me, do you guys think people have access to computers, internet? And if there are contact sessions, will they be able to travel?

**Student 7**

Just on internet. South Africa is not that advanced. But I do feel that we are fairly getting there when it comes to technology and stuff. People, kids in the rural areas have tablets, we have cell phones, like I'm currently using my cell phone. So yeah, you have cell phones and stuff like that. And I do know universities, they have programs where there are students that can be live inside a lecture room while you at home. So, you can see what's happening in the lecture rooms, while the lecturer is lecturing, take notes record, if you cannot be present at home while the lecture is going on at a university, you can just pre-record the timings of what time the lecture starts. And when it ends, and stuff like that. So, it might work. I know it costs about R3000 to have that set up in time for you, at home. And in your laptops as well. So yeah, I think we actually are getting there I mean, if it's something that you really want to invest in your own knowledge. Yeah, something that could be done, honestly from my side.

**Researcher 1**

So yeah, I think because in 2021, and because of COVID, it's more realistic to be in online learning. I sent you guys a pre-reading document where we had interviews with experts in the field. And they gave us topics that should be in a curriculum like this, this type of education. Is there anybody that read through it? Do we agree with what they said? Do you think something should be added?

**Student 6**

I did read through it. I agree mostly with the document. I saw recommendations for it being a Master's. I think that should be an optional post-grad. I think having a course like this only be available to those wanting to do masters is not fair, to be honest. And then I didn't see any mention of aero-medical neonatal transfers, I think that should just be included as well.

**Researcher 1**

Okay, thank you very much for that. So, you are saying aeromedical transfers should be covered in content. And then you were saying that maybe to have it only as a Master's is too long?

**Student 6**

So, what I'm saying is I see it was suggested to have a neonatal program offered as masters. I disagree with that. I think some form of post grad diploma would be suffice. Only just because having a two-year Master's is not attainable for everybody. And this is clearly a need within South Africa.

**Researcher 1**

Okay, thank you very much. You are touching on a very important point. Because we need to ensure that this type of education is inclusive. So, what you're saying is that if it was a master's program, it would not be attainable for everybody. And a postgraduate diploma. So, let's say one year would be more attainable, is that correct?

**Student 6**

That is correct. The Masters will be too in depth and require a thesis and all those things. And it's rather taxing, especially if you have a family and you're working. But it's not as easy to do. And it puts a lot of practitioners off just because of the amount of work it requires. Whereas a post-grad diploma, is shorter in duration, and it can be done part time as well, which makes it more attainable and more easily to do versus a Master's.

**Researcher 1**

I just want to touch on the topic of methods of assessment, like simulations and written tests, and oral exams and so on. In your guys experience, the method of assessment. What do you guys think is a good approach? What do you think works and really test your knowledge?

**Student 1**

Oh, thank you, sir. I think a more physical approach your OSCE skills, your actual simulation could prove to show if the knowledge was impacted, and if you can do the actual work. So, more focus on the on the on the actual physical skills, I think would suffice.

**Researcher 1**

In your experience, do you think simulations, oral exams also have value? Or do you think the focus should be on practical assessments? In your experience? What do you think works?

**Student 3**

I think we can, we can begin with written assessments. And then from there, we can move on to your simulations. And that way, we can see that whether one can actually take what they know in theory and actually put it into practice with when it comes to simulations.

**Student 7**

When it comes to the assessment, yes. OSCES, written, and obviously it's it assesses your thinking and the knowledge part of it. And then the OSCES and the practical’s, how you handle the patient, how you treat the patient and stuff like that. But I just remember when we were doing ICU, it was the first time we had the assessment, then physically took us to the hospital. They choose a patient and you do the actual physical assessment on the patient. And you like, they ask you questions and you talk to what you would do what your next step would do. And they physically looking at how you are handling this patient. It's quite a risky thing. But if we are learning and it might help, then maybe that could also help because having a doll and a physical patient, it's similar, but it's not. The touch is, is very different. So, when you're assessing the skin, it's, it's a very different feel of how they, how you are treating the patient. So maybe trying to implement some of that, especially when it comes to ICU transfers with these small kids on how you're going to move your baby into the incubator, and stuff like that.

**Student 4**

I think, for me, most importantly, working with students. So, we've been working with the university students for a few years now. And I do think that, especially from a university perspective, that they should engage with pre-hospital practitioners more, and maybe employ or contract like honorary lecturers, or people that can give feedback regarding the students or to the university. In our setting, there is not a lot of lecturers from the university that do work on the road. So, they rely a lot on the pre-hospital practitioners to guide the students on the practical shift. So, I think students should be evaluated daily, while working and not only in an assessment where the cameras are switched on, and it's a controlled environment. And they are stressing obviously, because of the, you know, just the nature of their assessment. And the second part of it is I do think they should be more clinical reasoning assessments so that that can maybe be done in form of an oral assessment, a student's capability of reasoning, different topics with regards to treatment, and also problem solving. If a problem arises. And I think that's one aspect that should be focused on more and it would also be beneficial to simulate students to think more about, you know what to do in adverse scenarios.

**Researcher 1**

Thank you very much for that. So, what I'm hearing from the group is that there should be a hybrid approach to assessment, where it's a combination of oral assessment, but it can be done at the same time as touching a real patient. But there will be some form of mentorship or oversight to reduce risk. And then also, your clinical reasoning can be assessed nicely with oral exams, because there's a higher level of thinking involved. And then there's also a place for a written assessment to cover the basic theory. What I'm hearing from the group is that there's a nice mix of all the methods of assessment. You mentioned that continuous assessment and mentorship, and these are all important aspects of assessment.

**Researcher 2**

The question that I have, and if you would allow me to indulge and be a little bit controversial for a moment is we spoke earlier about the point of inclusivity and to make sure that this course is accessible, and understanding that what are we really trying to achieve with this? Yes, its self-actualization of providers but, mostly we're doing it for patient care. And I was wondering if I could get some feedback, or just some comments from all of you, as it relates to changes in scope of practice with regards to the transfer of neonates, number one, and number two, relating to this course, and how the suggested course will be accessible to many different cadres of provider, but also, at the same time fitting into a particular type of educational framework and scope of practice framework. So, two parts, number one, experiences with regards to the change of scope of practice, and neonatal care. And secondly, how can we make this course accessible to a variety of different cadres?

**Student 7**

Yes, firstly, there is a change in this scope of practice with regards to the new CPGs. We ECPs are the ones that are allowed to be transporting, according to CPGs, to be transporting your neonates, which does put a bit of strain. We have a huge number of patients that we need to transport and it's all our responsibility. And this would be very beneficial, if we do have a course. Because if the HPCSA feels that other qualifications do not qualify, or whatever they think, then this program of dealing with your needs and transferring these kinds of patients would be very beneficial to their patients, firstly, and to the whole system entirely, because now we have more people that are more knowledgeable with these kind of patients and will be able to help them during the transfer because it does put a strain because it's sometimes to delay the calls. Where we have an emergency transfer for a neonate. But the practitioner that's on shift cannot be doing the transfer, they need to wait for a night shift, or they need to call someone from another area. And that patient needs to go to theater. Quick, quick. So yeah, I believe there's quite a big room and the scope of practice and the new CPGs. It has affected us. And that way, if we do have the program, we'll be able to include everyone to be able to help us when it comes to transferring and treating this kind of patients.

**Researcher 1**

All right. Thank you very much for that. So, what we're saying is that, due to the change in CPGs, there's a lot of strain on the system, because advanced life support providers are a very scarce resource. And if we did additional education, and we could increase the scope of everybody, then it would help a lot.

**Student 6**

Yes, I agree in that what we have currently in the Western Cape a situation where we have experienced practitioners that have been transporting neonates for years and now all of a sudden aren't allowed to just based on qualification and the constraints they are that HPCSA is now released. And we haven't gotten to the point yet where we have a bridging course for them to now be allowed to do those transfers again. So, what we have is a situation of new practitioners that are allowed to do these transfers with minimal experience. And then old practitioners that now are no longer allowed to do the transfers, but are required to work and do not have the time for mentorship. So, I think a course like this, if then allowed afterwards for the CCA and the short courses to have access to, those qualifications where they can now do these transfers would be such a huge help. Just because, we have a case where in private we have four ALS on a shift at a time and it will most likely be you and only you that can do these transfers which does then put a strain on and also results in a backlog of transfers continuously. I've spoken to some of the government ICU vehicle crews and they say it's the exact same with them, where calls are pending from the morning already, just because they're the only ones that can do these calls. So, provided that we do allow short courses in, then allowed to afterwards perform these transfers and skills, that would be such a great help to bridge that gap that we now have.

**Researcher 1**

So, what I'm hearing is that, to make this inclusive, we include all scopes of advanced life support providers, and then the scope should increase so that we can be able to transfer more neonates because it is a problem.

**Student 5**

Yes, it is, indeed, a huge problem. In my district, it is about 25,597 kilometers square. And we are only three paramedics. We are all CCAs with no ECPs. So, we have four shifts, we work a four-shift system. There is one that doesn't have a paramedic. We rely on whoever is working after hours. But without the ability of our inclusivity of this type of practice of performing or doing the neonatal transfers, we are in big trouble this side. I understand that they are pushing so hard in universities to produce extra ECPs. But I doubt it's going to be enough. So, if there is a way to come up with maybe paths this course, this neonatal critical care retrieval course, if maybe there's a way that we can be included in that course, and we have that scope of practice, then we think it could be helpful. We'll see in the future, what will happen. Thank you.

**Researcher 1**

Thank you very much for that. We can appreciate the distances for these patients and the scarce resource that ALS providers are in South Africa, especially in your province. So, we appreciate that input.

**Additions from email correspondence post FGD on clarity regarding expert opinions on curriculum design presented as pre-reading:**

**Student 6**

Good day

Please find considerations below :

Conditions :

- HIE

- Trauma

Continuity of care:

- warming (kangaroo care etc)

Equipment :

- humidifier

- neopuff

Transport considerations :

- aeromedical

- charging stations

Hope these are useful.

**Student 7**

Agree with content presented

Would say that a Master’s level should be an option only for practitioners that want to specialize in Neonatal critical care retrieval.

**Student 1**

Evening

Please find below the feedback about our teams meeting as requested. Hope this helps.

Duration of education

- A continuous education is welcomed, offered to the broader field inclusive of all ALS practitioners

 Method of education

- An emphasis on mentorship and work intergrated learning and clinical placement

Method of assessment

- A more practical approach including discussion and OSCE Skills assessment.

**Student 3**

Learning objectives

Anatomy and physiology: Neonatal specific.

This should be included. I think it is very important, however, what can be included in this is the various birth defects that neonates may have. Whether common or rare.

CCR Systems: Adverse events, appropriate receiving facility, escalation pathways, network limitations, patient criteria for transfer

This should be included. I strongly agree with this.

Conditions: Conditions from study, congenital heart defects, infections, prematurity, respiratory, surgical emergencies

This should be included.

Continuity of care: feeding, skincare

Documentation: referral and handover specific.

This should be included.

Emergency procedures: airway management, chest decompression, resuscitate.

This should be included.

Equipment: incubator, ventilators, infusion devices, troubleshooting.

This should be included.

Indwelling attachments: colostomy bags.

This should be included.

Medication: neonatal CCR specific

This should be included.

Pathophysiology: neonatal specific

This should be included.

Patient assessment: neonatal assessment

This should be included.

Patient monitoring: ABG, ECG, ETCO2, fluid balance, glucose management, perfusion, thermal regulation

This should be included.

Transport considerations: acceleration deceleration, modes, movement and sound, patient packaging

This should be included.

Vascular access: Arterial lines, central line, IO access, IV peripheral, umbilical

This should be included.

Ventilation: bag valve mask, CPAP, heated circuits, humidification, neopuff, O2 blending, oscillation takeover.

This should be included.

2. Duration of course

It should be one year part-time course. It should be a post graduate diploma and not a Master’s degree as the ANTs do not qualify to do the Master’s degree.

3. Method of education

It should be interactive, mentorship, online learning, specialists discuss topics, WIL and clinical placement at mother and child hospitals such as Nelson Mandela.

4. Method of assessment

It should include the following:

· Continuous assessment

· Discussions

· Oral assessments

· OSCE skills assessment

· Portfolio of evidence

· Simulations

· Written assessments

**Student 2**

Good evening

I've just read through the draft and the experts advises. Its clear and straight to the point, I'm in full agreement the expert's advice. It covers exactly what we discussed in our teams discussion last week Friday. I, personally accept their recommendations.

However, I'm bit lost regarding the prerequisite or should I say requirements for the program. I noticed that it should be available for masters graduates(leading) and for post graduates . What about CCA paramedics for such an educational program.

I'm happy with the program contents, duration of the program, method of training and assessment.

I would really appreciate if there will be some consideration for CCA paramedics. It’s a necessary educational opportunity for us as ALS paramedics for personal clinical development and improve patient care. Some of us still wanna do more to our patients and utilize our full talents and potential in line with clinical evidence based researches.

I really appreciate the opportunity to be part of this research and how you kept me up to date . This has helped me so much in how I see and my clinical judgment especially with this neonatal critical care retrievals.

I conclude my response by saying it's absolutely true that our level of education in NICU is inadequate.

I hope you'll find this response in order.
