# Supplementary material for: Developing a South African curriculum for education in neonatal critical care retrieval: An initial exploration
Source: PLoS One. 2023 Aug 31;18(8):e0290972. doi: 10.1371/journal.pone.0290972 (PMC10470938; doi:10.1371/journal.pone.0290972)
Supplement: S1 Data — (ZIP) [file pone.0290972.s002.zip › Data Compressed/Transcript 2.docx]

**Interview 2**

**Researcher 1**

So, did you receive the consent document and did you read over it?

**Participant 2**

Yes.

**Researcher 1**

Okay. So just to summarize that, so the interview is voluntary and you can withdraw at any time. And then if there's any personal information, it will be anonymized during the transcription phase. Are you okay with it?

**Participant 2**

Yes.

**Researcher 1**

All right, then just a just a quick background on this study. So, the transfer of neonates in South Africa is performed by advanced life support providers. This is a high-risk service and it's usually performed by specialized teams internationally. And then adverse events during these transfers have been linked to the provider's level of knowledge. South African advanced life support providers, they have a variable education background. Then there's no guidance from South African governing bodies on the method and content of education in this specialized field. That's just an overview of the study and the background to do it. Today, I'm after your opinion on education in neonatal critical care retrieval transfers, and there are no right and wrong answers. So just to start off, can you please give me a little bit of your background, your education and where you currently work? And more specifically, your background in neonatal critical care transfers?

**Participant 2**

Yeah, sure. So, I trained in the UK, as a medical doctor, and then specialized in pediatrics. So, in the UK, that's an eight-year full time training program, which includes two years of neonatal intensive care training. So that's working in tertiary neonatal units. And the way that neonatal transport works in the UK is that we have retrieval networks, where the intensive care units provide a transport service, which is actually more like a mobile Intensive Care Unit, as it was. So, the services are run by intensive care doctors and nurses that go out in a dedicated ambulance to the district general hospitals, to help the teams in their local hospitals stabilize the neonates and then transfer them to the tertiary unit. So, it's very different to the way that transport is done in South Africa for neonates. And that would be my background really is working for those neonatal retrieval services. As a neonatal intensive care doctor. I have done various transport courses. Lester used to run a very good one, I think you mentioned in your reading material, BMJ Peter Berry textbook that used to actually be a book that accompany to a week-long transport course in Leicester which no longer runs now. But it used to be a practical course, that accompanied that text, which was my initiation into transport medicine that, really, I would say my training has been working practically for those retrieval teams. And then coming to South Africa, I only came to South Africa in 2014 as an emergency medicine fellow, so I have an interest in critical care and emergency medicine for children in the NHS. And so, I came through a year as a fellow and then I went back to the UK and then I've been here on and off, working since 2017. Working at a district General Hospital. It's been part time work over the last few years for them. Like I've only really been involved in a handful of actual transports here because, of course doctors aren't actually part of the transport service not pediatric doctors anyway. So, I've actually only been in the ambulance with a few babies locally. And that's when I particularly wanted to go with them. So that would be my background.

**Researcher 1**

Okay, thank you. So that's obviously a very impressive background in neonatal education. So, when you started doing the transport of neonates, did you feel that any of your education in neonatology had any gaps or barriers towards doing the transfers? Were there any gaps that you needed to close?

**Participant 2**

For me? Yes, I think as a doctor, and this is where paramedics certainly have the upper hand on the doctors would be the equipment usage. So, I really had to learn a lot about how all the machines worked and, you know, in a transport situation, I'm sure the paramedics are so used to that, being fundamental part of their knowledge, but for doctors, you know, I mean, especially British doctors, some, my colleagues don't even know how an infusion pump works. So, obviously, in a transport, you need to know the ins and outs of all your equipment. And that was probably my biggest learning curve for me, when I started doing neonatal transport, I think that the patients, as I said, it's really an extension of the, of the treatment provided in intensive care units in the UK. So as far as managing the actual babies, for me, the medicine the physiology was not too much of a challenge, but the equipment and obviously being in that ambulance environment, and safety considerations of movement within the ambulance environment. Those things were new to me, and important to really understand.

**Researcher 1**

Okay, thanks. So just to quickly summarize the section. So, you did your training in the UK as a medical doctor first, and then you specialized in pediatrics, which included two years neonatal education. And then you did some of your experience on neonatal transfers was on the transport network, which is quite different to what we do here in South Africa. You guys have doctors and nurses, you go out to clinics or smaller hospitals and stabilizing transfer these patients. You said you also did a transport course. And the book was one of the resources that I mentioned, but it's linked to a course in the UK. And then in 2014, you started working in South Africa, in emergency medicine, and your interest is children and pediatrics and neonates. And then from 2017 you've been doing a part time I work in a general hospital. You've done a couple local transfers. Can you just maybe reflect on the local transfers that you were exposed to? What were your thoughts? obviously very different to what you're used to with specialized teams in the UK?

**Participant 2**

Um, yeah. Well, I think there's a huge variety. So, my general impression of the service and I've obviously handed a lot of babies over to the transport teams here. I think this seems to be quite a variation in confidence, whether that reflects competence or not, I'm not sure but certainly, I get the feeling that there's a very high level of confidence amongst the staff picking up babies for transport. And I think It was the paperwork, the documentation, you know, I didn't really see much of it. But I don't know if that's because there isn't much or because I just didn't see it. But, you know, that's a huge difference to the UK. And my training is that these things in England are highly documented from a sort of medical, legal, but also patient safety perspective. And here, I didn't see a huge amount of formal documentation. And I think the particular babies that I did go with an ambulance where I was probably triggered to do so because I felt that the people who came were perhaps under confident, or the baby was particularly sick. And when I did go, and the paramedic was extremely relieved on both on several times, you know that, mostly because of the handover, at the other side, I realized they weren't that worried about the actual transfer so much, but they seem to be quite nervous about handing over to the ICU doctor, and very pleased that I was there so that I could do that. So again, I feel that that's a documentation issue. Around the people being clear about the numbers and how to communicate effectively to the other side, what's happened, not just in transit, but obviously in the referral center as well.

**Researcher 1**

Thanks, doctor. So just to summarize your experience with local transfers, not just escorting, but people, either accepting patients from you. You say there's a variation in confidence, not necessarily knowing, whether this translates into competence. But you mentioned, one of the shortcomings was documentation. Quite limited when you compare this to the UK amount of paperwork. And obviously, from a medical legal perspective, this is a concern. And then you did escort some of the patients. And this obviously helped a lot, the paramedic was relieved that you could go with because maybe not necessarily patient management, but the handover on the other side to a specialist. Tell me your experience with the level of equipment in the vehicle. What was it like?

**Participant 2**

Good, like I would say quite similar to what I'm used to from back home, actually, the equipment's all very similar to what I been using, you know, this was I suppose I'm talking about the dedicated sprint service so that they obviously have their incubators or set up on the trolleys. And that's what I was referring to that all seems to be good. Like, there was nothing that I thought, Oh, we need this. And it wasn't there, for example, if that makes sense.

**Researcher 1**

So, it sounded like your exposure was with a dedicated transfer team, and that you felt that the equipment levels were adequate. And just some of the individuals may be from a confidence perspective was not that good. Is that correct?

**Participant 2**

Yeah, but genuinely, I mean, I'm amazed by the skills that the paramedics have here and, usually they're very confident and generally, I think I've been really, really impressed with them. Yeah, what people do here. I mean, we've very, as you say, with quite limited sort of backup and support, people seem to take on a huge amount of risk and seem to manage that very well. And so, I mean, genuinely I've been really impressed with the standard of the paramedics that have come to take the babies.

**Researcher 2**

Just as a follow up question, to sort of elaborate on what you've just said, in your view, where do you think these providers get the skill and knowledge in order to be competent and proficient in this and have the confidence to perform these transfers in the manner that you're describing? Where do you think that comes from?

**Participant 2**

I honestly don't know. And I think that's sometimes what perhaps shakes my confidence a little bit rather than this is that I'm, I don't know if this is just learnt from colleagues, you know, that somebody more experienced is teaching someone else or through just sheer experience of numbers of transfers. I mean, they are doing a lot. So, I guess we all learn from our own experience. And perhaps it's, it's just that they are getting more and more familiar with things and familiarity sometimes can breed a little bit of complacency as far as perhaps being overconfident on occasions.

**Researcher 2**

Great, thank you.

**Researcher 1**

Thank you. So just to summarize, the question was: Where do you think providers get the skills from? You said, Not sure. But if you had to guess, from sharing information with colleagues and more transfers comes with more confidence. But then there is a possibility that this familiarity with neonates could lead to complacency. Is that correct?

**Participant 2**

Well, I think, as you've said, like, there's no dedicated formal training, or I mean, I'm not sure what the system is here, do people have sort of regular yearly competency reviews, or as far as their continued employment in a service goes? Because I just don't know enough about the paramedic training systems. So, I just mean, that sometimes in medicine, you know, you learn something from a colleague, but it might not necessarily be quite correct. And then you learn that as absolute and you take that in good faith, and then you continue doing that, and then the next person doesn't, perhaps, you know, things start to drift. So, I think there does need to be a kind of regular review of competencies within the service. I'm not sure if that exists currently.

**Researcher 2**

And I guess this is kind of the risk, of course, where you don't have a particular standard or course that you know, people can perpetuate incorrect information. And that can go into regular normal practice. Really?

**Participant 2**

Yeah, exactly. Yeah, I did also have some paramedics come when I was working in the emergency center. Occasionally, we do have paramedics come with a skills log, to spend some time in the unit with me and do some calculations and are keen to know if there's an airway. They're keen to get involved. So, I do know that there is obviously some system where people are getting sign offs, but I don't think that's specific to neonatal skills. That's more pediatrics.

**Researcher 1**

Okay, thank you, doctor. So, my question to you next would be, how much do you know about South African paramedics? Do you know what advanced life support providers are? And our history in education and the changes over the last, let's say, decade? How familiar are you with the qualifications?

**Participant 2**

I'm not very familiar with, so, please educate me.

**Researcher 1**

Historically paramedics, as we call them, had a short course approach to training. So, it would range from a one month, two or three months to a nine-month course, there was also a three-year full-time course, which was a national diploma level. And then currently, this is changed so that they are now a one year two year and a four-year qualification. And I guess that it doesn't matter your qualification, everybody is still called a paramedic. So, when we refer to advanced life support paramedics, we usually talk about people that's done a two year and up qualification. And these are usually the individuals that you would see on an ambulance that would be doing these transfers. But the background in education, the institutions, they come from the methodology, and the timeframes vary a lot.

**Researcher 2**

Yeah, I think that what it's really good about this particular interview is the fact that we will not find another doctor to interview that has experienced from both sides and has extensive experience in moving units. And I think what's important here is that we should take the interview in a direction where we're falling straight into the curriculum elements. Because we need to learn from international colleagues, and you know, so that we can actually also align it to international standards, but also because your discussion schedule and the documentation that you sent, is actually based on mostly international literature, of course. So, it's quite important that I think we just move into talking about the curriculum itself. And then we can talk about other system issues as they come up, as they relate to the curriculum. I think, if that sounds like a reasonable plan?

**Researcher 1**

That sounds perfect. Thank you. So, doctor, in the background reading section, I did do a literature review on neonatal specific training for transfers. And there was only really one local study that compared the different curricula for the universities. And then I found international courses as well. And one of them, as you said, the book that you were familiar with. And is there any other type of training that you know of that people do internationally to cover these gaps with neonatal specific transfers.

**Participant 2**

So, whereas as I said, it's, it's very different in the UK, because we are an ICU outreach, essentially, we're an extension of the ICU that traveled out to pick up babies and come back to our unit. So, the training actually is within working within the neonatal intensive care units. So, for example, I would have a logbook, and when I was training, and I would log all the transfers, I went on a lot of transfers before I let them so I would go as an extra person and as a junior doctor, and I would have a logbook and I would be having to document and debrief all those transfers with senior colleagues in the unit. So that's the learning and of course a logbook of skills as well. And then once I had sort of satisfied all the competencies as far as the number of transfers and the skills logbook, and that was supervised, and I then moved on to start doing leading my own transport team, and that would be initially on fairly low acuity transfers, short distances. And then, you know, in my senior years of training that see progressed to being munch more sick babies, and so it's very much a continual progression of skills and development throughout training, it's not a one-off course. That makes sense?

**Researcher 1**

Thank you, that makes perfect sense. So, you had to show a portfolio of evidence logbook? And then you will not be the leading physician initially. And then this would then build up from low acuity patients when you started leading to higher acuity patients? Is that correct?

**Participant 2**

Yeah, that's correct.

**Researcher 2**

Just maybe a follow up question. If I can get your opinion. If we have to transplant that system into this African context, or in the low middle-income country context? What alternatives Can you maybe think of, in an instance, where we might not always have the luxury of having, you know, doubling up the clinician on a transfer? Because there just aren't enough people to specifically do that? And what kind of alternative educational approaches Do you think might be able to almost replicate that kind of a model, but without being so resource heavy, if you had to just spit ball?

**Participant 2**

Yeah, um, I mean, I was thinking about this. And I was thinking, you know, how do you enable people to access this kind of training in such a resource limited environment, and particularly in a place like South Africa, which geographically is huge, and you've got people in a working transporting the units across the country. Thus far distances from the district generals or the clinics to some of the tertiary neonatal units in some cases. And I suppose people therefore, physically live in places far away from the neonatal intensive care units as well, because I mean, I was thinking, I was wondering about time spent on a neonatal intensive care unit, whether that would be useful for paramedics undertaking these transfers, to have spent some time working alongside neonatal consultants, or physicians in the dedicated tertiary units. But I suppose that's also tricky, because there aren't that many neonatal intensive care units, and they're only really in the major cities. So, I guess it's difficult for people who are working more remotely to access that. But maybe it could be a sort of intensive block of a couple of months, rather than a sort of longer period of time. Maybe that would allow the paramedics to feel to gain the skills as far as some of the practical sort of emergency skills might be required. I mean, I'm thinking particularly around managing airways and ventilation, some of the sort of troubleshooting on the vent, and emergency access techniques, I guess, those are things that really could be learned, while spending time in a neonatal unit. I mean, even if I think about the neonatal unit, I mean, it's only a district General Hospital. It's got a small neonatal unit. We don't ventilate babies there, but we do have sick babies born there plenty and those babies need to be stabilized and often are ventilated and often have central lines and have you prior to transfer, so even time spent at a district General, to be involved in that process might be useful as far as training is concerned. In the same way, by the way, I think doctors should all really be spending time with paramedics. Learning about how their job works. In order to understand better, what happens when you put babies in the back of these ambulances, as you say, to gain a better appreciation of each other's skill set, it's really important to be able to work together in a team. That will be my thinking.

**Researcher 2**

Absolutely. Thank you. Yes, I think you can continue from there.

**Researcher 1**

Okay, thank you for that question. So, just to quickly summarize this section. The question with our limited income country like South Africa with limited resources, how would we employ a mentorship program like this? And your suggestion was to include an in-hospital training way, and the paramedics would spend some time in NICU, or in neonatal units or casualty with doctors and nurses and use the training and confidence in that way? Is that correct?

**Participant 2**

Yeah, yeah.

**Researcher 1**

All right. So, with your experience in neonatal transfers. And you've seen a bit paramedic and how they present in South Africa. If we had to sort of outline a broad knowledge base and skill sets for these paramedics, if we wanted to do additional training for them. Can you maybe give us an outline of what you think should be contained within that knowledge base?

**Participant 2**

Okay, I had to think about this. I think firstly, an understanding of the scope of the service that you're delivering and by that, I mean that you're not an ICU outreach. And I think people in hospital teams and transport teams need to have very clear understanding about who, you know, are the patients to be transferred, and where they go. So, the networks and directions of escalation. So, I think you need to know the scope of what you can deliver, where the limitations are, and the physical sort of directions of care within the networks. And importantly, I think there needs to be some objective, like, parameters of what is stable, I mean, what you could ask lots of people what is a stable neonate, when is a neonate stable for transfer, and I'm sure many people would give many different answers. But I think there needs to be an agreed criteria, really, for your own safety as far as your own risk is concerned. And so, I would think that there needs to be an idea of that, and a little bit of the ethics around when not to go. And so, some of these babies, you know, you might be asked to pick up are in a state where actually they are unlikely to survive the transfer, or, you know, even if they were transferred, the outlook is that they are not going to survive, that there's no chance that they'll survive when they're at the unit you're taking. And I think it is your responsibility, as you know, you're taking over the responsibility of this child when you pick them up. And I think you do have to have a consideration of that. And I think if you felt uncertain that either the baby was not stable enough to be moved, or that actually it was inappropriate for the baby to be moved, I think that the paramedics ought to be empowered to raise that and have a person to call, I don't know who that would be the consultant at the risk referring unit, perhaps directly to have that discussion. So, one of my feelings around that education would be really around the sort of decision to transfer because you may think that it's the decision of the person that's asked you to come pick up the baby. But actually, it's your decision when you pick them up whether you're taking them or not. So, I think that needs to be a part of the education. And then along with that communication, so it's hugely important, the documentation and the handover communication are hugely important. And we are only going to find that we are living in an increasingly challenging world as far as our risk and medical legal risk is concerned. So, I would suggest that we have a fair amount on documentation and written and verbal communication at handovers and then really, I think you move on to more of the things that you'd be expecting. So, management of the baby, I saw in your background reading you, highlighted the diagnoses of the babies that you'd been transporting in South Africa, and particularly around congenital heart disease seem to feature quite heavily. And personally, I feel that really, you need to be looking at learning to manage physiology, rather than learning to manage specific diagnoses. So much like I'm sure you already do in the paramedic world, and certainly in the intensive care world, we would manage the ventilation, the perfusion, the sedation, and the thermal regulation. And I think those four things, you know, are absolutely key, and then you have your associated drugs with each of those subcategories. So, I personally wouldn't look at how to manage a baby with pneumonia, or how to manage a baby with a PDA or some other horrible coarctation or something, I would really look at it as Okay, how do I find airway comes first, but then how do I manage this baby's ventilation and oxygenation? And then how do I manage this baby's perfusion, and the sedation. And then, of course, thermal regulation is so important. And that's a big area that I don't see. Practice, perhaps because it's so warm here. Most of the time, we don't think about it, but it can be, you know, winter months are cold. And you know that temperature control is a huge thing on transport and your admission temperature for a neonate at the receiving hospital. If there are hypothermic, that admission temperature is more predictive of their mortality than their gestational birth weight. So, it's I mean, it's hugely important and overlooked. So, I think those four things have to be key in sort of managing physiology. And then specifically, you know, in transport, there are some physiological considerations. I don't think it's as hugely, important as is written about all the time. But certainly, we need to be aware that there are hemodynamic important consequences of an acceleration, deceleration during transport and movement and sound considerations for neonates. So, the general physiological categories and then physiology and transport specifically, and then I would have about the equipment and the safety environment in the ambulance. So, learning about how all the equipment works, I mean, paramedics are brilliant at the equipment. I'm often asking them particularly you know, well, the lines and tubes and everything plugs into the machines, but you know, when things start alarming and something says on the ventilator, you know, hi minute volume and it starts beeping at you, just troubleshooting around the equipment. I think you really need to know your equipment well fought for your transport. And then lastly, I think the practical skills really is just for absolute emergencies. You really, practically it's just going to be the baby should be packaged by your referring team with your help. And your practical skills. You know, if you're to be secure and your lines are secure, etc., then hopefully shouldn't be required. But I guess we always have to prepare for disaster and so emergency practice skills such as bag valve mask, ventilation, and placement of an ET tube and then IV or i o access, and needle decompression of a haemothorax probably be the limit of practical skills, I would have thought necessary. I'm not even sure if those are entirely necessary. I think. Yeah, that would be my thoughts on roughly. But my quick thoughts that I'd love to give it more in depth thought. But those were just my quick thoughts, having read the material that you sent me and having a little think about it this morning.

**Researcher 1**

Okay, perfect. Thank you. So that's a big section for me to summarize, but let me quickly run through it. So, when we were talking about curriculum for extra education in this field, you started off with saying, the scope of the service. And so, they need to realize that not all these ambulances are not an ICU, and then also which patients are transferable and where they are going. So, we need to understand the network of where these patients are going. Then also safety parameters, we need to define what is a stable neonate, and there needs to be an agreed criteria, and then the ethics. So, when not to transfer a patient, especially if the outlook is bad, and the patient looks like the patient won't survive transfer, the paramedics need to have the voice to say no or to consult with maybe the receiving specialist and to discuss, to not transfer if it's not in the patient's best interest. Then you mentioned the section on communication. Documentation is very important from a medical legal perspective, so the written and the verbal handovers is very important, then management, you did mention and you did refer to the patient data that I sent you in the prereading document. So, you mentioned that congenital heart defects were one of the highest rates. And if you were going to suggest how paramedics should be taught on how to manage these patients, it shouldn't be specific to the disease, it should be from a physiology perspective and not so much the diagnosis. So, you mentioned four areas that is critical to manage these patients. You said ventilation, sedation, thermoregulation, and perfusion. You said that, from a thermal regulation perspective, the admission of neonates to ICU is a big predictor of mortality rates if the baby is hypothermic. And you mentioned also the physiological considerations during transfer. So, your acceleration deceleration affects your noise, your vibration. So, this needs to be covered in that kind of education, then your equipment safety, you really need to know how your equipment works, if there is alarming of any equipment, how to troubleshoot it, and how to address the problems. And then lastly, from the practical perspective, you need to prepare yourself for any type of emergency. And so, you suggest that the referring team helping package the patient to ensure that the tube is secure, the lines are patent. And then from the paramedic side, they need to prepare for disaster. So, they need to know how to intubate and bvm the patient if something goes wrong, and then worst-case scenario, you mentioned needle decompression, and also how to ventilate the baby. So just for your interest. Needle decompression is on the scope of an emergency care practitioner for neonates. But it's only one type of qualification that's can do this. Did I capture that section? Is anything you want to add or change?

**Participant 2**

No, I think you're far more articulate than I am. Thank you for summarizing that so nicely.

**Researcher 1**

Alright, so if we look at this type of additional training, if you had to say how long do you think this type of training should be?

**Participant 2**

Like that depends if you're going to do this full time or part time. So, if this is going to be something that you do as an intensive course or if this is going to be something that you do alongside your working week.

**Researcher 1**

Alright, so you say it depends on whether it's a part time commitment or a full-time commitment. Let's say it was a full-time program, how long would you suggest this type of training to cover this specialized field would be?

**Participant 2**

Oh, goodness. Really? I'm really not sure. It's a lot. And I think, the reason that I struggle with this answer is that I really don't have a clear enough understanding of the base knowledge of the people that will be entering this program. So, I don't know, you know, how much of the baseline is there? So, it's difficult to answer how long it would take to get to the standard that I'm imagining not knowing really, where your basis, sorry, I'm not familiar enough with the with the, with the skill set that you would be entering onto this from me.

**Researcher 2**

Maybe if we had to rephrase it slightly, but I can also give you a little bit of a hint on who it looks like it might be it is, it would probably be the same kind of people that would be entering into the Post graduate diploma program. And so that would kind of be where they would start from. But I think outside of how long, maybe we can rather put it in a way of saying, do you think that this should be a short course? Do you think that there should be a diploma course an actual master's degree? Maybe somewhere around that, especially if you're considering maybe going into being a dedicated neonatal retrieval person? Does that help?

**Participant 2**

Yeah, goodness, it's complete. I think that this has to it. So, if you're talking about a course, this has to run with what we were talking about earlier, have some face to face time in units. So, I don't think this is something that can't be delivered, like an entirely online course, or diploma? I think this has to, you know, the content of other course, would have to run with some time spent in units. And I don't think that necessarily has to be dedicated intensive care units, if that's not sort of geographically feasible, but certainly units where babies are being born and resuscitated and stabilized and transported from. So, I think it would have to include some of that time. And if you were going to make a short course. Yeah, I think it could be a short, I think this could be a short course. I mean, as we've said, we're not. We're not, this is not training to be an ICU outreach team, it's a different scope entirely. So, I would think that you could do maybe a six months module, your short course, including a couple of months in a unit or some sort of specific contact time within that built into that. Right, that's, that's really thinking of how to get the service to a safe transport service and not an ICU outreach service.

**Researcher 1**

You mentioned that it should be face to face. Contact with neonatal ICU or some in hospital experience is essential during this training. So, if it had to be a short course, and if we had to assume that there would be a good base knowledge for these paramedics, six months with additional contact sessions would be what you would be suggesting. Is that correct?

**Participant 2**

I think so. I'd Honestly, I think so I think I'd have to think a little bit more about it really, as far as the exactly how it would work timeframes. But yeah, I think if you had it six months dedicated training to neonatal retrieval, I think we could achieve a lot. And yeah, I think that would be, that would be a good place to start.

**Researcher 1**

Okay, perfect. Thank you. Can I get your opinion on? How would we assess these type of curricula that we discussed earlier? So, in your opinion, what methods of assessment is quite effective to evaluate these paramedics?

**Participant 2**

Well, it's a question of resources. Because I would say that the best assessment is actual observation and discussion with somebody who has more experience in the world of neonatal transport. So, you know, the best way is, I think, a skills log and a transport log of experience, and then discussions with, you know, debriefs, and discussions around that. I think, specifically looking at learning about managing the physiology and learning about your equipment, and even your communication and the scope. Those things can be can be delivered online, I mean, that and can be assessed via a mixture of discussions. And so, discussions with a tutor around a topic, like a viva , I don't know if that's a very old-fashioned word to use, but we used to have like OSCE VIVAS. So, I would have thought you could do some sort of simulated scenarios. And yeah, sorry, I'm thinking out of my head and it's all coming out jumbled. So, three things one is mental appraisal on your practical on your contact, attachment time, one thing would be simulation scenario and scale testing, and then you could use a mixture of the simulations, you could also have some OSCE Viva discussions, I mean, you can use some essay based and then MCQ based type assessment tools, sure, for some of it, but a lot of this is far more practical and more difficult to assess by those traditional methods, but I think you could probably 50/50 as far as assessment will be concerned. So, some face to face and some and MCQ type short answer questions.

**Researcher 1**

Okay, perfect. Thank you, let me quickly summarize your opinion on assessments. So, you said mentorship or observation is very important. So, this is where a senior or specialist person would have a period of observation. And then there are discussions or I'm following this observation and that's one method of assessment and also learning. Then you mentioned again, portfolio of evidence is very important. So, you would have a logbook, where you log your skills to show that you've had exposure to these type of skills. And then when it comes to equipment and physiology and various other fields usages that online discussion types of assessment could be suitable. You mentioned VIVAS and OSCEs as also a type of assessment for these areas, and also simulated scenarios. And then also, you mentioned the short answer-based tools. And then short answer questions, and also some practical assessments. Did I capture everything?

**Participant 2**

Yes, I think I mentioned every possible tool of assessment. Sorry.

**Researcher 1**

And so, if there was something that last message that you had to give the curriculum development committee, as what is the most important things to consider if additional course or training was said to be developed for the industry?

**Participant 2**

I think I would say, know when and who to call for help. I think that's really important. I think they have to have backup, and they need to recognize, you know, being able to recognize when they need assistance, because there is always assistance available. That you have to ask for it.

**Researcher 1**

Okay, thank you. So, you say it is very important to include in this type of education that especially within a low to middle income country, like South Africa, and our limitations is that you need to know who to call and when. And you need to recognize that there are limitations, but that there is help and that there is someone that you can call. Is that correct?

**Participant 2**

Yeah.

**Researcher 1**

Okay, perfect. Thank you.

**Researcher 2**

I think also, just one of the things that I think is coming through quite strongly in your answers is the idea or notion of interdisciplinary collaboration between the different role-players is almost that it's not something that should occur in isolation with any one profession. And that there should be, you know, cross disciplinary learning, and communication throughout. Not just the development process of a curriculum, but also really through the, the actual conduct of doing these transfers. And regular communication between all of the different role players from is a systems perspective. And I think that's if I understand correctly, that that's what you mean, it's I think that's a very important element for us to consider in the design of, this curriculum and courses like this, is to always keep that in mind that it is not one group in isolation. And I think that's almost something that paramedics often feel is that it's just them in the back of the ambulance. And I think that you're making a very, very good point. Related to that.

**Participant 2**

I thank you for summarizing that. I think that's exactly how I feel. Yeah, absolutely. This has to be interdisciplinary. And I'm amazed at the sort of disjointed situation at the moment where, you know, there really isn't enough support across disciplines like this should be a team effort. I absolutely agree. This needs to be looked up from that perspective.

**Researcher 1**

Great. Thank you. So, if there's nothing else you want to add, I'm going to stop the recording before we say goodbye.
