# Supplementary material for: Developing a South African curriculum for education in neonatal critical care retrieval: An initial exploration
Source: PLoS One. 2023 Aug 31;18(8):e0290972. doi: 10.1371/journal.pone.0290972 (PMC10470938; doi:10.1371/journal.pone.0290972)
Supplement: S1 Data — (ZIP) [file pone.0290972.s002.zip › Data Compressed/Transcript 3.docx]

##### Interview 3

##### Researcher 1

Good morning, thank you for joining us. So just to let you know that we are recording this session. Just to confirm that you are giving consent on the recording.

##### Participant 3

Yes, I am happy

**Researcher 1**

Thank you, just to confirm that you received the consent document?

**Participant 3**

Yes I did receive it and I sent it back to you this morning.

**Researcher 1**

Just to summarize that, just want to confirm that this interview is voluntary and you can withdraw at any time. Any personal information will be anonymised during the transcription. Are you happy with that?

**Participant 3**

Yes, sure.

**Researcher 1**

Just a quick background to the study:

- the transfer of neonates in South Africa is performed by advanced life support (ALS) providers.
- This high risk service is performed by specialist teams internationally.
- Adverse events during these transfers have been associated with the providers’ level of knowledge.
- South African ALS providers have variable education backgrounds.
- There is no guidance from SA governing bodies on the method and content of education in this specialised field.

Can you please start by telling is about your background , your education and your experience in neonatal critical care retrieval?

**Participant 3**

From an education perspective I started after high school in a nursing degree at xxx University. I worked full time in the emergency department for a year, before going over to study emergency medical care. Completed that, and started working in road ops for about a year. Then moved over to clinical care retrieval. Critical care retrieval career started in 2013 and that is basically what I have been doing since. I have always had a special interest in neonates and encountered a lot of neonates on the critical care retrieval service. So I identified a big knowledge gap. I spent a lot of time in hospital in neonatal ICU. From a non-formal education perspective I spent quite a bit of time in the neonatal ICU at the hospital that I was based at. That is obviously non formal training. Then I did some critical care retrieval courses here and there. That is essentially the training. In theory I am enrolled for a master’s degree but I don’t feel like putting that on the table.

**Researcher 1**

So you have got an in hospital background with the neonates and then you also did the paramedic degree, and then you already mentioned that you identified gaps. Can you elaborate on when you identified the gaps. Was it when you started in 2013 with the transfers that you felt there was a gap or when was this?

**Participant 3**

It was pretty much in 2012. I think I was qualified for a month or not even and I did my first ventilated neonate transfer. At that point I was working and they did not have a dedicated ICU unit. I was an ops paramedic when I did that and I felt completely out of my depth. Taking the baby from the environment that it was in and putting it in the ambulance, I felt like what am I doing here. I was the only option to do it. Encountering ventilated neonates I know I was completely out of my depth.

**Researcher 1**

You said you closed the gaps and it was self-initiated and you did in hospital training and also critical care retrieval courses. Is that correct?

**Participant 3**

Yes

**Researcher 1**

Just reflecting on the bachelors degree that you did for pre-hospital. Do you think that curriculum was adequate to prepare you for neonatal transfers?

**Participant 3**

I definitely don’t think so. You spend quite a bit of time in the neonatal units. The problem with that is that you are a bit of a side-line person. There are registrars as well and I can completely understand why we don’t take preference in that scenario. Its not anyone’s fault. On paper it looks like we spend a lot of clinical time in a neonatal unit. In reality what you actually learn there is very little. You see side-line things. You do not learn how to adequately take charge of critically ill neonates.

**Researcher 1**

You have been involved in the program that is currently running. So from when you did your qualification to when you got involved in teaching on the current program, did it change? Would you make any changes to the current curriculum as it is with regards to neonatal critical care transfers?

**Participant 3**

What we basically added was some lecture content. So no, I wouldn’t change the current curriculum. This is a personal opinion completely. I do not believe newly qualified paramedics should be caring for ventilated neonates. I feel neonatal retrieval should be a speciality on its own and should be done by speciality teams. I think what it requires to safely care for a sick neonate, you are not going to teach in a couple of lectures for a University student. Ideally yes if we can edit the time spent on neonatal content we could get more value out of it. I don’t think they can be adequately prepared in the timeframe.

**Researcher 1**

So to summarize. There is limited time on the bachelors program and that you would like to see more time spent on neonates. You feel that the guys that qualify from the bachelors program should not be caring for ventilated neonates and should be reserved for specialist teams. Is that correct?

**Participant 3**

Yes, essentially it doesn’t have to be a neonatal critical care retrieval course, it can be a critical care retrieval qualification for the newly qualified guys.

**Researcher 1**

So you are touching on my next question then which is you feel that additional training is required for people that do critical care retrieval. If you could put a time frame on it and the type of training would it be a post graduate diploma or masters level or what type of training do you think this should be?

**Participant 3**

I think its both. Similar to the model that the hospitals use with nursing, where you get the six month in service training. It’s an official six month training program with a lot of practical work or you can do the masters in one of the nursing divisions. I feel like it would be very similar. There is enough content to learn to justify a full master’s degree. In reality you need more worker bees if I can call it that. Appropriately skilled and equipped individuals, not just masters candidates. I feel like there should be at least a six month program very practically orientated with all the theory as well. You can’t just do a theoretical thing is the bottom line for me. There needs to be theory content but a lot of that can be done online as part of adult learning. The guys need to work on a critical care retrieval service for hands on experience. You also need to work in the neonatal unit hands on. Not like the current fourth year students that are standing in the corner looking at what the other people are doing. So a mixed approach. So you would start with a six month program and work your way up. Maybe a PG dip program is a better idea. The one year program and ultimately a masters.

**Researcher 1**

If it was a post graduate diploma in neonatal critical care retrieval, do you think it should follow directly after the bachelors program, or should it be a requirement before you can transfer a neonate?

**Participant 3**

I don’t have a problem with it following directly after your bachelors because it wouldn’t be a full time thing. You would be working full time and building up experience anyway, but at the same time you would be building up experience in a specific field. So I suppose it would depend on how it is designed. I think what matters is that you do have the additional qualification before you perform neonatal transfers as primary crew. It should be a minimum requirement.

**Researcher 1**

With our setting of a low and middle income country. Specialised retrieval teams are a scarce resource. Your emphasis on this training was on practical exposure. How could we get around this with limited teams for the guys to work with.

**Participant 3**

I do think that there are enough people that would be interested in getting the additional qualification, to say that over and above my regular hours I am willing to do an additional 5 shifts a month. I do think it is going to rely on people putting in additional effort because of the resource limited setting. In a first world country the employer will pay for you to become part of the critical care retrieval team. It will have to be people that say they are willing to put in the additional effort. I would like to think that there will be people willing to that. There are not a lot of retrieval services around at the moment, I fully hear that. I think it will have to be a staged roll out. Let’s say we start with in hospital training more, then once these guys reach their six months, the first couple of years of something new is always going to be less than ideal. Focus on in-hospital training first and then build up the neonatal critical care teams. They would already be a 100% better than the guys that come straight out of university.

**Researcher 1**

So just to make sure I understood you correctly. So you are saying that yes, we do need the additional training. You suggest an additional 6 months like the nurses do for hospital probably up to post graduate diploma. With our setting, working with specialised teams might not be that practical so we might want to look at in hospital exposure to neonates. Is that correct?

**Participant 3**

Yes, that is technically the path that I followed. The majority of my neonatal exposure has been in hospital because it was available.

**Researcher 1**

And then you were saying that one of the barriers could possibly be that the employers may not be willing to pay for this additional training and that it would have to be self-initiated from these guys.

**Participant 3**

I do think that that may be a challenge. When you qualify and get a full time job, the way it is at the moment is, employers will say that I am not paying extra if things are working the way it is.

**Researcher 1**

With your experience in neonatal critical care transfers if we had to sort of group knowledge into specific sections of what you think these guys will require. If you can give it some broad categories of what should be included.

**Participant 3**

I think the basics would be a big part of it. I would say basic neonatal assessment and monitoring. What is normal for a neonate? So many people don’t know what is the normal blood pressure for a neonate. What’s your target? If you don’t know what your target is you don’t know what you are doing. So I would say assessment, neonatal monitoring.

Then I would say specific neonatal conditions. The conditions I would split into broad categories. Surgical emergencies like your open abdominal things and NEC. Then your congenital abnormalities which involve your airway, your head, your neck. Cardiac would be a big thing. Then pre-maturity I would put as its own medical emergency or disease category. Then under those I would further expand. I think that is what I will do. Say, assessment, monitoring and then neonatal specific conditions and split them into categories.

**Researcher 1**

So you are saying then that the basics are quite important, so we need to know what is normal. What are the normal parameters, and then you would break up the content into the different conditions. Whether it be congenital, prematurity and the considerations that go with each. So the hospital setting is quite different to the ICU (ambulance) setting. Would you include anything from that perspective consideration wise, training wise?

**Participant 3**

I would, because that will fall under generalised or continued care. Things like feeding, what feeding are they doing in hospital or why, how. It does impact your transport journey to have specific considerations for what continued care is going to be once the baby reaches the hospital or for that break period in between. Hospital to hospital is different in a closed incubator vs this is a open bed cot in hospital and temperature requirements with that. We need to make a comparison between in-hospital care and how you are going to manage it during transport.

**Researcher 1**

I hear you that things like feeding that happen in an ICU setting, it needs to be continued in the ambulance but the environment has changed. So these things need to continue but how do we do it in the ambulance?

**Participant 3**

Yes, and when do you continue. Sometimes it is important to continue the feeding and sometimes you wouldn’t. Stuff like that, when do we continue, how do we continue, how do we marry the two fields of in hospital and out of hospital.

**Researcher 1**

Then, with your position, you have had oversight of many transfers and various practitioners. Can you highlight the areas that are often a shortfall that will require training?

**Participant 3**

I think a massive shortfall is basics. Honestly just what do you do with a baby, how do you position a baby, when do you know that a baby is in distress? When do you know that a baby is decompensating? So I would definitely say basic assessment and care of a neonate is a massive gap that then translates into the bigger things. They are already comfortable with ventilation, it is just easy to adjust. Ok maybe I will just adjust to a lower pressure or rate. That is not the biggest learning curve, the biggest learning curve is that it is not just a tiny adult. That’s where the biggest gap would come in and then obviously the neonatal specific. You don’t see an adult with transposition. You don’t see an adult with a perforated NEC. There is a difference between an adult with abdominal sepsis to a neonate with perforated NEC. Basics then neonatal specific emergencies.

**Researcher 2**

I just want to get a little bit of clarity on, it sounds almost as if you are saying that there seems to be a deficit in basic undergraduate training for general neonatal care. And this kind of sounds like what you are talking about with regards to the basics of care. Because that is theoretically something that a technician should be able to do. Anyone that has neonatal transfer on their scope of practice. What I am hearing is that you are saying that there is a deficit there from the outset. Just go back a little bit to what you said with regards to undergraduate training. At the same time it sounds like you are saying that while there is this deficit with basic care there is also on top of that, the care that a retrieval person would need to learn about for neonatal care. Is that kind of right?

**Participant 3**

Yes, I definitely think that if we could improve the basics in undergraduate training. The problem is that you can only learn about what you can see. You can tell me how many times in a presentation, this is what a neonate looks like with poor perfusion until they really see that neonate. I think that is maybe why the basics is so lacking because they don’t see neonates. Even that time in hospital xxx as a student, you see them but you see them from a distance. Yes, definitely a gap in undergraduate training that carries through to the specialised knowledge/care.

**Researcher 1**

The equipment that we use in hospital and in the ambulance. Do you think that needs to be included? The differences?

**Participant 3**

I am going to say yes, and people may say I am a snob. Because I have the nice things (equipment). We have nice things because they do impact the care of the neonate. So I do think that the equipment needs to be included and there should be a minimum standard. Everyone that does neonates need humidification for example, because it changes their prognosis and outcomes. Also knowledge of the equipment. It doesn’t help you running around with a neopuff and you don’t know how to set it up safely.

**Researcher 1**

Did you review the neonatal data from xxx study?

**Participant 3**

Yes

**Researcher 1**

Do you agree with the disease profile and the attachments? Is that kind of what your unit is seeing? Is it different?

**Participant 3**

We definitely see more premature neonates. Here is quite a heavy distribution of cardiac neonates. We see a higher percentage of prematurity as well. We do see a lot of CHD as well. Prematurity and CHD are the bigger ones. Things that we tend to see a little bit more of like under CHD, we see Sildenafil and Viagra which we are seeing a lot. We also see a lot of babies on nitric oxide. You then need to take into consideration taking them off the nitric. There were some small things I would add. On a broad/overall level I don’t think it is a bad representation. 25:55

**Researcher 1**

So that information that you looked at was a national sample. So what I hear you say is that for your unit, prematurity is up there with CHD and some medications more so than what is on the list. So when we take that information that was from xxx study, and the cases that you are seeing. How do we bring that back to training? What special training is required based on those patient profiles?

**Participant 3**

I think that the stats fit in pretty much smack bang with how I envisaged us covering the topics is that it shows you the main criteria of patients that we see. I think if we include in the curriculum a sub set of these conditions that fit in very closely under those categories that I mentioned. I think it speaks nicely to each other. Those are the babies that we are seeing is the bottom line.

**Researcher 1**

So what you are saying is that when the curriculum design is performed those type of topics with the disease profiles should be included?

**Participant 3**

Yes, it should take data like this into account.

**Researcher 2**

This sample was in private care. Do you have any sense of what this data might look like in state care or not?

**Participant 3**

My opinion will be based on two individuals that I have spoken to that have or are currently working in the government ICU system. From what I understand from them is that they do tend to see less of the very complex neonates that we tend to see. Because they tend to pick up neonates from more basic facilities with very initial interventions. Then they upgrade them to specialised care. I don’t think they will ever see a neonate that is currently on nitric, or a baby that is post a catheter procedure with an open chest. So I think in public you might see a little more complexity and see more primary sick neonates in government. So, like a baby that has not been tubed yet. We would find the baby tubed, or already on CPAP. Where in government they might find a baby gasping for air in a warmer.

**Researcher 2**

So what you are saying is that for the state sector it is more acute resuscitation, whereas for the private sector it is a continuation of more complex care?

**Participant 3**

It is more ICU level of care vs government with basic resuscitative care.

**Researcher 1**

So we don’t have those statistics but lets say that in the state sector it is 80% of neonates that are being moved and that it is more basic interventions. So if there was a study that combined state and private statistics. I think it will change the scope of what should be covered in training don’t you think?

**Participant 3**

I do think that it possibly could as there will be greater emphasis on resuscitation. I do think that it is very important to look at both. The aim of a training program would never be to train someone for one or the other. We would need to put someone out that can work in either and do well in either. I do think that it would be very important to include the government data.

**Researcher 1**

Yes, we definitely need to consider that and that it will affect the type of curriculum that is put together. So in that document there is also a literature review. There has only been one local study that compared the local university curricula. Did you review that and what was your opinion on the content?

**Participant 3**

I didn’t really look at it in depth.

**Researcher 1**

There was only one study then and it compared the curricula and I think it is quite in line with what you said earlier. Like the time frames and what is included. There were these international courses then as well that cover neonatal critical care retrieval. I don’t know if you looked at the sources that were found? Is there anything else that you would add or do you agree that what was found is the only things that are available?

**Participant 3**

I am very hesitant around the international stuff that is available just due to the fact that our environment is so different. Some of the things that I have looked at before with regards to neonatal retrieval. Fore someone who has very little neonatal knowledge those can still add a lot of value. Some of the ones that I was interested in I couldn’t actually do because I am not a physician. So the level of knowledge that our people will require is not accessible. The international paramedic qualifications would not take on those roles.

**Researcher 1**

We come to the conclusion then that with our local setting and the patient stats that we have that the international courses might not be a perfect fit. The equipment and the setting is different. You mentioned that access to some of these courses is a barrier s it is reserved for physicians and not so much for paramedics.

**Participant 3**

And the cost associated with them as well.

**Researcher 1**

If a new course in neonatal critical care is developed, how do you think this should be implemented? From a national perspective? Should it only be a small local short course or should it be the national standard? How do you think this should be rolled out?

**Participant 3**

It definitely needs to be national. We will never set the standard if we do things on a small scale. It needs to be a national standard that neonates do not get moved by non-qualified retrieval teams. If you are doing your own little course, it is great for the patients that our people will see, and you might improve the outcome of 50 neonates but the outcome of 500 others are still going to remain unchanged. So definitely national level. It requires funder buy in as well.

**Researcher 1**

So one of the barriers would be funder buy in. Any other barriers that you would perceive between different parts of the country, institutions and governing bodies?

**Participant 3**

We have to start on a small scale if there are only that many of us available who have experience and knowledge in neonatal critical care retrieval. You can’t launch a training platform where you are going to need ten instructors or lecturers when there is only 3 or 4. Implementation will be a challenge, for example people are going to have to travel to attend practical classes. The practical application will be a challenge on a national level. You might have to start small but the aim will be national.

**Researcher 1**

You have an understanding of when we say ALS providers and their educational background. The different types of training historically and different NQF levels. How do you see this type of additional training fit in with the different qualification levels?

**Participant 3**

I do feel something like this should be open to all. It is complicated as the ANT register includes ECTs and I don’t think they the new diploma graduates are ready for this. Someone who has received ALS education like CCAs and N.Dips and they work in the correct environment I don’t see any reason for them to be excluded from something like this. At the moment the varsity level is so lacking when it comes to neonates that it might not make a difference if you took a CCA or an ECP. Their neonatal knowledge is equally lacking. I would say that all CCAs, N.Dips and ECP should be allowed access to train in a course like that.

**Researcher 1**

So you are saying that it should be inclusive for all advanced life support providers when we talk about the older qualifications.

**Researcher 2**

I just want to get a little bit more of your opinion here. You are saying that it should be for the traditional ALS cadre. I want to ask what do you think about how that would translate into scope of practice? You are highlighting the complexity. So during this course what does that mean for the neonatal elements associated with it?

**Participant 3**

If you look at nursing, when you have done the four year degree at university and you do additional courses it allows you to do different things when compared to someone that did the in hospital training which is just a resisted nurse and not a professional nurse. An enrolled nurse can also enrol in these courses. Their enrolment doesn’t now mean that they can administer advanced medication that is reserved for registered nurse scope. So if we could run it like that, it means that we say yes, the CCAs can come and do it as well. The diploma guys can do the six months as well if we can pull it off. It doesn’t increase your scope and in reality you will not be the primary crew on a retrieval team. You will form part of it. But it still allows you to take part. I don’t think it should be a big issue.

**Researcher 2**

So what you are almost saying is that the participation does not go hand in hand with a change in scope.

**Participant 3**

It makes you a better practitioner in your patient care and that should be enough motivation to do the course.

**Researcher 1**

What is your opinion on the type of ALS providers that are moving neonates within the country qualification wise? Is it ECPs, N.Dips or CCAs or new diplomates? I know that you don’t know each services numbers but if you had to give us an idea?

**Participant 3**

I do think there is a difference between rural and urban. Where in Johannesburg it is ECPs and in Welkom it is a CCA. And it Kathu it is a CCA. That is why I think the reality is we cannot exclude those people. There is still a number of neonatal retrievals being done by the OLD ALSs.

**Researcher 1**

Do you think a single course will cover all of the neonates’ needs if the biggest volume of patients being moved have basic needs? You mentioned the state sector require more acute resuscitative needs and private more high acuity level of care. Do you think one course should cover everything or should it be split?

**Participant 3**

If you include enough and the correct practical environment. I do believe there should be two pathways. There should be the 6 moth section for the non-primary crew of retrieval teams and then potentially PG Dip and Masters. Because there is a lot to learn and you cannot learn everything in 6 moths. You can affect the outcome of a lot of neonates with the 6 month program. For the guy that does the occasional transfer. The guy in Welkom will never be part of a critical care retrieval team but he is still going to do a ventilated neonatal transfer because there may not always be a critical care retrieval team available. He is probably not going to go and do a PG dip as he maybe sees one neonate in 2 months. You cannot teach everything in 6 moths but you can teach enough to make a difference.

**Researcher 1**

Do I understand you correct in saying that it is like a progression from the six month course where you cover more basics and base knowledge. Then it moves on to the 1 year post grad diploma for the guys that will be ending up on the specialist retrieval teams.

**Participant 3**

Yes

**Researcher 1**

On the methods of assessment. What do you think is an adequate way to assess these people with the suggested additional training?

**Participant 3**

Ideally you would want to put them in charge of a real patient on a critical care retrieval unit with a mentor overseeing it. You might not exactly see the correct patient population. Another way might be to give them a simulated patient transfer of a real baby in hospital and then you discuss it with them without moving the patient. You do need to practically put a baby on a ventilator and move them in an incubator, but for the complex cases you can in hospital go to a patient with transposition for example and then discuss how you would move them. Then the student can give you his plan. So a combination of simulated and real life stuff. And written. I do like oral for specialised care. I do think oral assessments are valuable.

**Researcher 1**

There is an emphasis with hands on practical experience with some form of mentorship that goes with it that may include discussions. It may not actually be the movement of the patient as there may not be a need or a safety factor. For the theory and base knowledge you mentioned written and oral examinations.

**Participant 3**

I would rate oral higher than written from an exam perspective. I think you can learn a lot more from a student’s knowledge from an oral exam.

**Researcher 1**

Can you get a more in depth understanding or probe more, or what is the reason?

**Participant 3**

Yes, you can gauge understanding and insight a whole lot more when compared to answers from a written test. You can engage with the student and elaborate on the scenario. I think for specialised type of training oral has a lot of value.

**Researcher 1**

From a student perspective do you think oral exams are intimidating, or at that level do you think they will be fine with getting the information out of them?

**Participant 3**

I think that at that level it shouldn’t really matter. If you had to put me in an oral assessment now I would prefer it because written is so much effort. As a student you can validate and explain your answer better where in a written it is more black and white.

**Researcher 2**

You are making a good point. What you want to do in an oral exam is to probe and try and figure out that this is knowledge that they can just regurgitate or that they actually have an understanding and you can move the assessment in a particular way which you can’t do with a written.

**Participant 3**

Yes

**Researcher 1**

What type of person do you thin should take part in this type of education? Personality wise or is there something that needs to be different about the person?

**Participant 3**

I want to say someone that wants to. That is a big thing that I see in teaching right now. The difficult thing is determining if the person is just doing it to put it on his CV or he is doing it because he is legit interested. The keen interest would be the core thing.

**Researcher 1**

I think that our barrier would be as you said earlier that companies might be unlikely to pay for this type of training, and it is unlikely to find someone that doesn’t want to be there.

**Participant 3**

That is valid. It is not like ACLS, where the company says we have ten spaces who wants to go.

**Researcher 1**

What is the most important consideration that the curriculum committee has to take into consideration when developing this curriculum?

**Participant 3**

We have a responsibility to our patients and we have to keep that in mind. The babies have their entire lives ahead of themselves. You can also be the reason why this kid ends up on a ventilator for the rest of their life.

END
